# Targeting sorting nexin 3 to treat pulmonary fibrosis by dual modulating Wnt/β-catenin signaling

**DOI:** 10.1038/s41419-025-08248-x

**Published:** 2026-01-15

**Authors:** Dinghu Ma, Wenjing Yu, Hang Zhou, Rongyuan Lin, Ximeng Sun, Mingxia Peng, Chenjia Lin, Haoyu Du, Yueqi Li, Huimin Liang, Duanping Sun, Peiqing Liu, Jing Lu

**Affiliations:** 1 National and Local United Engineering Lab of Druggability and New Drugs Evaluation, Guangdong Provincial Key Laboratory of New Drug Design and Evaluation, Guangdong Province Engineering Laboratory for Druggability and New Drug Evaluation, School of Pharmaceutical Sciences, Sun Yat-sen University, Guangzhou, 510006 P.R. China; 2 Guangdong Provincial Key Laboratory of Pharmaceutical Bioactive Substances, Center for Drug Research and Development, Guangdong Pharmaceutical University, Guangzhou, 510006 P.R. China

**Keywords:** Pharmacology, Pathogenesis, Respiratory tract diseases

## Abstract

Pulmonary fibrosis (PF) is a chronic progressive lung disorder characterized by overactivation of Wnt/β-catenin signaling and limited therapeutic efficacy. This study identifies sorting nexin 3 (SNX3), a retromer-associated protein, as a dual regulator of PF pathogenesis through coordinated molecular mechanisms. SNX3 is significantly upregulated in PF patients’ lungs and bleomycin-induced murine fibrotic models, with predominant localization in alveolar type 2 (AT2) epithelial cells correlating with β-catenin hyperactivation and fibrotic progression. Genetic ablation of SNX3 in AT2 cells attenuated Wnt/β-catenin signaling, collagen deposition, and pulmonary dysfunction, while SNX3 overexpression exacerbated these phenotypes. Mechanistic studies further elucidated two distinct SNX3-driven regulatory pathways. Wls is rescued by SNX3 from lysosomal degradation to sustain Wnt ligand secretion and canonical pathway activation. In addition to Wls, casein kinase 1α (CK-1α) is identified as a novel cargo protein for SNX3, which mediates its plasma membrane recruitment via Rab5a-dependent endosomal recycling, bypassing the β-catenin destruction complex, ultimately suppressing proteasomal degradation of β-catenin. This dual regulatory mechanism positions SNX3 as a master coordinator of both Wnt-dependent and -independent β-catenin signaling in PF. Furthermore, we screened inhibitors targeting SNX3 and identified a novel small molecule, LC4, which effectively ameliorated pulmonary dysfunction and reversed pulmonary fibrosis. Tetrahedral framework nucleic acids (TDNs), known for their excellent biocompatibility and drug delivery capacity, were utilized to develop a multifunctional nanodrug delivery system (TDN-LC4) to enhance the treatment of PF. By optimizing this loading approach, we improved LC4 delivery efficiency, enhanced its therapeutic potential, and minimized off-target effects. Our findings reveal SNX3 as a master coordinator of dual Wnt-dependent and -independent β-catenin activation, and propose TDN-LC4 as a potential therapeutic strategy to disrupt pathogenic signaling redundancy in PF pathogenesis.

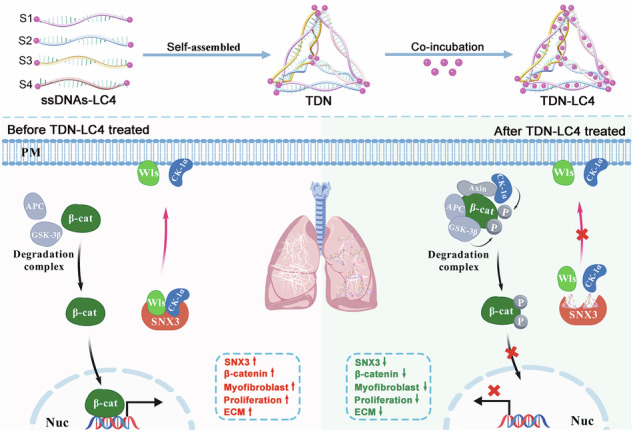

## Introduction

Pulmonary fibrosis (PF) is a chronic progressive disorder characterized by aberrant scar tissue deposition in the lungs, manifesting across diverse pulmonary diseases [1, 2]. The majority of PF cases are idiopathic, which means that their cause is unexplained [3, 4]. PF generally has an adverse prognosis, with a highly unpredictable progression that ultimately leads to irreversible damage, respiratory failure, and mortality [5–8].

Sorting nexin 3 (SNX3), belonging to SNXs family, is identified by our laboratory as a novel therapeutic target for heart failure [9, 10]. SNX3 interacts with vacuolar protein sorting (VPS) components VPS26, VPS35, and VPS29 to form the evolutionarily conserved SNX3-retromer complex, which orchestrates retrograde trafficking of cargo proteins to the trans-Golgi network (TGN), plasma membrane (PM), or nucleus, thereby modulating multiple pathological processes [11–13]. Our recent histological screening revealed a very high abundance of SNX3 in lung tissue in mice, in addition to heart tissue, suggesting its potential involvement in pulmonary pathologies.

SNX3-retromer complex mediates intracellular trafficking of key cargo proteins, such as signal transducer and activator of transcription 3 (STAT3), transferrin receptor (TFRC), and Wntless (Wls) [9, 10, 14, 15]. STAT3 translocation to nucleus and TFRC translocation to PM mediated by SNX3-retromer exacerbate myocardial injury and failure [9, 10]. Wls is recycled by SNX3-retromer to PM by rescuing it from the lysosomal degradation, which carries Wnt protein secretion and initiates Wnt/β-catenin signaling [14, 15]. However, the mechanism by which SNX3 regulates cargo proteins and its potential association with PF remains to be fully elucidated.

Wnt/β-catenin is a conserved cellular signaling pathway that plays a crucial role in the development of PF [16]. Following Wnt secretion mediated by Wls, extracellular Wnt binds to Frizzled receptor (along with the co-receptor LRP5/6), triggering PM recruitment of Dishevelled (Dvl), Axin and casein kinase 1α (CK-1α), thereby disrupting the β-catenin destruction complex [17, 18]. However, this signaling pathway involves significantly redundant or compensatory molecules, such as sFRP1 and DKK1 discovered by us [19, 20], and coexists with Wnt-independent β-catenin activation mechanisms [18, 21]. Therefore, the intervention of multiple targets of Wnt/β-catenin pathway may have a better effect on its inhibition and treatment of PF.

In this study, we illustrate that SNX3 was elevated in lung tissues from patients with lung disease and in a mouse model of bleomycin (BLM) -induced PF. SNX3 selectively expressed in alveolar type 2 epithelial cells (AT2) was associated positively with Wnt/β-catenin, fibrotic progression, and pulmonary dysfunction. While SNX3-mediated Wls recycling promotes Wnt secretion and β-catenin-driven PF development, Wls overexpression fails to completely rescue the protective effects of SNX3 knockout in AT2 cells, indicating pathway redundancy. Through unbiased screening, we identified CK-1α as another key cargo protein for SNX3 involved in β-catenin activation. SNX3-mediated CK-1α PM trafficking rather than proteasomal degradation represents a novel Wnt-independent β-catenin regulatory axis in PF, positioning SNX3 as a promising therapeutic target through dual modulation of β-catenin signaling.

Currently, treatment options for PF remain limited, and most research focuses on the development of anti-fibrotic drugs [22–24]. The two most commonly used drugs for the treatment of PF are pirfenidone and nintedanib, both of which have been approved for clinical use [23, 25, 26]. These drugs help slow the progression of fibrosis, improve lung function, and improve the quality of life of patients [23, 27]. Despite their efficacy, the clinical outcomes of pirfenidone and nintedanib are not ideal, and their side effects are notable [25–27]. Therefore, drug development for PF remains among the most pressing challenges.

Tetrahedral framework nucleic acids (TDNs), with their unique self-assembly properties, high tunability, excellent biocompatibility, and drug delivery capacity, have shown great potential in treating a variety of diseases [28, 29]. TDNs can function as drug delivery systems and gene carriers, and they have the ability to target specific cells or tissues, thereby enhancing therapeutic outcomes [30, 31]. Such as nucleic acid drug delivery (siRNA, microRNA, and DNA-based drugs [32–34]), small molecule drugs delivery (Doxorubicin, mitoxantrone, and platinum-based drugs [35–37]), and Traditional Chinese medicine monomers delivery (puerarin, curcumin, and resveratrol [31, 38]). In the context of PF, TDNs have been used as carriers to deliver traditional anti-fibrotic drugs, such as pirfenidone and nintedanib, through targeted delivery to the lungs [38, 39].

Guided by structural insights into SNX3-retromer-cargo interactions [9, 10, 15, 40, 41], we developed LC4, a novel small-molecule PF inhibitor identified through systematic compound screening and optimization. We designed a multifunctional nanodrug delivery system (TDN-LC4) to enhance the delivery efficiency of LC4 and minimize its side effects. This system utilizes two strategies to increase drug loading and enhance utilization. The “weapon-holding” strategy involves the direct chemical binding of LC4 with modified DNA single strands through chemical reactions, while the “armor-wearing” strategy exploits π-π stacking interactions between the drug molecules and the bases of the DNA tetrahedral double-helix structure. This integrated approach significantly enhances drug payload and shows a better effect on the inhibition of PF while minimizing off-target effects.

In summary, our work identifies SNX3 as a novel potential therapeutic target through dual β-catenin pathway modulation, develops LC4 as a novel inhibitor, and establishes TDN-LC4 as a precision nanomedicine for PF treatment.

## Methods and Materials

### Acquisition of microarray data

The Gene Expression Omnibus (GEO) database (http://www.ncbi.nlm.nih.gov/geo/) provided microarray datasets GSE76808 carried out on the [HG-U133A-2] Affymetrix Human Genome U133A 2.0 Array (GPL571 platform).

### Animal studies and ethics statement

The Shanghai Model Organisms Center created *Snx3-floxed* mice by CRISPR/Cas9 technology. *Snx3*^*flox/flox*^ mice were hybridized with C57BL/6J*Smoc-Sftpc*^*em(IRES-CreERT2)Smoc*^ mice (Stock No. NM-KI-18020. MGI ID: 109517) to produce *Sftpc-CreERT2* + ; *Snx3*^*flox/flox*^ *mice, i. e*. AT2 cells-specific *Snx3-cKO* mice. The Shanghai Model Organisms Center created *Snx3* transgenic mice by following established procedures. AT2 cells-specific overexpressed *Snx3* (*Snx3-cTg*) mice were created by crossing the two transgenic lines with C57BL/6J*Smoc-Sftpc* ^*em(IRES-CreERT2)Smoc*^ mice (Stock No. NM-KI-18020. MGI ID: 109517) (Tables S1–S3).

In 2 months aged adult transgenic female and male mice, tamoxifen (5 mg, Sigma-Aldrich, #T5648) diluted in 100 μL corn oil was injected to induce *Cre* gene recombination. Littermate control mice only received corn oil. This was done over 2 weeks, 7 times. After treatments, SNX3 was measured in *Snx3-cKO* mice and *Snx3-cTg* mice.

*Snx3-cKO* and *Snx3-cTg* mice were genotyped by PCR (Tables S4–S9). As we previously described, whole body plethysmography, and WBP (such as lung function and physiological indicators of respiration) were assessed using an EMKA small animal lung function testing system (EMKA, WBP-4A, France). The respiratory physiological indexes of animals in the awake and free state of activity. The evaluation of animal bronchial contraction and other parameters. Peak inspiratory flow (PIF), Enhanced pause (Penh), Minute volume (MV), were commonly used parameters that reflect lung function and airway patency. After that, mice were sacrificed and the lungs were quickly removed for further testing.

### Bleomycin-induced fibrosis model and AAV6 transduction

Pentobarbital sodium (150 mg/kg, Merck, #P3761) was used to anesthetize the mice before the intratracheal injection of 3 mg/kg BLM (Macklin, #B802467) diluted in 100 μL of saline with a 1 mL sterile syringe (OUJIAN MEDICAL, Zhejiang, China). As controls, mice received the same amount of saline intratracheal injection.

Adeno-associated virus 6 (AAV6) vectors, AAV6-Wls and AAV6-CK-1α with SP-C promoter for gene over-expression were constructed by Vigene. The AAV6 was intratracheally instilled before bleomycin injection 2 weeks.

### Primary alveolar type 2 epithelial cells (AT2s) and primary fibroblasts cells (FBs)

Six to eight-week-old mice were selected. First, sacrifice the mice by cervical dislocation. Subsequently, immerse the sacrificed mice in 75% alcohol for several minutes to conduct surface disinfection.

After that, carefully remove the lung tissue together with the trachea from the disinfected mice. Then, rinse the obtained lung tissue with PBS to eliminate any impurities on its surface. Next, use a syringe to slowly inject an appropriate amount of digestion solution, which contains 0.1% trypsin (Sigma, USA, #T4799-5G) and 0.1% collagenase Type I (Sigma, USA, #C0130), into the lungs through the trachea. This injection should be done in a way that makes the lung tissue fully expand. Then, place the lung tissue in an environment at 37 °C for digestion, which lasts for 30 min. During this digestion period, gently shake the container to ensure that the digestion is more thorough. Once the digestion is completed, rinse the lung tissue with PBS again. Then, collect both the digestion solution and the rinse solution into a centrifuge tube. Centrifuge the solution in the tube at 1500 rpm for 10 min. After centrifugation, discard the supernatant and resuspend in DMEM/F12 (Procell, China, #PM150312) containing 10% FBS (ABW, USA, #0050S). For cell purification, the differential adhesion method can be employed. Inoculate the cell suspension obtained above into a cell culture plate. Then, place the culture plate in an incubator at 37 °C with 5% CO₂ for incubation. After 2 h of incubation, adherent cells such as fibroblasts will adhere to the wall of the culture plate first, while the non-adherent cells are mainly alveolar type II epithelial cells. Cell purity was confirmed by quantifying AT2-specific markers (SP-C) through bright-field microscopy and immunofluorescence (IF) (Fig. S3K).

### Micro-CT

The lung tissue density of the mice was measured by using PET/CT (Inveon, Germany) after the BLM model, and level of PF in each group was assessed. The mice were fixed with tape after being given anesthesia, and then the mice were maintained in the scanning area. The mice were then subjected to a CT plain scan using the following procedures: the mice’s living lung tissue was scanned after setting parameters. To assess the variation in the level of PF in mice, the structural and morphological variations in lung tissue were observed based on the images that were obtained.

### Lung tissues transmission electron microscopy

Lung tissues were taken from the left lobes and were promptly preserved at room temperature in glutaraldehyde solution (China, Servicebio, #P1126) for 2 h in the dark. After that, the lung tissues were fixed, sliced, and stained for imaging. A JEM-1400 transmission electron microscope (JEOL, Japan) was used to examine the ultrastructure.

### Lung tissue processing

The left lobe was fixed in 10% paraformaldehyde and then sliced. To examine histopathological changes in the slices, HE, PSR, IHC, and IF staining were applied. For IHC and IF staining, paraffin sections of left lung tissue samples were incubated overnight at 4 °C with primary antibodies: anti-SNX3 (Servicebio, China, diluted 1:200, #K009182P), anti-α-SMA (Proteintech, China, diluted 1:200, #23081-1-AP), anti-SP-C (Proteintech, China, diluted 1:200, #10774-1-AP), anti-CD31 (Servicebio, China, diluted 1:200, #GB15063), anti-CD68 (Servicebio, China, diluted 1:200, #GB11309), anti-collagen 1 (Proteintech, China, diluted 1:200, #66761-1-Ig), anti-Wls (Proteintech, China, diluted 1:200, #23081-1-AP), anti-CK-1α (Santa Cruz, USA, diluted 1:200, #SC-74582), and anti-β-catenin (Proteintech, China, diluted 1:200, #51667-2-AP). After incubation, the samples were exposed to anti-CY3-tyramide (Servicebio, China, diluted 1:500, #G1223), anti-FITC-tyramide (Servicebio, China, diluted 1:500, #G1222), and counterstained with 4′,6-diamidino-2-phenylindole (DAPI, Servicebio, China, #G1012). Samples were captured using an EVOS M7000 (Thermo Fisher, USA). The quantitative analysis of IHC staining was performed using ImageJ software.

### Hydroxyproline concentration

The lung tissues were harvested and the concentration of hydroxyproline was detected by Hydroxyproline assay kit (Nanjing Jian cheng, China, #A030-2-1).

### Recombinant adenoviral vectors and virus infections

Standard methods were used to create recombinant adenoviral vectors that expressed the sh-SNX3 sequence, Ad-SNX3 sequence as reported. Ad-Wls, or Ad-CK-1α were constructed by Vigene. The concentration of each purified protein was determined, and the protein was dissolved in PBS and kept at −80 °C. AT2s and FBs were treated with recombinant adenoviral vectors and virus (including Ad-NC, Ad-SNX3, sh-NC, sh-SNX3, Ad-Wls, or Ad-CK-1α) for 48 h before being separated for further analysis.

### Immunofluorescence staining and microscopy

AT2s and FBs were seeded onto 15 mm confocal dishes at an appropriate density. The cells were starved in FBS-free medium for 6 h. After 24 h, AT2s and FBs were cultured in medium containing TGF-β1 (10 ng/mL) and 1% FBS. Following TGF-β1 stimulation, the cells were washed three times with PBS, fixed in 4% paraformaldehyde for 10 min, and permeabilized with 0.3% Triton X-100 for 10 min. Goat serum was then used to block the cells for 1 h. The cells were incubated with primary antibodies overnight at 4 °C. The following primary antibodies were used: anti-SNX3 (Proteintech, China, diluted 1:200, #10772-1-AP), anti-SNX3 (Santa Cruz, USA, diluted 1:200, #sc-376667), anti-α-SMA (Proteintech, China, diluted 1:200, #23081-1-AP), anti-ACTA2 (Proteintech, China, diluted 1:200, #14395-1-AP), anti-SP-C (Proteintech, China, diluted 1:200, #10774-1-AP), anti-Wls (Proteintech, China, diluted 1:200, #23081-1-AP), anti-CK-1α (Abcam, USA, diluted 1:200, #EPR19824), anti-β-catenin (Proteintech, China, diluted 1:200, #51667-2-AP), anti-PSMC3 (Cloud-Clone, China, diluted 1:200, #PAG278Hu01), anti-Rab 5A (CST, USA, diluted 1:200, #12666), and anti-LAMP1 (CST, USA, diluted 1:200, #9091). After overnight incubation, the cells were treated with Alexa Fluor 488/594-conjugated secondary antibodies (CST, USA, diluted 1:100, #4408S, #4412S, #8889S, #8890S) for visualization. The cell nucleus was stained with DAPI (CST, USA, diluted 1:1000, #4083). Images were captured using an FV3000 fluorescence microscope (Olympus, Japan). Colocalization analysis of IF staining was performed using ImageJ software.

### Western blotting and coimmunoprecipitation (co-IP) assays

Following processing with RIPA buffer (Beyotime, China, #P0013B) containing a combination of phosphatase and protease inhibitors (Bimake, USA, #B14012 and #B15001) to extract the lysate, the lung tissues were centrifuged. The samples were then loaded in equal volumes, separated by SDS–PAGE, transferred to PVDF membranes (Millipore, Germany), and blocked with 5% skim milk. The primary antibodies were incubated with the immunoblots overnight at 4 °C, and the appropriate secondary antibodies were added for an additional hour at room temperature. Protein levels were measured using an electrochemiluminescence system (Tanon, China), and the data were quantitatively analyzed using ImageJ software. For co-immunoprecipitation (co-IP), lung tissue homogenates were processed with immunoprecipitation lysis buffer (Beyotime, China, #P0013). After centrifugation, the appropriate primary antibodies were applied to the cell lysates, which were rotated overnight at 4 °C, followed by incubation with Pierce™ Protein A/G Agarose for 4 h at 4 °C. The target proteins were then eluted and analyzed by Western blotting. The primary antibodies used were as follows: anti-SNX3 (Proteintech, China, diluted 1:1000, #10772-1-AP), anti-FN (Proteintech, China, diluted 1:1000, #66761-1-Ig), anti-α-SMA (Proteintech, China, diluted 1:1000, #23081-1-AP), anti-Wls (Proteintech, China, diluted 1:1000, #23081-1-AP), anti-CK-1α (Abcam, USA, diluted 1:1000, #EPR19824), anti-β-catenin (Proteintech, China, diluted 1:1000, #51667-2-AP), anti-GAPDH (Proteintech, China, diluted 1:2500, #10494-1-AP), anti-Flag (Merck, USA, diluted 1:2500, #F1804), anti-Lamin B1 (Proteintech, China, diluted 1:2500, #12987-1-AP), anti-VPS35 (Proteintech, China, diluted 1:1000, #10236-1-AP), anti-VPS26 (Proteintech, China, diluted 1:1000, #12804-1-AP), and anti-GFP (Proteintech, China, diluted 1:1000, #66002-1-AP).

### Wound-healing assay

AT2s and FBs were seeded in 6-well plates at the proper density and cultured in medium to 90% confluence. To remove cell waste and detached cells, the cell monolayer was scratched straight across with a sterile 1 mL tip. After culture in FBS-free medium for 12–24 h, migration images were gathered to calculate healing areas.

### EdU fluorescence staining

The BeyoClick™ EdU Cell Proliferation Kit with DAB (Beyotime, China, #C0085S) was employed to identify cell proliferation. Twenty-four-well plates were used to cultivate AT2s and FBs. Following treatment, the test was carried out in compliance with the product manual.

### Synthesis and characterization of TDN-LC4

The four ssDNAs identified in the initial step (Sangon, Shanghai, China), which are listed in Table S1, were dissolved in a Tris-magnesium sulfate (TM) annealing buffer solution (10 mM Tris, 5 mM MgCl₂, pH 8.0, Beyotime, China). The ssDNAs-LC4 complexes were individually synthesized by reacting LC4 with ssDNA in the presence of HATU and DIPEA. The mixture was then heated at 25 °C for 120 min and gradually cooled to 4 °C. In the second step, the TDN was synthesized using the four modified single-stranded LC4-DNAs. Briefly, equimolar quantities of LC4-DNAs were denatured at 95 °C for 10 min and then cooled to 4 °C for 20 min, allowing LC4 to associate with TDN/Cy5-TDN. In the third step, LC4 was added to the TDN/Cy5-TDN solution, and the mixtures were co-incubated for 8 h at 4 °C. After incubation, the mixture was ultrafiltered to purify the TDN-LC4 complex. The formation of TDN-LC4 was characterized by 3.5% agarose gel electrophoresis, run at a constant voltage of 110 V for 40 min. The gel was scanned under UV illumination. The absorption spectra of TDN, LC4, and TDN-LC4 were measured using a spectrophotometer. For detailed morphological and dimensional analysis of TDN-LC4, transmission electron microscopy (TEM, Tecnai, Netherlands) was employed. Additionally, dynamic light scattering (DLS) was used to assess the ζ-potential of both TDN and TDN-LC4.

### Uptake of TDN and TDN-LC4 and Stability

To confirm the absorption, intracellular trafficking, and localization of TDN and TDN-LC4 by the cells, Cy5 labeling was used to track the presence of TDN and TDN-LC4. AT2s and FBs were separately co-incubated with Cy5-TDN (200 nM) and Cy5-TDN-LC4 (20 μM LC4, 200 nM Cy5-TDN) for 6 and 12 h. Images were then obtained using laser confocal microscopy. For the stability characterization of TDN-LC4, TDN-LC4 (20 μM LC4, 200 nM Cy5-TDN) was combined with cell lysate in medium and incubated at 37 °C for 0, 3, 6, 9, 12, and 24 h. After incubation, the samples were analyzed using 3.5% agarose gel electrophoresis.

### TDN-LC4 treatment

*N-Tg* mice *and Snx3-cTg* mice were selected. Pentobarbital sodium was used to anesthetize the mice before the intratracheal injection of 3 mg/kg BLM diluted in 100 μL of saline. As controls, mice received the same amount of saline intratracheal injection. At the same time, LC4 (10 mg/kg) dissolved at 3% DMSO, TDN-LC4 (LC4: 10 mg/kg, TDN: 1 μM) and pirfenidone (10 mg/kg) were administered for 3 weeks. As controls, mice received the same amount of TDN intratracheal injection. For the chronic toxicity experiment, a 10-fold dose of TDN-LC4 (LC4: 100 mg/kg, TDN: 10 μM) was administered via tracheal instillation, once every 2 days for 1 month. After that, mice were sacrificed and quickly removed for further testing.

### Bioluminescence imaging

Bioluminescence imaging utilizing the near-infrared fluorescent dye Cy5 (excitation/emission: 649/670 nm) was employed to track distribution [34, 42, 43]. Mice, pre-treated with either bleomycin (BLM) or saline, received Cy5-TDN-LC4 via intratracheal instillation. At designated time points (0.5, 1, 3, 6, 12, 24, and 48 h post-administration), mice were euthanized, followed by the collection of major organs. The in vivo imaging was performed by a luminescent imaging system (Shanghai United Digital Biotech. Co. Ltd., NIR-II-ST).

### Total RNA isolation, cDNA synthesis, and real-time polymerase chain reaction (qPCR)

Total RNA was isolated from tissues or cells using TRIzol reagent per manufacturer’s instructions and quantified spectrophotometrically. cDNA was synthesized from 1 µg RNA using a reverse transcriptase kit with oligo(dT) primers and random hexamers. qPCR was performed using SYBR Green master mix on a QuantStudio 5 system in 20 µL reactions containing cDNA and gene-specific primers. Samples were run in triplicate with melting curve analysis. Gene expression was normalized to endogenous reference genes using the 2^−ΔΔCt^ method.

### Pharmacokinetic profiling

Pharmacokinetic profiles of TDN-LC4 and pirfenidone were comparatively assessed following administration. Serial blood samples were collected via designated methods at predetermined time points post-dose. Plasma was separated immediately by centrifugation and stored at −80 °C until analysis. Lung tissues and other relevant organs were harvested at specified time points, homogenized in appropriate buffer, and centrifuged to obtain supernatants. Concentrations of TDN-LC4 and pirfenidone in plasma and tissue homogenates were quantified using validated liquid chromatography-tandem mass spectrometry (LC-MS/MS) methods. Non-compartmental pharmacokinetic analysis was performed using Phoenix WinNonlin software to calculate key parameters including area under the concentration-time curve (AUC_0-48h_), maximum plasma concentration (C_max_), time to C_max_ (T_max_), elimination half-life (t_1/2_), and tissue-to-plasma concentration ratios. Statistical comparisons between TDN-LC4 and pirfenidone pharmacokinetic parameters were conducted using appropriate tests (e.g., Student’s *t*-test or ANOVA).

### Blood biochemistry analysis

Serum levels of alanine aminotransferase (ALT), aspartate aminotransferase (AST), alkaline phosphatase (ALP), blood urea nitrogen (BUN), creatinine (CRE) and uric acid (UA) were measured using the HITACHI 7100 automated biochemical analyzer.

### Duolink Proximity Ligation Assays (PLA)

AT2s were cultured overnight on confocal dishes for 48 h. After treatment, the cells were fixed with 4% paraformaldehyde for 10 min and permeabilized with 0.3% Triton X-100 for 10 min at room temperature. Then, cells were blocked with PLA blocking buffer for 1 h at 37 °C and incubated with rabbit anti-Wls (Proteintech, China, #23081-1-AP), anti-CK-1α (Abcam, USA, #EPR19824) and mouse anti-SNX3 (Santa Cruz, USA, diluted 1:200, #sc-376667) overnight at 4 °C. AT2s were added to the Duolink® In Situ Red Starter Mouse/Rabbit kit (DUO92101; Sigma) following the manuals [10, 44, 45]. The cell nucleus was stained with DAPI (CST, USA, diluted 1:1000, #4083). The PLA signals were observed were captured using an FV3000 fluorescence microscope (Olympus, Japan).

### Statistical analysis

All results were analyzed using the GraphPad Prism software (version 8.0.2) and displayed as means ± SEM. According to what is stated in the figure legends, statistical analysis was done. In addition, fluorescence colocalization assays coefficients were processed using Image J software. Student’s *t*-test and Mann-Whitney test were used for significant differences between two groups. All data (more than two groups) were analyzed using with ANOVA test. The non-parametric test Kruskal–Walli test followed by the Dunn’s post-hot test was used to correct for multiple comparisons. In each case, differences were regarded as statistically significant at *P* < 0.05. *P < 0.05, **P < 0.01, ***P < 0.001 or ^#^*P* < 0.05, ^##^*P* < 0.01, ^###^*P* < 0.001 or ^&^*P* < 0.05, ^&&^*P* < 0.01, ^&&&^*P* < 0.001.

## Results

### SNX3 expression was upregulated in pulmonary fibrosis

**Fig. 1:**
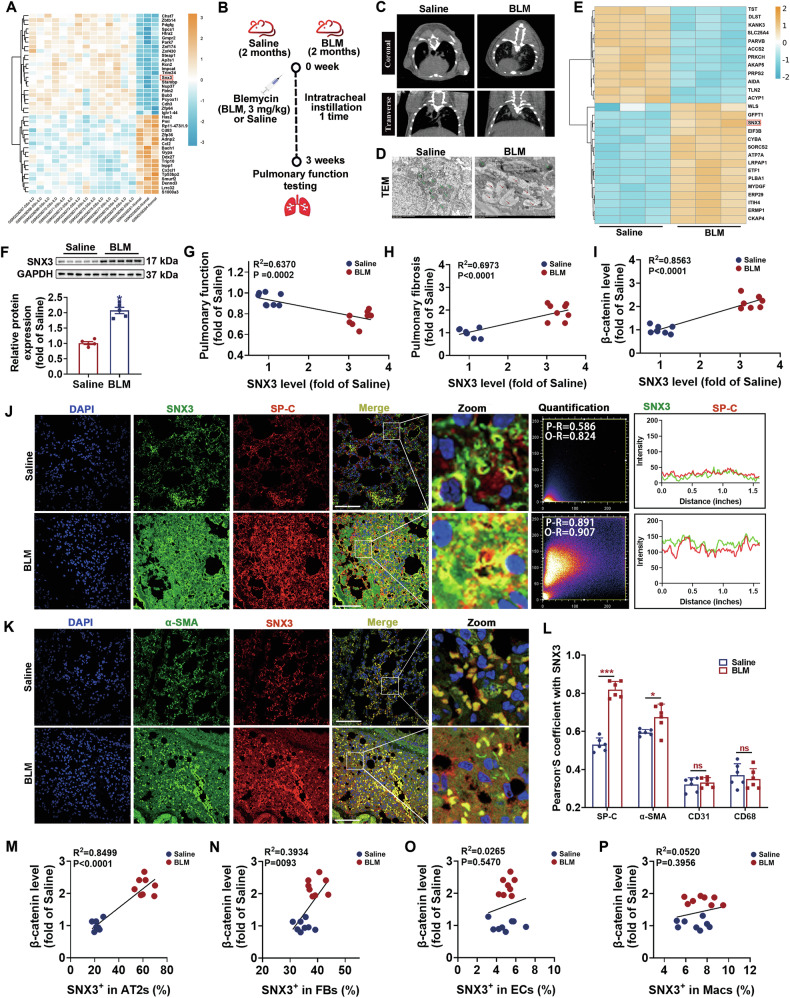
SNX3 expression was upregulated in pulmonary fibrosis. **A** Heatmap of the abundance of genes determined by bioinformatics analysis from GSE76808. **B** The flow chart of BLM-induced PF in C57BL/6 mice. Were created with BioGDP.com. **C** Micro-CT images were shown; *n* = 5 mice. **D** The ultrastructural dysfunction of lung tissues was detected by TEM analysis; Scale bars: 2 μm. **E** Heatmap of various proteins determined by proteomics analysis of the lungs of 3 saline-treated mice and 3 BLM-treated mice. **F** Representative images of western blotting analysis detected the protein level of SNX3; *n* = 8 mice. **G** Correlation between SNX3 protein levels and pulmonary function (Pearson *R*^2^ = 0.6370; *P* = 0.0002). **H** Correlation between SNX3 protein levels and pulmonary fibrosis (Pearson *R*^2^ = 0.6973; *P* < 0.0001). **I** Correlation between SNX3 protein levels and β-catenin protein levels (Pearson *R*^2^ = 0.8563; *P* < 0.0001). **J**, **K** The change of colocalization of SNX3 with SP-C and α-SMA in mice was measured by IF staining analysis; Scale bar: 100 μm, *n* = 8 mice. **L** The change Pearson^,^S colocalization coefficient of SNX3 with SP-C, α-SMA, CD31, CD68 in mice was shown; *n* = 6 mice. **M** Correlation between β-catenin protein levels and SNX3 positive rate in AT2s (Pearson *R*^2^ = 0.8499; *P* < 0.0001). **N** Correlation between β-catenin protein levels and SNX3 positive rate in FBs (Pearson *R*^2^ = 0.3934; *P* < 0.0093). **O** Correlation between β-catenin protein levels and SNX3 positive rate in ECs (Pearson *R*^2^ = 0.0265; *P* = 0.5470). **P** Correlation between β-catenin protein levels and SNX3 positive rate in Macs (Pearson *R*^2^ = 0.0520; *P* = 0.3956). The data were shown as means ± SEM. *P < 0.05 vs. Saline group. ns, not significant.

To investigate the potential role of SNX3 in the onset of PF, we analyzed a total of 14 lung injury tissues and 4 matched normal lung tissues from the GSE76808 dataset, using the adj. *p* < 0.05 and |log FC | > 1 as cutoff values. Twenty-four differentially expressed genes (DEGs) were significantly increased, while 18 were downregulated. The expression of *Snx3* was increased in lung disease tissues compared with healthy controls (Fig. 1A). To further explore the pathological process of pulmonary fibrosis (PF), we established a mouse model of PF through intratracheal injections of bleomycin (BLM) (Fig. 1B), which was validated by several results (Figs. 1C, D, and S1A–K). Proteomic analysis of the lungs of BLM-induced mice showed that 318 proteins were upregulated and 323 were downregulated (Fig. 1E). Among these, SNX3 protein levels were elevated in BLM-induced lung tissues (Fig. 1F). The increase in the protein level of SNX3 induced by BLM was confirmed by immunofluorescence staining (IF) and immunohistochemistry (IHC) staining (Fig. S2A, B). The SNX3 protein level was negatively correlated with pulmonary function and positively correlated with fibrosis area (Fig. 1G, H).

In view of the central role of Wnt/β-catenin in PF and the fact that Wls, which has the function of mediating Wnt secretion, is a recognized cargo protein of SNX3 [9, 46–49], we also examined the correlation between SNX3 and β-catenin in the process of PF. β-catenin was upregulated in BLM-induced mice (Fig. S2C, D). SNX3 was positively correlated with β-catenin protein levels (Fig. 1I). In addition, the same effects were also observed in cell model of PF (Fig. S3A–J).

Next, we examined the expression of SNX3 in cells within lung sections from BLM-induced and saline-treated mice. The results of the IF staining showed that SNX3 co-localized with SP-C (AT2 cell marker), α-SMA (myofibroblast marker), CD31 (endothelial cell marker), and CD68 (macrophage marker). However, in BLM-induced mice, SNX3 showed higher levels of co-localization specifically with AT2 cells, rather than the other cell types (Fig. 1J–L and Fig. S2F, G). Additionally, SNX3 was more highly positively correlated with β-catenin protein levels in AT2 cells rather than others (Fig. 1M–P). These findings suggest that SNX3 in AT2 cells is associated with Wnt/β-catenin signaling pathway in the PF process.

### SNX3 deficiency alleviated pulmonary fibrosis

**Fig. 2:**
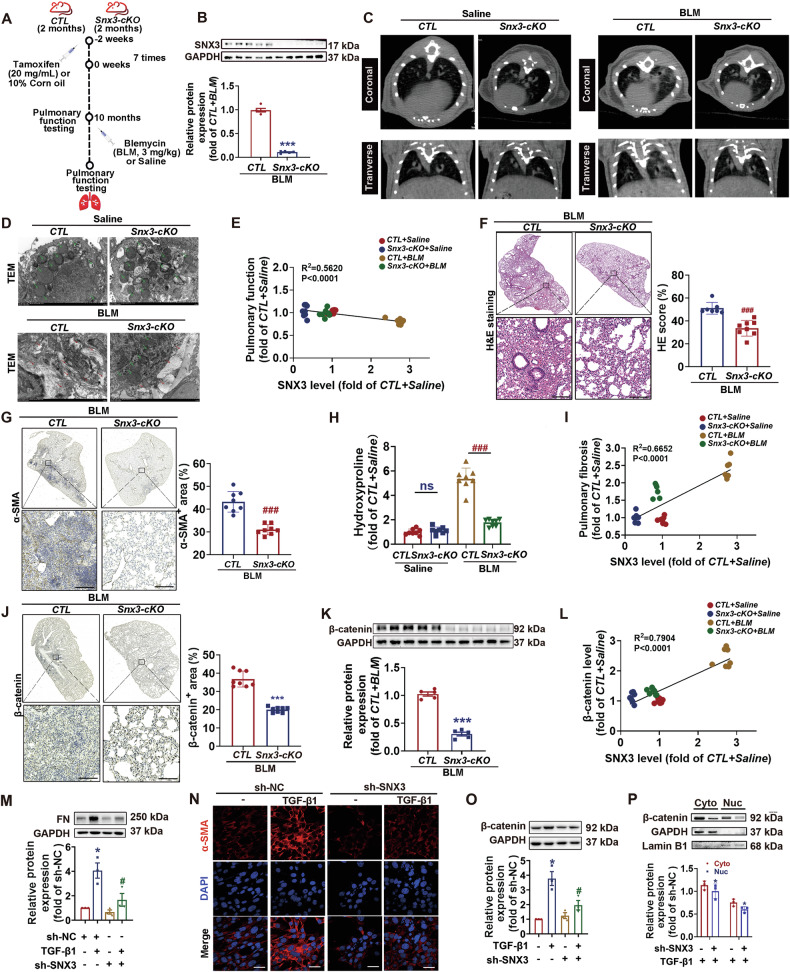
SNX3 deficiency alleviated pulmonary fibrosis. C57BL/6 *Snx3-cKO* mice and *CTL* mice at 2 months were administered tamoxifen (3 mg/mL, *i.p* injection, 7 times) or 10% corn oil for 2 weeks, and were administered 3 mg/kg BLM (intratracheal instillation) or saline one time. The alterations in pulmonary structure and lung function were examined. **A** Flow chart of *Snx3-cKO* construction and BLM-induced PF models. Were created with BioGDP.com. **B** The protein level of SNX3 was measured by western blotting analysis, *n* = 8 mice. **C** Micro-CT images of *Snx3-cKO* mice were shown; *n* = 6 mice. **D** The ultrastructural dysfunction of the lung tissues of *Snx3-cKO* mice was detected by TEM analysis; Scale bars: 2 μm. **E** Correlation between SNX3 levels and pulmonary function (Pearson *R*^2^ = 0.5620; *P* < 0.0001). **F**, **G** Representative images of HE staining and α-SMA images were shown; Scale bar: 200 μm, *n* = 8 mice. **H** Representative images of hydroxyproline concentration were shown, *n* = 8 mice. **I** Correlation between the SNX3 protein level and pulmonary fibrosis (Pearson *R*^2^ = 0.6652; *P* < 0.0001). **J**, **K** The protein level of β-catenin in *Snx3-cKO* mice was detected by Western blotting analysis (*n* = 8 mice) and IHC staining analysis (Scale bar = 200 μm; *n* = 8 mice). **L** Correlation between SNX3 protein levels and β-catenin protein levels (Pearson *R*^2^ = 0.7904; P < 0.0001). **M** sh-SNX3 inhibited the protein expression of FN caused by TGF-β1 in AT2 cells; *n* = 3 experiments. **N** IF staining analysis demonstrated α-SMA protein levels in AT2 cells; Scale bar: 25 μm, *n* = 3 experiments. **O**, **P** Western blotting analysis was used to identify the protein level and nuclear distribution of β-catenin, *n* = 3 experiments. The data were shown as means ± SEM. *P < 0.05 vs. *CTL+ Saline group or* sh-NC. ^#^*P* < 0.05 vs *CTL* + *BLM group or* sh-NC + TGF-β1. ns, not significant.

To study the role of SNX3 in PF, a AT2 cells specific *Snx3-cKO* mouse model was produced by mating *Snx3-floxed* mice with the C57BL/6J *Smoc-Sftpc*^*em(IRES-CreERT2)Smoc*^ mouse line and given tamoxifen to induce *CreERT2* recombinase expression, and a PF mouse model was established via intratracheal injections of BLM (Fig. 2 and Fig. S4A–E). The endogenous SNX3 protein was markedly reduced in the lungs of *Snx3-cKO* mice, compared to their littermate negative controls (*CTL*) (Fig. 2B).

Under standardized conditions, *Snx3-cKO* mice exhibited no glaring structural or functional deficiencies in the lung (Fig. S4F). Under BLM-induced conditions, the increased lung shadow area, mitochondrial disorders, thickened alveolar septa and an accumulation of collagen fibrils and elastic fibers were significantly ameliorated in *Snx3-cKO* mice (Figs. 2C, D and S4G–K). The SNX3 protein level was negatively correlated with pulmonary function (Fig. 2E). Compared to BLM-induced *CTL* mice, *Snx3-cKO* mice had significantly improved pulmonary appearance, fibrosis areas, collagen fibrils, elastic fibers, hydroxyproline concentration and lung dysfunction (Fig. 2F–H and Fig. S4L–T). The SNX3 protein level was positively correlated with pulmonary fibrosis (Fig. 2I). β-catenin protein level was significantly reduced in BLM-treated *Snx3-cKO* mice (Fig. 2J, K) and was positively correlated with SNX3 protein expression (Fig. 2L). Changes in β-catenin and protective effects against of PF by SNX3 knockout were also observed in sh-SNX3 infected AT2 and FB cells (Fig. 2M–P and Figs. S5A–I and S6A–D). These findings suggest that SNX3 knockout significantly reduced Wnt/β-catenin activation and lung dysfunction in PF.

### Overexpression of SNX3 caused pulmonary fibrosis

**Fig. 3:**
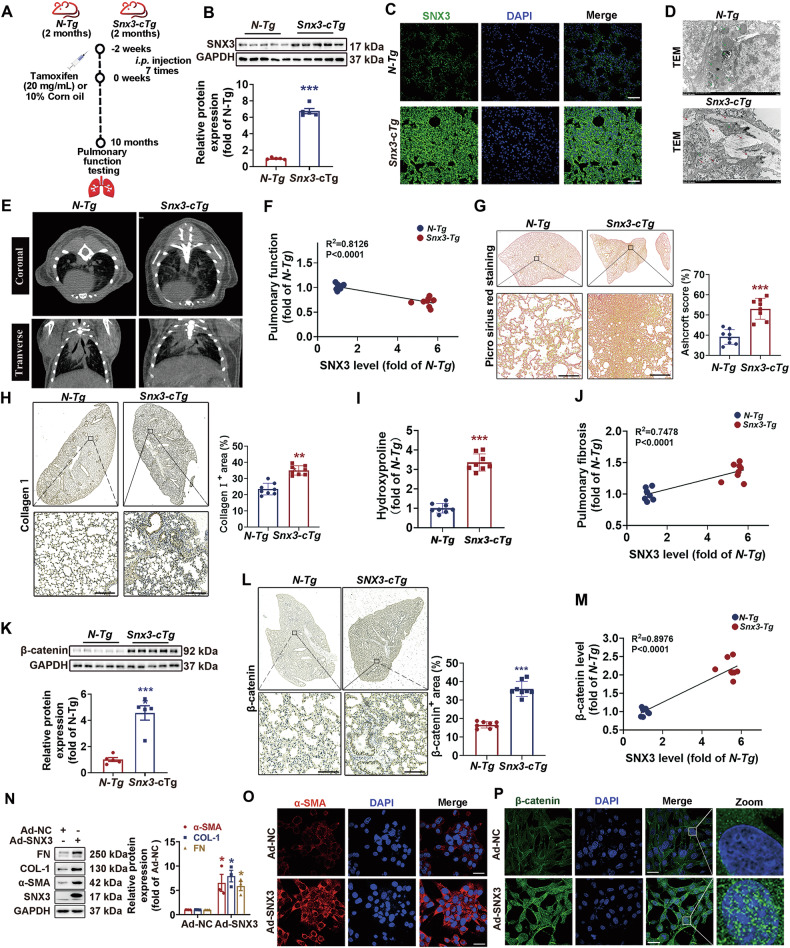
Overexpression of SNX3 caused pulmonary fibrosis. *Snx3-cTg* mice and *N-Tg* mice at 2 months were administered tamoxifen (3 mg/mL, i.p injection, 7 times) or 10% corn oil for 2 weeks, and were examined the alterations in pulmonary structure and lung function at 10 months. **A** *Snx3-cTg* construction flow chart. Were created with BioGDP.com. **B**, **C** Western blotting and IF staining analysis was used to detect the protein level of SNX3 in mouse lung tissues; Scale bar: 100 μm *n* = 8 mice. **D** TEM analysis of lung tissues from *Snx3-cTg* or *N-Tg* mice; Scale bars: 2 μm. **E** Micro-CT images were shown; *n* = 6 mice. **F** Correlation between SNX3 protein level and pulmonary function (Pearson *R*^2^ = 0.8126; *P* < 0.0001). **G** PSR staining were shown; Scale bar: 200 μm, *n* = 8 mice. **H** Collagen 1 was detected by IHC staining analysis; Scale bar: 200 μm, *n* = 8 mice. **I** Representative images of the hydroxyproline concentration were shown, *n* = 8 mice. **J** Correlation between SNX3 protein level and pulmonary fibrosis (Pearson *R*^2^ = 0.7478; *P* < 0.0001). **K**, **L** The protein level of β-catenin was detected by Western blotting analysis (*n* = 8 mice) and IHC staining analysis (Scale bar = 200 μm; *n* = 8 mice). **M** Correlation between SNX3 protein level and β-catenin level (Pearson *R*^2^ = 0.8976; *P* < 0.0001). **N** The protein levels of FN, COL-1, α-SMA and SNX3 in FBs were detected by western blotting analysis; *n* = 3 experiments. **O** α-SMA was measured by IF staining analysis; Scale bar: 100 μm, *n* = 3 experiments. **P** The protein level and nuclear distribution of β-catenin in AT2 cells were measured by IF staining analysis (Scale bar: 25 μm, *n* = 3 experiments). The data were shown as means ± SEM. *P < 0.05 vs. *N-Tg or* Ad-NC. ns, not significant.

To study whether SNX3 causes PF, AT2 cell specific *Snx3-cTg* mice were produced by crossing *Snx3* transgenic mice with *C57BL/6JSmoc-Sftpc*^*em(IRES-CreERT2)Smoc*^ mice and given tamoxifen to induce *CreERT2* recombinase expression at 2 months of age (Fig. 3A and Fig. S7A–G). As shown by western blotting and IHC staining, *Snx3-cTg* mice had higher SNX3 protein level in the lungs than in their littermate negative transgenic mice (*N-Tg*) (Fig. 3B, C).

Ten-month-old *Snx3-cTg* mice exhibited clear pulmonary dysfunction, as demonstrated by the observed results: (1) mitochondrial disorders, hypertrophic alveolar septa, and accumulation of collagen and elastic fiber according to TEM (Fig. 3D); (2) increased lung injury by Micro-CT and surface morphology (Figs. 3E and S7H). The level of the SNX3 protein was negatively correlated with pulmonary function (Fig. 3F), and *Snx3-cTg* mice exhibited spontaneous pulmonary dysfunction (Fig. S7I–L). Pulmonary fibrosis areas, deposition of collagen fibers, hydroxyproline concentration and increased fibrosis biomarkers were observed in *Snx3-cTg* mice (Figs. 3G–I and S7M–O). The SNX3 protein level was positively correlated with the degree of fibrosis (Fig. 3J). β-catenin protein level was upregulated in *Snx3-cTg* mice (Fig. 3K, L) and was positively correlated with SNX3 protein levels (Fig. 3M). The effects of fibrosis and β-catenin activation were also observed in Ad-SNX3 infected AT2 and FB cells (Figs. 3N–P, S8A–G and S9A–E). These results show that SNX3 overexpression significantly induced Wnt/β-catenin activation and the PF process.

### SNX3 interacted with Wls and regulated its intracellular recycling and degradation to activate Wnt/β-catenin pathway

**Fig. 4:**
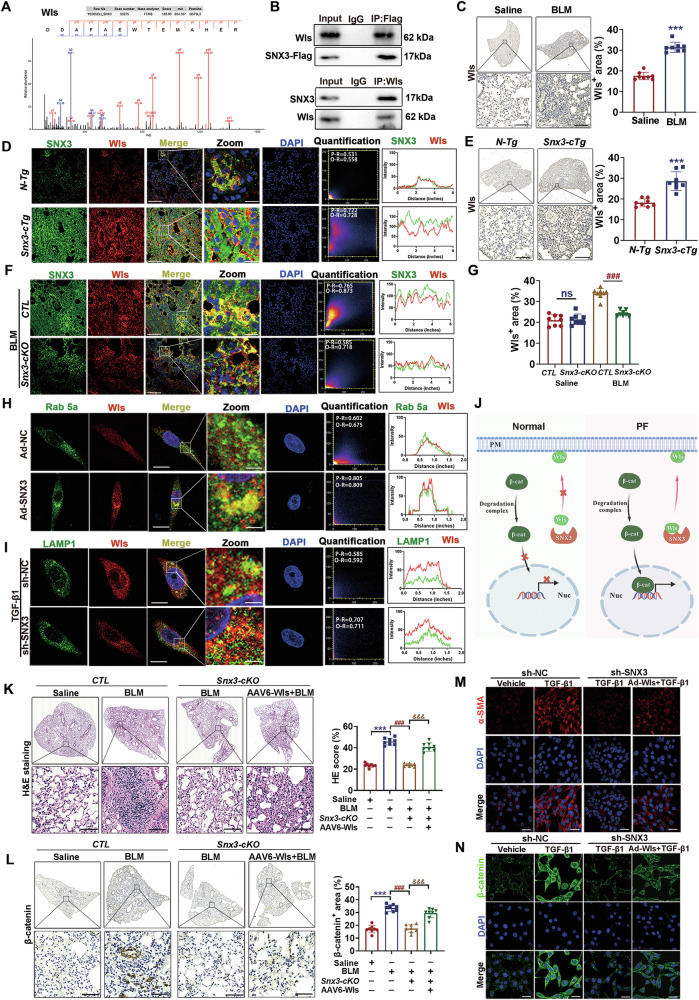
SNX3 interacted with Wls and regulated its intracellular recycling and degradation to activate Wnt/β-catenin pathway. **A** The protein mass spectrum of Wls were shown. **B** Fresh lung homogenates were sparked by anti-Flag or anti-Wls for Wls or SNX3 detection in co-IP analysis. **C** IHC staining analysis shown the Wls expression in BLM-induced mice (Scale bar: 200 μm; *n* = 8 mice). **D** The protein level and colocalization of Wls with SNX3 in *Snx3-cTg* mice were detected by IF staining analysis (Scale bar: 100 μm, *n* = 8 mice). **E** The protein level of Wls were detected by IHC staining analysis in *Snx3-cTg* mice (Scale bar: 200 μm; *n* = 8 mice). **F** The protein level and colocalization of Wls with SNX3 in *Snx3-cKO* mice were detected by IF staining analysis (Scale bar: 100 μm, *n* = 8 mice). **G** The protein level of Wls in *Snx3-cKO* mice were detected by western blotting analysis (*n* = 8 mice). **H**, **I** IF staining analysis were performed to detected the protein level and colocalization of Wls with Rab 5a and LAMP1; Scale bar: 10 μm; *n* = 3 experiments. **J** The Wls intracellular recycling and degradation flow chart. Were created with BioGDP.com **K** H&E staining and PSR staining in lung tissue sections were shown. Scale bar: 200 μm, *n* = 8 mice. **L** Representative images of IHC staining analysis in *Snx3-cKO* mice were shown; Scale bar: 200 μm, *n* = 8 mice. **M** IF staining analysis of α-SMA were shown; Scale bar: 25 μm, *n* = 3 experiments. **N** IF staining analysis of β-catenin were shown; Scale bar: 25 μm, *n* = 3 experiments. The data were shown as means ± SEM. *P < 0.05 vs. *CTL+ Saline* group *or* sh-NC. ^#^*P* < 0.05 vs *CTL* + *BLM* group *or* sh-NC + TGF-β1. ^&^*P* < 0.05 vs *Snx3-cKO* + *BLM* group *or* sh-SNX3 + TGF-β1. ns, not significant.

The notable effects of SNX3 on PF (Figs. 1–3 and S1–S9) encouraged us to examine the underlying mechanisms involved. The most recognized function of SNX3 is to mediate intracellular recycling of its cargo proteins [9, 48]. These cargo proteins include Wls, which is necessary for the secretion of Wnts into extracellular [15]. We proposed that SNX3 contributed to Wnt/β-catenin pathway and PF possibly by mediating the intracellular recycling of Wls. Hence, we confirmed that SNX3 interacted with Wls via IP-MS, co-IP and PLA analysis (Figs. 4A, B and S10A). As shown in Fig. 4C–E and Fig. S10B–D, the protein level of Wls and its colocalization with SNX3 were increased in BLM-induced mice and *Snx3-cTg* mice. SNX3 deficiency inhibited the upregulation of Wls and the colocalization of Wls with SNX3 in mice (Figs. 4F, G and S10E–G). Ras-related protein 5a, an early endosomal marker protein, is involved in the budding and reverse transport of SNX3-retromer’s cargo protein from early endosome (EE) [50]. If the cargo protein is not recycled, it will enter LE and undergo protein degradation as the LE fuses with the lysosome, which is manifested as increased colocalization of it and the lysosomal marker Lysosomal-associated membrane protein 1 (LAMP1) [15]. When SNX3 was upregulated, the interaction of Wls with Rab5a increased significantly, suggesting that Wls were retrograded from EE budding for its intracellular recycling by SNX3 (Fig. 4H, Fig. S11A–G). After knocking down SNX3, the colocalization of Wls with Rab5a was decreased and the colocalization of Wls with LAMP1 was increased, implying that the intracellular recycling of Wls was inhibited when SNX3 was deficient (Fig. 4I and Fig. S12A–F). Similar results were also observed in FB cells (Fig. S13A–C). These results suggest that SNX3 interacted with Wls to promote the intracellular recycling of Wls and avoided its late endosomal-lysosomal degradation (Fig. 4J).

To examine the role of Wls in PF, adeno-associated virus serotype 6 (AAV6) -Wls or Vector were intratracheally delivered to *Snx3-cKO* mice. Two weeks later, a PF mouse model was established via intratracheal injections of BLM (Fig. 4K, L and Fig. S14A). The selective overexpression of AAV6-Wls in mice was validated (Fig. S14B, C). *Snx3-cKO* mice showed significantly ameliorated pulmonary dysfunction, reduced accumulation of collagen fibrils and elastic fibers, and decreased elevated levels of β-catenin that were caused by BLM. Interestingly, in AAV6-Wls-expressing mice, the protective effect of *Snx3-cKO* was partially reversed (Fig. S14D–G). Changes in protective effects and β-catenin of PF were also observed in AT2 cells (Figs. 4M, N and S14H–I). These results suggested that other important pathways involving SNX3 contribute to the progression of PF.

### CK-1α was identified as a novel cargo protein of SNX3 in pulmonary fibrosis

**Fig. 5:**
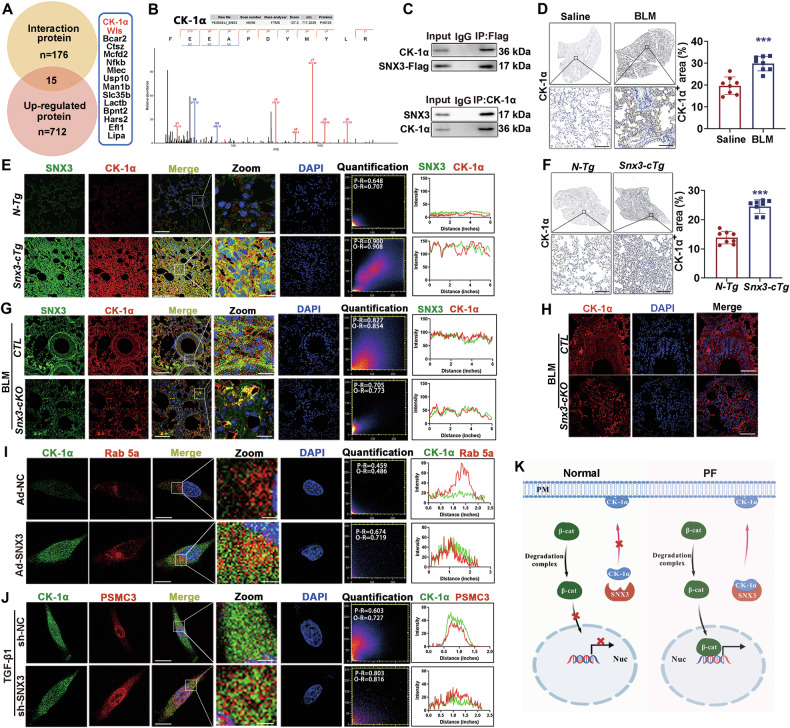
CK-1α was identified as a novel cargo protein of SNX3 in pulmonary fibrosis. **A** Ven diagram revealed the quantity of SNX3-interacting proteins overlapped by the IP-MS database and proteomics analysis. **B** The protein mass spectrum of CK-1α were shown. **C** Fresh lung homogenates were sparked by anti-Flag or anti-CK-1α for CK-1α or SNX3 detection in co-IP analysis. **D** The protein level of CK-1α in mice were detected by IHC staining analysis (Scale bar: 200 μm; *n* = 8 mice). **E** The change colocalization of SNX3 with CK-1α in *Snx3-cTg* mice were measured by IF staining analysis; Scale bar: 100 μm, *n* = 8 mice. **F** The protein level of CK-1α in *Snx3-cTg* mice were detected by IHC staining analysis (Scale bar: 200 μm; *n* = 8 mice). **G** The change colocalization of SNX3 with CK-1α in *Snx3-cKO* mice was measured by IF staining analysis; Scale bar: 100 μm, *n* = 8 mice. **H** The protein level of CK-1α in *Snx3-cKO* mice were detected by IF staining analysis (Scale bar: 100 μm, *n* = 8 mice). **I**-**J** IF staining analysis were performed to detect the protein levels and colocalization of CK-1α with Rab 5a and PSMC3; Scale bar: 10 μm; *n* = 3 experiments. **K** The flow chart of CK-1α intracellular recycling and degradation. Were created with BioGDP.com The data were shown as means ± SEM. *P < 0.05 vs. *CTL+ Saline* group *or* sh-NC. ^#^*P* < 0.05 vs *CTL* + *BLM* group *or* sh-NC + TGF-β1. ns, not significant.

As shown in the Fig. 4A–J, SNX3 interacted with Wls to promote intracellular recycling of Wls to activate Wnt/β-catenin pathway and promote fibrosis. Whether SNX3 has other key proteins involved in this pathway besides Wls to induce pulmonary fibrosis? Based on IP-MS results and 712 upregulated proteins in the BLM model analyzed by proteomic analysis, we identified 15 proteins (Fig. 5A). Among the 15 identified proteins, CK-1α attracted our interest because it is associated with the Wnt/β-catenin signaling pathway (Figs. 5A, B and S15A, B). CK-1α, another key member of the β-catenin protein degradation complex, is recruited by the scaffold protein Axin to phosphorylate β-catenin, which is required for further ubiquitination and proteasomal degradation of β-catenin [51]. We found that SNX3 interacted with CK-1α (Fig. 5C), and were curious about whether SNX3 has a regulatory effect on CK-1α and whether this effect was related to Wnt/β-catenin signaling pathway and PF.

The protein level of CK-1α and the colocalization with SNX3 were increased in BLM-induced mice and Snx3-cTg mice (Fig. 5D–F and Fig. S15C–E). *Snx3-cKO* mice inhibited the upregulation of CK-1α and the colocalization of CK-1α with SNX3 (Figs. 5G, H and S15F–K). The upregulation of SNX3 caused a significant increase in the colocalization of CK-1α with Rab5a, indicating that CK-1α was retrograded from EE budding by SNX3 for recycling (Fig. 5I and Fig. S16A–F). The proteasome 26S subunit (PSMC3) plays an important role in proteasomal degradation system [52]. The knockdown of SNX3 increased the colocalization of CK-1α and PSMC3, whereas the colocalization of CK-1α and Rab5a was downregulated, suggesting that the SNX3 knockdown inhibited the intracellular recycling of CK-1α, and most of CK-1α entered the proteasome degradation pathway (Figs. 5J, and S17A–F). Similar results were also observed in FB cells (Fig. S18A–C).

In AT2 cells, SNX3 overexpression induced β-catenin accumulation and α-SMA-associated fibrosis, partially attenuated by Wls knockdown (Fig. S19A–C). CK-1α increased with SNX3 overexpression but remained unchanged post-Wls knockdown (Fig. S19D), indicating SNX3-specific association. Complementarily, CK-1α overexpression induced fibrotic effects partially rescued by SNX3 knockdown (Fig. S19E, F) but failed to restore Wls expression (Fig. S19G). In Snx3-cKO mice, AAV6-CK-1α administration did not reverse BLM-induced Wls suppression (Fig. S19H), confirming CK-1α operates through SNX3 without influencing Wls-mediated Wnt secretion.

Above all, CK-1α was a new cargo protein of SNX3 and SNX3 directly interacted with CK-1α to regulate its intracellular recycling and degradation (Fig. 5K).

### SNX3/CK-1α axis induced Wnt/β-catenin signaling pathway activation and pulmonary fibrosis

**Fig. 6:**
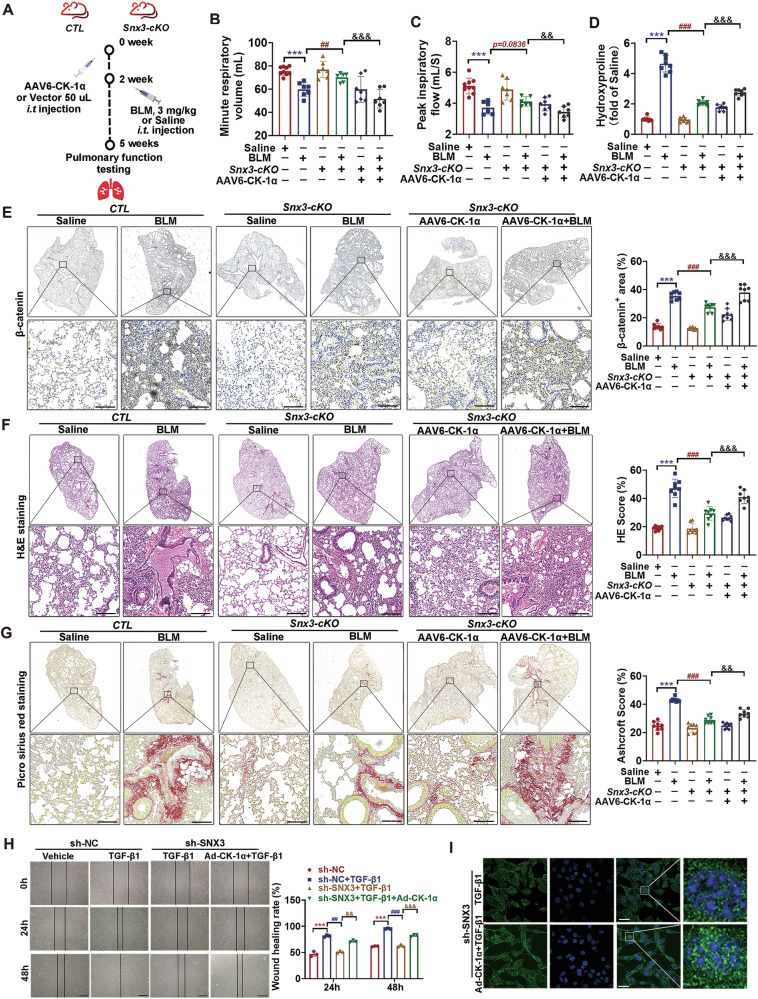
SNX3/CK-1α axis induced Wnt/β-catenin signaling pathway activation and pulmonary fibrosis. **A** *Snx3-cKO* mice and *CTL* mice were administered AAV6-CK-1α (50 μL, *i.p* injection) and BLM (3 mg/kg, intratracheal instillation). **B**, **C** Minute respiratory volume and Peak inspiratory flow were detected by the EMKA system; *n* = 8 mice. **D** Representative images of hydroxyproline concentration were shown, *n* = 8 mice. **E** Representative images of IHC staining analysis of β-catenin in lung tissue sections as indicated (Scale bar: 200 μm; *n* = 8 mice). **F**, **G** H&E staining and PSR staining of lung tissues are shown; Scale bar: 200 μm, *n* = 8 mice. **H** The wound healing analysis detected the migratory ability of AT2 cells; Scale bar: 200 μm; *n* = 3 experiments. **I** The protein level and nuclear distribution of β-catenin were measured by IF staining analysis (Scale bar: 25 μm, *n* = 3 experiments). The data were shown as means ± SEM. *P < 0.05 vs. *CTL+ Saline* group *or* sh-NC. ^#^*P* < 0.05 vs *CTL* + *BLM* group *or* sh-NC + TGF-β1. ^&^*P* < 0.05 vs *Snx3-cKO* + *BLM* group *or* sh-SNX3 + TGF-β1. ns, not significant.

β-catenin is phosphorylated by CK-1α, phosphorylated by GSK-3β, and then degraded by ubiquitination and proteasome [51]. To investigate whether the effect of SNX3 on the retrograde transport of CK-1α to the PM was involved in the Wnt/β-catenin signaling pathway and PF, we used *Snx3-cKO* mice and littermate control mice after intratracheal instillation of AAV6-CK-1α and BLM (Fig. 6A). As shown in Figs. 6B–G and S20A–C, the levels of β-catenin protein, pulmonary dysfunction, fibrosis areas, collagen fibrils, and hydroxyproline concentration were significantly reduced in *Snx3-cKO* mice compared with BLM mice, but AAV6-CK-1α reversed this lung-protective effect of SNX3 deficiency, as was also observed in AT2 cells (Figs. 6H–I and S20D–H). These results suggest that the transport of CK-1α to the PM by SNX3 may be a novel pathway for bypassing Wnts and regulating β-catenin in the PF process.

### A novel tetrahedron TDN-LC4 targeting SNX3 ameliorated pulmonary dysfunction and fibrosis

**Fig. 7:**
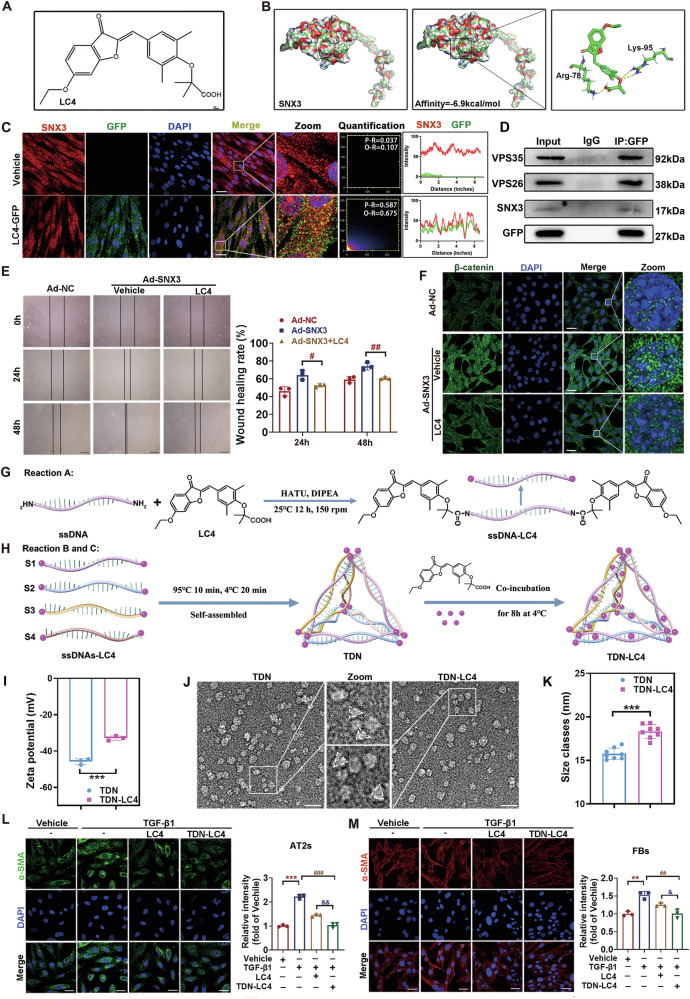
A novel tetrahedron TDN-LC4 targeting SNX3 ameliorated pulmonary dysfunction and fibrosis. **A** Chemical structure of LC4. **B** Diagram of 3D SNX3 structure, SNX3 binding with LC4 and magnification of SNX3 and LC4 binding. **C** IF staining analysis were performed to detect the protein level and colocalization of LC4-GFP with SNX3. Scale bar: 25 μm; *n* = 3 experiments. **D** LC4 treated FBs were sparked by anti-GFP detection in co-IP assays. **E** Representative images of wound healing analysis; Scale bar: 200 μm, *n* = 3 experiments. **F** The protein level of β-catenin was detected by IF staining analysis. Scale bar: 25 μm; *n* = 3 experiments. **G**, **H** Schematic synthesis process of TDN-LC4. **I** Zeta potentials of TDN and TDN-LC4. **J** TEM and images of TDN and TDN-LC4, Scale bar: 25 nm. **K** The diameters of the TDN and TDN-LC4. **L**-**M** IF staining analysis were performed to detect indicating that α-SMA positive cells in AT2s and FBs; Scale bar: 25 μm, *n* = 3 experiments. The data were shown as means ± SEM. *P < 0.05 vs. Ad-NC, TDN *or* Vehicle. ^#^*P* < 0.05 vs Ad-NC, TGF-β1 *or* TGF-β1 + LC4. ^&^*P* < 0.05 vs LC4 + TGF-β1 ns, not significant.

As mentioned above, we found that SNX3 contributes to PF by activating the Wnt/β-catenin signaling pathway. Therefore, our objective was to identify small molecule inhibitors targeting SNX3 to inhibit the Wnt/β-catenin pathway and prevent the progression of PF. To this end, we performed molecular docking of SNX3 with our library of >50,000 small molecule compounds to identify potential inhibitors. For small molecules with high docking scores, we performed co-immunoprecipitation (co-IP) and IF assays to further verify their interaction with SNX3 (Fig. 7A–D). The interaction between SNX3 and LC4 was predicted by molecular docking, and the 3D docking model revealed that the small molecule LC4 bound to the Arg-76 and Lys-95 residues of SNX3 (Fig. 7B). LC4 was coupled with GFP fluorescent labeling, and the interaction between SNX3 and GFP-LC4 was confirmed by co-IP assays (Fig. 7C, D). Taken together, these results demonstrate that LC4 targets SNX3 to exert biological effects. We explored the effects of LC4 on PF caused by SNX3 in vitro. As shown in Figs. 7D–F and S21A–H, the protein levels of SNX3, β-catenin, proliferation and migration were significantly decreased in AT2 cells after treatment with 10 μM LC4, implying that LC4 reversed the PF by decreasing SNX3 protein expression.

Tetrahedral framework nucleic acids (TDNs) nanostructures have become a prominent research target in natural materials for living organisms due to their excellent biocompatibility [53, 54]. In order to further enhance the medicinal efficacy and reduce the side effects of LC4, we designed a DNA tetrahedron to achieve an efficient antifibrotic effect of LC4 through a dual loading approach. The synthesis of the TDN-LC4 complex involved three steps, as detailed in Fig. 7G, H. We designed four partially complementary DNA sequences (ssDNA) (Table S12). To obtain the ssDNA-LC4 complex, LC4 and ssDNA were mixed with HATU and DIPEA, and the mixture was heated at 25 °C for 120 min before being gradually cooled to 4 °C (Fig. 7G). Through complementary base pairing, the four ssDNA-LC4 strands self-assembled to gradually form tetrahedra, termed TDN. Subsequently, LC4 was inserted into the double-stranded structure via π–π stacking, leading to the encapsulation of the TDN-LC4 complex (Fig. 7H). The formation of the DNA structure was characterized by agarose gel electrophoresis. As the number of paired DNA strands increased, the structure migrated more slowly during electrophoresis, exhibiting a single band (Fig. S22A). This indicated efficient formation of complementary structures. Furthermore, LC4 insertion into the double-stranded structure did not affect the migration of the TDN structure (Fig. S22B). Due to the encapsulation of LC4, the TDN-LC4 complex displayed characteristic UV absorption peaks at 260 nm (for DNA) and 380 nm (for LC4) (Fig. S22C). The ζ potential increased from −45.77 mV to −32.89 mV in the TDN-LC4 complex compared to TDN alone Fig. 7I. Furthermore, we observed the typical tetrahedral morphology of the TDN-LC4 complex using transmission electron microscopy (TEM), revealing that the molecular sizes of TDN and TDN-LC4 were ~15.8 nm and 18.4 nm, respectively (Fig. 7J, K). In summary, LC4 can be embedded in the helical structure of TDN through chemical bonding and π–π stacking interactions, suggesting the successful construction of a nanomedicine system, TDN-LC4.

To evaluate the antifibrotic capability of TDN-LC4, we first assessed its ability to enter AT2 and FBs cells, which is a crucial step for its subsequent biofunction. Cy5 fluorescence labeling allowed us to trace the TDN-LC4 complex, and its cellular uptake in the TGF-β1-stimulated fibrotic state was observed using immunofluorescence (IF) assays. As shown in Fig. S22D, E, the cellular uptake of both TDN and TDN-LC4 significantly increased between 6 and 12 h. Furthermore, TDN and TDN-LC4 exhibited progressive accumulation in lysosomes, evidenced by increasing co-localization with lysosomal marker LAMP1 from 6 to 12 h post-exposure suggesting ongoing lysosomal degradation. Concurrently, TDN and TDN-LC4 dissociation from early endosome marker Rab5 was observed by 6 h were able to successfully escape from endosomes, being released into the cytoplasm by 6 h, which is critical for the bioapplications of AT2 and FBs cells (Fig. S22E–I). Next, we assessed the stability of TDN-LC4 in cell lysis buffer at 37 °C, demonstrating that TDN-LC4 remained stable for at least 24 h (Fig. S22J, K). These results confirm that TDN-LC4 is an excellent biological agent.

The pathological process of idiopathic pulmonary fibrosis (IPF) is characterized by sustained fibroblast activation and epithelial-mesenchymal transition (EMT), both of which contribute to accumulation of myofibroblasts at the site of the injury, excessive extracellular matrix (ECM) deposition, and subsequent lung dysfunction [55, 56]. To mimic pulmonary fibrosis in vitro, we exposed AT2 and FBs cells to TGF-β1, thereby generating an in vitro PF model. As expected, we observed a significant reduction in the expression of fibrosis markers α-SMA and COL-1 after treatment with TDN-LC4 compared to LC4 alone. Furthermore, TDN-LC4 inhibited TGF-β1-induced cell migration and proliferation, and TDN-LC4 treatment effectively reduced ROS production (Figs. 7L, M and S23A–H). In summary, TDN-LC4 effectively enhances the inhibition of fibroblast activation and reduces cell proliferation and migration in vitro compared to LC4.

### TDN-LC4 targeting SNX3 ameliorated pulmonary dysfunction and fibrosis

**Fig. 8:**
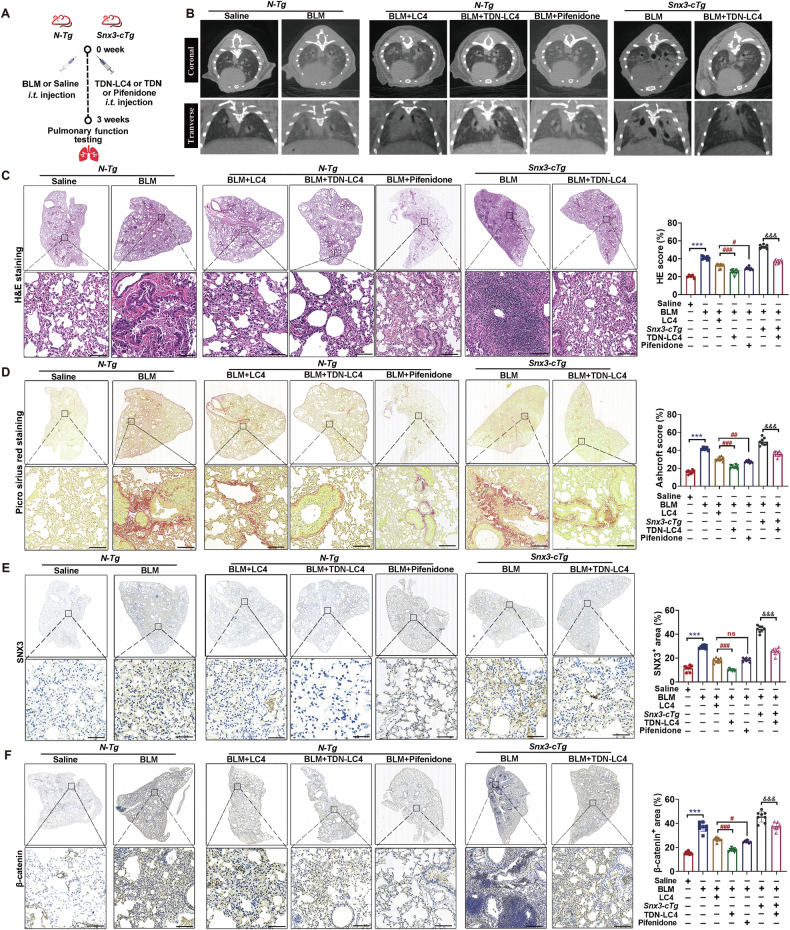
TDN-LC4 targeting SNX3 ameliorated pulmonary dysfunction and fibrosis. **A** *Snx3-cTg* mice and *N-Tg* mice were administered BLM and TDN-LC4 at the same time for 3 weeks, with pirfenidone (PFD) as a positive control treatment. **B** Micro-CT revealed the fibrotic area of the lung tissues; *n* = 6 mice. **C**, **D** HE staining and PSR staining analysis were shown; Scale bar: 200 μm, *n* = 8 mice. **E**, **F** Representative images of IHC staining analysis for SNX3 and β-catenin as indicated (Scale bar: 200 μm; *n* = 8 mice). The data were shown as means ± SEM. *P < 0.05 vs. *Saline*. ^#^*P* < 0.05 vs *BLM* + *LC4* group *or* sh-NC + TGF-β1. ^&^*P* < 0.05 vs *Snx3-cTg* + *BLM* group. ns, not significant.

To study the role of TDN-LC4 in SNX3-induced PF, *Snx3-cTg* mice and *N-Tg* mice were administered BLM followed by injections of either TDN-LC4 or pirfenidone injection for weeks (Fig. 8A). As expected, BLM-treated mice remarkably aggravated the pulmonary functional injury, fibrosis areas, β-catenin protein levels and hydroxyproline concentration. Pirfenidone, LC4, and TDN-LC4 demonstrated varying degrees of efficacy in mitigating these alterations. Comparative analysis revealed that LC4 achieved therapeutic efficacy equivalent to pirfenidone in inhibiting BLM-induced PF, while TDN-LC4 exhibited a significantly superior antifibrotic effect (Figs. 8B–F and S24A–F). Consistent with Fig. 3, Snx3-cTg mice developed pronounced lung injury. TDN-LC4 reversed this fibrotic effect of SNX3 overexpression, suggesting that the administration of TDN-LC4 abolished the fibrotic effect of overexpressed SNX3 in mice (Figs. 8B–F and S24A–F). These results provided evidence that TDN-LC4 targeting SNX3 effectively ameliorated Wnt/β-catenin pathway activation, pulmonary dysfunction and fibrosis.

Additionally, to study whether TDN-LC4, LC4, or pirfenidone caused organ toxicity while achieving therapeutic effects, we assessed the safety of the drugs to the principal organs by a series of examination. The morphology of the heart, liver, spleen, and kidneys was analyzed using H&E staining. As shown in Fig. S25A–D, all treatment groups (TDN-LC4, LC4, and pirfenidone) showed no significant structural damage in overall and localized tissue views.

Examination of fibrosis, inflammation, and Wnt signaling pathway activation in lung tissue demonstrated that both LC4 and TDN-LC4 alleviated these parameters to varying degrees (Fig. S26A–L). The intratracheal administration of BLM did not induce fibrosis, inflammation, or Wnt signaling activation in the liver or kidneys, and neither LC4 nor TDN-LC4 produced significant changes in these organs (Figs. S27A–L, S28A–L). These results collectively confirm the safety of therapeutic doses of LC4 and TDN-LC4 in vivo.

To evaluate the safety profile of high-dose TDN-LC4 in healthy mice, we conducted a 1 month toxicity assessment following administration at 10-fold the therapeutic dose (Fig. S29A). Results demonstrated no significant mortality (Fig. S29B) and negligible body weight reduction (Fig. S29C). Pulmonary function parameters showed no impairment (Fig. S27D–F), and serum markers of hepatic function (Fig. S29G–I) and renal function (Fig. S29J–L) remained within normal physiological ranges. Histopathological analysis revealed no structural abnormalities in major organs (Fig. S29M). Concomitant evaluation of inflammation, fibrosis, and Wnt pathway activation in the lungs, liver, and kidneys showed no significant alterations (Fig. S30A–C). Although limited by truncated chronic toxicity data, these findings indicate an absence of overt toxicity under these experimental conditions.

### Biodistribution and pharmacokinetics assessment of TDN-LC4 in healthy and fibrotic mice

**Fig. 9:**
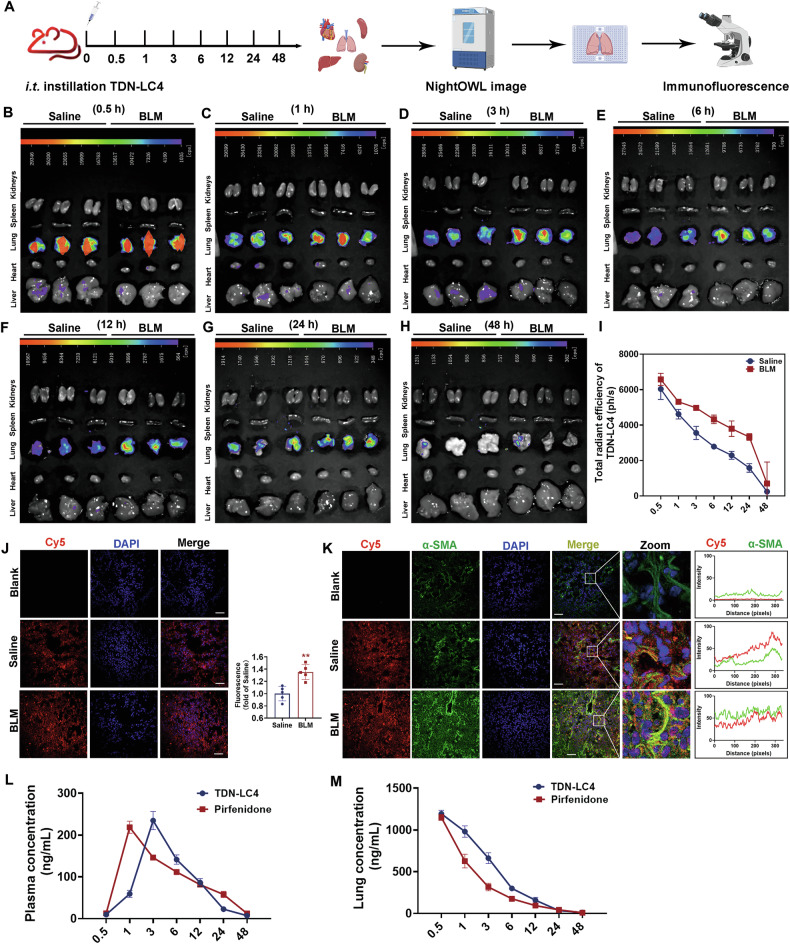
Biodistribution assessment of TDN-LC4 in healthy and BLM-induced pulmonary fibrosis mice. **A** Experimental schema illustrating ex vivo fluorescence imaging and immunofluorescence staining for Cy5-TDN-LC4 biodistribution. **B**–**H** Representative ex vivo fluorescence images of major organs (heart, liver, lungs, spleen, kidneys) at serial timepoints (0.5, 3, 6, 12, 24, 48 h) post-intratracheal instillation of Cy5-TDN-LC4. **I** Quantitative analysis of pulmonary fluorescence intensity across timepoints (*n* = 6 mice). **J** Confocal microscopy of Cy5-TDN-LC4 biodistribution in lung sections from Saline or BLM groups (24 h). Scale bars: 100 μm. **K** Immunofluorescence co-localization of Cy5-TDN-LC4 (red) with α-SMA (green) in normal or fibrotic lungs. Nuclei counterstained with DAPI (blue). Scale bars: 100 μm. *n* = 6 mice. Data were shown as means ± SEM. **L** Lung tissue concentration-time profiles of TDN-LC4 (blue) and pirfenidone (red) measured. **M** Corresponding plasma concentration-time curves of TDN-LC4 (blue) and pirfenidone (red) measured. Error bars represent SEM (*n* = 6 mice). *P < 0.05 *vs*. Saline. ns, not significant.

To evaluate pulmonary targeting of TDN-LC4, we performed fluorescence tracking using Cy5 labeling and pharmacokinetic analysis. Cy5-TDN-LC4 was administered via intratracheal instillation to mice treated with BLM or saline. Mice were euthanized at 0.5,1, 3, 6, 12, 24, and 48 h post-administration, and major organs were harvested for ex vivo fluorescence imaging and immunofluorescence staining (Fig. 9A). Analysis demonstrated significantly stronger Cy5-TDN-LC4 fluorescence signals in the lungs compared to other organs (Fig. 9B–H). Critically, in fibrotic lungs, Cy5-TDN-LC4 fluorescence persisted for over 24 h (Fig. 9I). Immunofluorescence of lung sections confirmed distinct Cy5 signals at 12 h of administration (Fig. 9J, K). Enhanced colocalization of Cy5 with α-SMA-positive myofibroblasts in BLM-treated mice suggested prolonged retention in these cells (Fig. 9L, M). Pharmacokinetic profiling was performed (Fig. 9N, O, Tables S13–S15). In lung tissue, TDN-LC4 demonstrated significantly higher exposure (AUC_0-48h_ = 6223.02 ± 500 ng·h/mL) and an extended half-life (*t*_1/2_ = 13.40 h) compared to pirfenidone. Systemic exposure was similar, though TDN-LC4 exhibited a delayed plasma T_max_ (3.0 h *vs*. 1.0 h). Notably, TDN-LC4 achieved a higher lung-to-plasma ratio (2.147 ± 0.231) than pirfenidone (1.625 ± 0.086). Minimal fluorescence signal from Cy5-labeled TDN-LC4 was detected in kidney tissue (Fig. S31A), while hepatic signal was largely eliminated by 6 h (Fig. S31B). These results support TDN-LC4’s pulmonary targeting.

## Discussion

Wnt/β-catenin signaling is a well-recognized therapeutic target in the pathogenesis of PF [57]. However, interventions targeting a single target of this pathway have not achieved satisfactory efficacy [21]. This study reveals that SNX3 promotes PF through dual mechanisms: (1) recycling Wls to transport Wnts to extracellular and activate Wnt-dependent pathways, and (2) bypassing Wnts and directly translocating CK-1α to the PM to destroy the β-catenin degradation complex, thereby exerting dual activation of β-catenin and PF. By targeting SNX3 to screen small molecule inhibitors, LC4 was found to have excellent effects on inhibiting Wnt/β-catenin signaling and treating PF.

SNX3, a member of SNXs family, plays important roles in various physiological and pathological processes by mediating the intracellular trafficking of cargo proteins from EE to the TGN, PM or nucleus [9, 58–60]. For example, our laboratory reported that SNX3 induces heart diseases by reversing the transport of its cargo proteins to the PM or nucleus [9, 10]. Unlike other SNX subtypes, SNX3 has the simplest structure and contains a highly conserved PX domain [50]. The crystal structure of SNX3-retromer complex formed by SNX3 and VPS proteins is clear, making it easier to target for drug design [15]. The advantages of SNX3 encouraged us to further investigate its role in different diseases and regulatory mechanisms. This study confirms the importance of SNX3 in PF by activating Wnt/β-catenin signaling pathway.

Wnt signaling pathway is still worthy of in-depth study, despite being explored for many years by us [19, 20] and others [18, 61, 62]. This pathway plays a key role in fibrosis in different tissues and has not been fully elucidated due to its great complexity [57]. The complexity may be attributed to the possible redundancy of signal molecules in this pathway, and there are non-canonical Wnt/PCP and Wnt/Ca^2+^ pathways, in addition to the canonical Wnt/β-catenin pathway [63]. The multiple crosstalk between Wnt signaling and other signaling pathways (such as TGFβ1/Smads pathways [8]) is also one of the important reasons for the complexity of the signaling network. This paper focuses on the intracellular transport patterns of Wls and CK-1α, two key molecules of Wnt signaling pathway, in the process of PF.

In Wnt signaling pathway, Wls is known to carry the secretion of lipid-modified Wnt proteins to initiate this signaling pathway [14, 64, 65]. In Drosophila, SNX3 was found to rescue Wls from the lysosomal degradation pathway and to recycle it in the cell to promote Wnt secretion [51]. In a mouse model of PF, we similarly found that SNX3 deletion led to Wls degradation via the late endosomal-lysosomal pathway, and the reduction of Wls prevented Wnt secretion and thus PF. Once Wnts bind to their core components, the scaffold protein Axin translocates to the PM, and subsequently recruits CK-1α and GSK-3β to the PM; subsequently, β-catenin increases, and the signal is activated. However, when SNX3 deletion decreased Wls recycling, AAV6-Wls still partially reduced β-catenin degradation and promoted its translocation into the nucleus, suggesting that there are other key cargo proteins that plays an important role in inducing β-catenin and PF.

We searched for the possible reasons why SNX3 inhibited β-catenin degradation. CK-1α, one of the key components of the β-catenin destruction complex, was screened and identified as another cargo protein of SNX3 by IP-MS and proteomic experiments. CK-1α phosphorylates β-catenin at Ser45 to prepare it for subsequent phosphorylation by GSK-3β, ubiquitination and proteasomal degradation by the β-TrCP-containing E3 ubiquitin protein ligase [66]. We found that SNX3 interacted with and retrogradely recycled CK-1α from early endosomal budding to the PM rather than being degraded by the proteasome pathway. AAV6-CK-1α almost abolished the protective effect of *Snx3-cKO* on PF, suggesting that the role of SNX3/CK-1α axis in activating β-catenin was also attributed to inhibiting its protein degradation.

In other words, another mechanism by which SNX3 activated the Wnt/β-catenin signaling pathway involved its direct transport of CK-1α to the PM, which led to the destruction of the degradation complex and the inhibition of β-catenin degradation. This effect of SNX3 was very similar to the recruitment effect of the scaffold protein Axin on CK-1α. Whether SNX3 also has a scaffold role, in addition to being a transporter, needs to be further clarified in the future. Of course, in addition to Wls and CK-1α, there remain other potential cargo proteins of SNX3 that play a role in PF or other organ fibrosis through certain pathways less related to the Wnt/β-catenin signaling pathway, which is also the subject of our future research. Our proteomic and IP-MS analysis revealed several SNX3 interacting proteins, which may shed more light on the role of SNX3 in PF. In addition, SNX3-driven activation of Wnt/β-catenin may also cause extensive organ fibrosis, such as cardiac fibrosis and liver fibrosis, which needs to be confirmed by further research.

Our experimental data support that SNX3 may play an important regulatory role in two key nodes of the Wnt/β-catenin signaling pathway. In fact, the Wnt/β-catenin signaling pathway involves multiple redundant or compensatory signaling molecules, such as sFRP1 and DKK1, which were discovered earlier by our laboratory [19, 20]. Is it more advantageous to target SNX3 for simultaneous regulation of Wls and CK-1α? To this end, we screened inhibitors targeting SNX3 and identified a novel small molecule LC4, which effectively ameliorated pulmonary dysfunction and reversed pulmonary fibrosis. Additionally, we developed TDN-LC4, a DNA tetrahedron-based nanomedicine, as a versatile platform to deliver the small molecule LC4. Taking advantage of the high structural stability and programmability of DNA tetrahedra, TDN-LC4 aims to improve the targeted delivery and bioavailability of LC4 to diseased lung tissue, overcoming some of the limitations seen with conventional drug delivery systems, such as nonspecific biodistribution and poor cellular uptake [67, 68]. However, challenges remain to optimize the delivery of DNA tetrahedra to the lungs and ensure adequate accumulation at the target site [38, 67]. Inefficient tissue targeting could limit the effectiveness of TDN-LC4 in treating PF. Therefore, improving the tissue-specific delivery and uptake of these nanostructures is a key area for future research.

In conclusion, this study identifies SNX3 as a key regulator of PF through dual Wnt/β-catenin activation via Wls recycling and CK-1α trafficking. SNX3 upregulation in AT2 cells drives fibrotic progression, while its knockout or pharmacological inhibition by the novel TDN-LC4 nanodrug attenuates Wnt/β-catenin signaling, fibrosis, and lung dysfunction, offering a multi-target therapeutic strategy for PF.

## Supplementary information

Supplemental tables and figures

Related Manuscript File

aj-checklist

## Data Availability

All data needed to evaluate the conclusions in the paper are present in the paper and the supplementary information.
