# Supplementary material for: Targeting sorting nexin 3 to treat pulmonary fibrosis by dual modulating Wnt/β-catenin signaling
Source: Cell Death Dis. 2026 Jan 15;17(1):43. doi: 10.1038/s41419-025-08248-x (PMC12808692; doi:10.1038/s41419-025-08248-x)
Supplement: Supplementary file 1 — Supplemental tables and figures [file 41419_2025_8248_MOESM1_ESM.pdf]

Supplementary information for

Targeting sorting nexin 3 to treat pulmonary fibrosis by dual modulating Wnt/β-catenin signaling

Dinghu Ma, Wenjing Yu, Hang Zhou, Rongyuan Lin, Ximeng Sun, Mingxia Peng, Chenjia Lin, Haoyu Du, Yueqi Li, Huimin Liang, Duanping Sun, Peiqing Liu, and Jing Lu

This Word file includes:

Table S1-S15 and Figure S1-S31

Tables:

Table S1 Clinical characteristics of patients in GSE76808 dataset

Accession	Title	Source name	Tissue	Patient
GSM2038267	RLB6SD31	Human lung biopsy	lung	patient31and08
GSM2038274	RLA9SD08	Human lung biopsy	lung	patient31and08
GSM2038269	RLB4SD26	Human lung biopsy	lung	patient26
GSM2038272	RLB1SD17	Human lung biopsy	lung	patient17
GSM2038271	RLB2SD22	Human lung biopsy	lung	patient13and22
GSM2038275	RLA11SD13	Human lung biopsy	lung	patient13and22
GSM2038268	RLB5SD28	Human lung biopsy	lung	patient07and28
GSM2038277	RLA8SD07	Human lung biopsy	lung	patient07and28
GSM2038276	RLA10SD11	Human lung biopsy	lung	patient06and11
GSM2038278	RLA7SD06	Human lung biopsy	lung	patient06and11
GSM2038273	RLA12SD14	Human lung biopsy	lung	patient05and14
GSM2038279	RLA6SD05	Human lung biopsy	lung	patient05and14
GSM2038270	RLB3SD23	Human lung biopsy	lung	patient01and23
GSM2038280	RLA5SD01	Human lung biopsy	lung	patient01and23
GSM2038281	RLA4NL16	Human lung biopsy	lung	control16
GSM2038282	RLA3NL13	Human lung biopsy	lung	control13
GSM2038284	RLA1NL09	Human lung biopsy	lung	control09

Table S2 The gRNA sequence of *Snx3* for CRSPR/Cas9

gRNAs	Sequence (5'-3')
gRNA1	5'-GTAAAATGGCTGTCCTTGTGAGG-3'
gRNA2	5'-GCTGGAAGTGGCTAAAAGGAAGG-3'

Table S3 The gRNA sequence of *Sftpc* for CRSPR/Cas9

gRNAs	Sequence (5'-3')
gRNA1	5'- AGCTGCTCCTGCCCAGAAAC CGG -3'

Abbreviations: *Sftpc*, C57BL/6Smoc-Sftpc $^{em(IRES\text{-}CreERT2)Smoc}$

Table S4 The primer sequences were applied to *Snx3-floxed* mice to genotype the correct 5' and 3' homology arm recombination

Primer	Sequences
P1	5'-TCTCCATCCTTGTCAACCGTGTAT-3'
P2	5'-ATTTGGGAGCCTGTTTCTTCATCT-3'
P3	5'-GGCTGGCCTTGAGTTCTTGAT-3'
P4	5'-CAGCTGTGCCCTTTACTTACTTGA-3'

Table S5 The primer sequences were applied to *Sftpc-cre* mice to genotype 5' and 3' homology arm recombination

Primer	Sequences
P1	5'-CAACGCCAAAGCAAAGAGTGAAAC-3'
P2	5'- TGACCAGAGTCATCCTTAGCG -3'
P3	5'- CAACGCCAAAGCAAAGAGTGAAAC -3'
P4	5'- ATCCACAGGGCCTAGCATCAGTCA -3'

Abbreviations: *Sftpc*, C57BL/6Smoc-Sftpc $^{em(IRES\text{-}CreERT2)Smoc}$

Table S6 The primer sequences were applied to genotype *Snx3*-flox and the activity of *Sftpc-Cre*

Genotype	Primer	Sequences
*Snx*3-flox	P5 (Forward)	5'-CTTTTAGAGGAGACGATGGAATA-3'
	P6 (Reverse)	5'-AGAAAGGCTGGAAGTGGCTAAA-3'
Sftpc-Cre	P7 (Common)	5'- ACACCGGCCTTATTCCAAG -3'
	P8 (Mutant)	5'- TGCTTCACAGGGTCGGTAG -3'
	P9 (Wild type)	5'- CATTACCTGGGGTAGGACCA -3'

Abbreviations: *Snx3*, Sorting nexin 3; *Sftpc*, C57BL/6Smoc-Sftpc $^{em(IRES\text{-}CreERT2)Smo}$

Table S7 The F1 generation genotype test results of *Snx3-cKO* mice

mice ID	DOB	generations	Sex	Snx3-eCKO1	Sftpc-cre
51	2021/8/27	F1	♂	HE	HE
52	2021/8/27	F1	♂	HE	WT
53	2021/8/27	F1	♂	HE	HE
54	2021/8/27	F1	♂	HE	HE
55	2021/8/27	F1	♂	HE	HE
56	2021/8/27	F1	♀	HE	HE

Abbreviations: *Snx3-cKO,* lung-specific Snx3 knockout mouse; *Sftpc*, C57BL/6Smoc-Sftpc $^{\text{em(IRES-CreERT2)Smoc}}$

Table S8 The primer sequences were applied to genotype *Snx3-cTg* mice

**Primer**	**Sequences**
Forward	5'- GCGTTGGCTACCCGTGATATT -3'
Reverse	5'- CACGACATTCAACAGACCTT -3'
Control Forward	5'- AGTGGCCTCTTCCAGAAATG-3'
Control Reverse	5'- TGCGACTGTGTCTGATTTCC-3'

Abbreviations: *Snx3-cTg,* lung-specific Snx3 transgenic mouse.

Table S9 The F1 generation genotype test results of *Snx3-cTg* mice

mice ID	DOB	generations	Sex	SNX3	sftpc-cre
A38	2022/7/30	F1	♂	-	WT
A39	2022/7/30	F1	♂	-	HE
A40	2022/7/30	F1	♂	-	HE
A41	2022/7/30	F1	♀	-	HE
A42	2022/7/30	F1	♀	-	WT
A43	2022/7/30	F1	♀	-	HE
A44	2022/8/2	F1	♀	-	HE
A45	2022/8/2	F1	♀	+	WT
A46	2022/8/2	F1	♂	-	HE
A47	2022/8/2	F1	♂	+	HE
A48	2022/8/2	F1	♂	+	HE
A49	2022/8/2	F1	♂	-	HE
A50	2022/8/2	F1	♂	+	WT
A51	2022/8/2	F1	♂	-	HE
A52	2022/8/2	F1	♀	-	HE
A53	2022/8/2	F1	♀	-	HE
A54	2022/8/2	F1	♀	-	WT
A55	2022/8/10	F1	♂	-	HE
A56	2022/8/10	F1	♀	-	WT

Abbreviations: *Snx3-cTg,* lung-specific Snx3 transgenic mouse.

Table S10 The siRNA for mice Wls gene

Primer	Sequences
siRNA-1	Forward: 5'- CGGCGUCACAGUCCAAGUGAATT-3'
	Reverse: 5'- UUCACUUGGACUGUGACGCCGTT-3'
siRNA-2	Forward: 5'- CACGAAUCCCUUCUACAGUAUTT -3'
	Reverse: 5'- AUACUGUAGAAGGGAUUCGUGTT-3'
siRNA-3	Forward: 5'- CUUCAUCAUCGUGGCUGGAAUTT-3'
	Reverse: 5'- AUUCCAGCCACGAUGAUGAAGTT-3'

Table S11 The sequences of real-time polymerase chain reaction (qPCR)

Primer	Sequences
TNF-α	Forward: 5'- CACCACGCTCTTCTGTCTCTACTGAAC-3'
	Reverse: 5'- AGATGATCTGAGTGTGAGGGTCTGG-3'
IL-1β	Forward: 5'- AAATACCTGTGGCCTTGGGC -3'
	Reverse: 5'- CTTGGGATCCACACTCTCCAG-3'
IL-6	Forward: 5'- ACTTCCAGCCAGTTGCCTTCTTG-3'
	Reverse: 5'- TGGTCTGTTGTGGGTGGTATCCTC-3'
IFN -γ	Forward: 5'- ATGGCTACACACTGCATCTTGG-3'
	Reverse: 5'- TGAGGCACAGTCATTGAATGCT-3
TGF-β1	Forward: 5'- CCTGCTGACCTTGCTGACTT -3'
	Reverse: 5'- GCTGCATCTTGGCTTGTTCT-3'

Fibronectin	Forward: 5'- TGCTGAAGCTGAAACAAGGC -3'<br>Reverse: 5'- CAGGTCTTGGGATGAGGTCG-3'
Collagen I	Forward: 5'- GCAGGAGAAGACCAAGACCG -3'<br>Reverse: 5'- GGTGGTGGTGAAGGTGAAGC-3'
α-SMA	Forward: 5'- CATCCAGGCATCGTCAACCA -3'<br>Reverse: 5'- GCTGATCCACGTCAGCAGTC-3'
Wls	Forward: 5'- TGAAGCTGAATCGGCTGAGA -3'<br>Reverse: 5'- CAGGTCTTCTGGTGATGGCA-3'
CK-1α	Forward: 5'- GGAGCAACAAGCAAGAAGGC -3'<br>Reverse: 5'- TCCTTGGAAGCTGGATGTCC-3'
β-catenin	Forward: 5'- TGAGCTGCTTCTGGATGTGC -3'<br>Reverse: 5'- GCTGGATCTCTCGTCTTCCA-3'

Table S12 The sequences of ssDNAs

ssDNAs	Base sequence (5'-3')
S1	NH2TATCACCAGGCAGTTGACAGTGTAGCAAGCTGTAATAGATGCGAGGG TCCAATACNH2
S2	NH2TCAACTGCCTGGTGATAAAACGACACTACGTGGGAATCTACTATGGC GGCTCTTCNH2
S3	NH2TTCAGACTTAGGAATGTGCTTCCCACGTAGTGTCGTTTGTATTGGACC CTCGCATNH2
S4	NH2ACATTCCTAAGTCTGAAACATTACAGCTTGCTACACGAGAAGAGCCG CCATAGTANH2
Cy5-S1	Cy5TATCACCAGGCAGTTGACAGTGTAGCAAGCTGTAATAGATGCGAGGG TCCAATACNH2

Table S13 Comparative pharmacokinetics parameters of TDN-LC4 and pirfenidone in lung tissue

	TDN-LC4	Pifenidone
$AUC_{0\text{-}48h}$	6223.02 ± 500 ng.h/ml	4841.26 ± 119.13 ng.h/ml
C_{max}	1193.33 ± 43.30 ng/mL	1150.5 ± 34.34 ng/mL
T_{max}	0.5 ± 0.0 h	0.5 ± 0.0 h
$t_{1/2}$	13.40 h	10.66 h

Table S14 Comparative pharmacokinetics parameters of TDN-LC4 and pirfenidone in plasma

	TDN-LC4	Pifenidone
$AUC_{0\text{-}48h}$	2898.1 ± 208.3 ng.h/ml	2979.8 ± 138.5 ng.h/ml
C_{max}	234.8 ± 19.6 ng/mL	218.7 ± 14.3 ng/mL
T_{max}	3.0 ± 0.0 h	1.0 ± 0.0 h
$t_{1/2}$	8.45 h	8.9 h

Table S15 Comparative the ratio of drug concentration in lung tissue to that in plasma (T/P)

	TDN-LC4	Pifenidone
T/P	2.147 ± 0.231	1.625 ± 0.086

Supplemental figures:

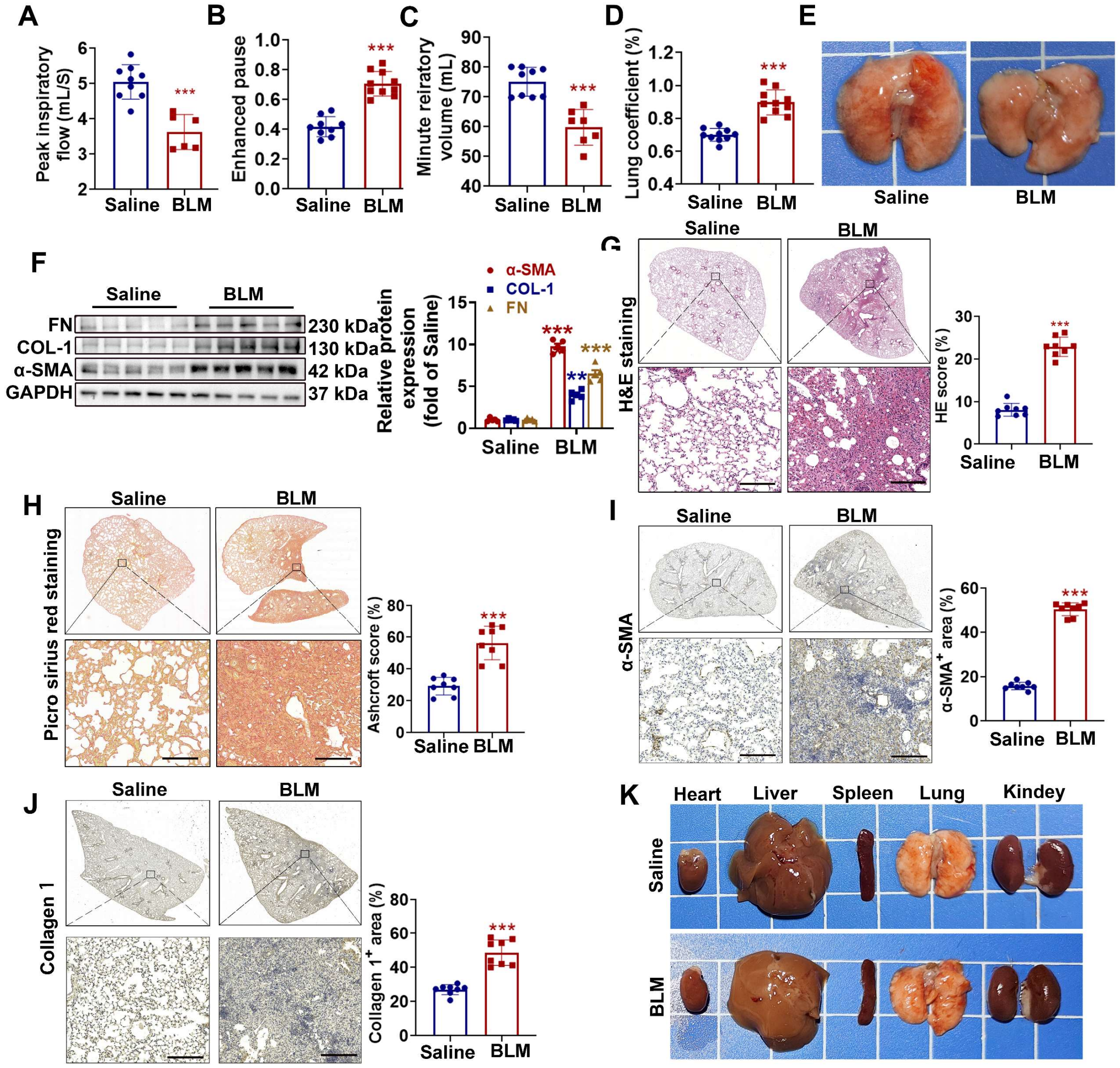

Figure S1 SNX3 expression was up-regulated in pulmonary fibrosis *in vivo*, related to Figure 1.

(A-C), Peak inspiratory flow, Enhanced pause and Minute respiratory reduced in BLM-induced mice were measured by EMKA system; *n*=8 mice. **(D)**, Lung coefficient was significantly increased compared to saline group; *n*=8 mice. **(E)**, Gross observation of the lungs. **(F)**, Representative images of western blotting analysis showing the protein level of FN, COL-1 and α-SMA; *n*=8 mice. **(G-H)**, H&E staining and PSR staining in lung tissue sections were shown. Scale bar: 200 μm, *n*=8 mice. **(I-J)**, Representative images of IHC staining analysis were shown; Scale bar: 200 μm, *n*=8 mice. **(K)**, Gross observation of lung showed significantly injury in BLM-induced mice expect for other organs. The data were shown as means ± SEM. *$P < 0.05$

vs. Saline group. ns, not significant.

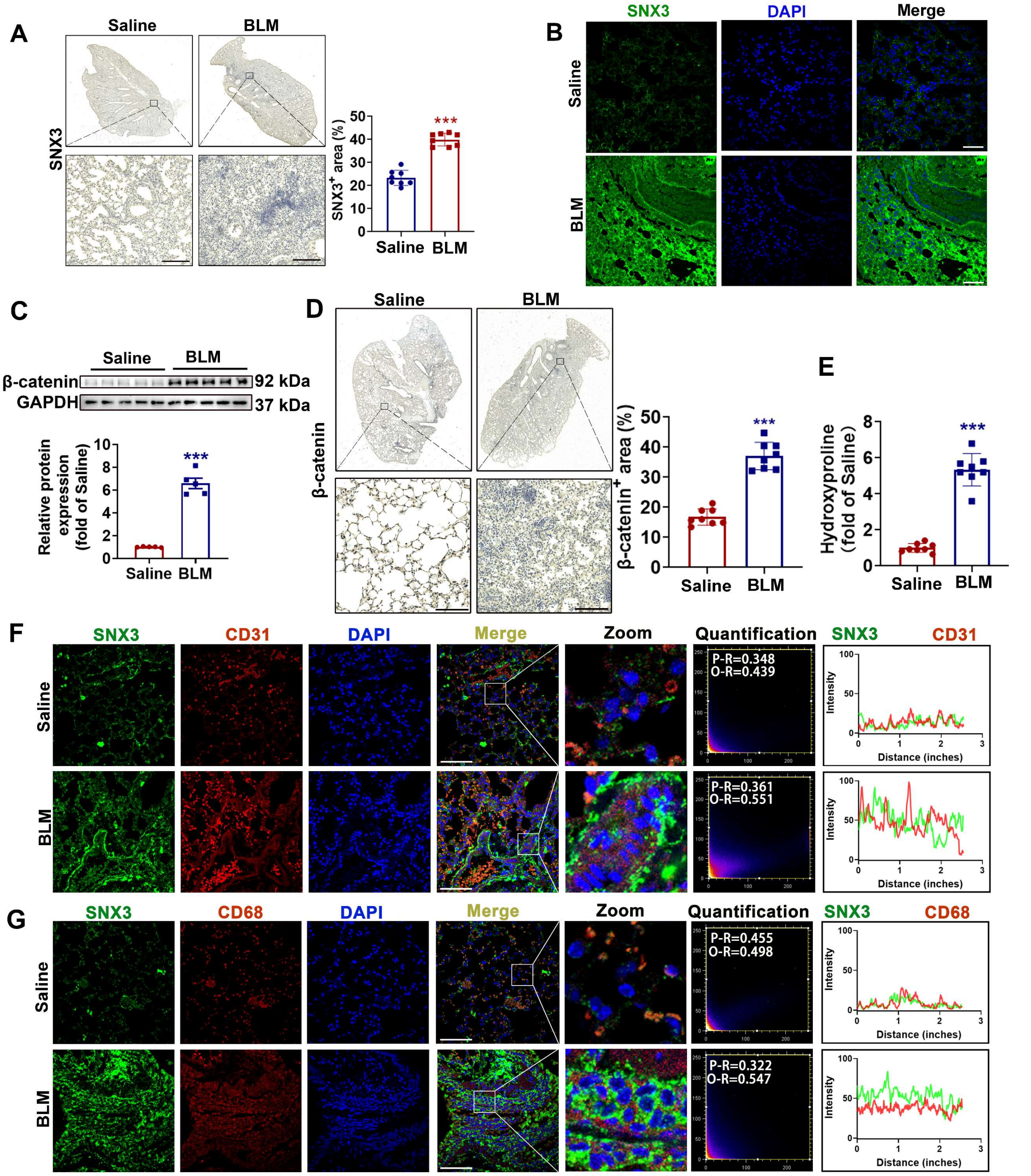

Figure S2 SNX3 expression was up-regulated in pulmonary fibrosis *in vivo*, related to Figure 1.

(A-B), Representative images of IHC staining analysis (Scale bar: 200 μm, *n*=8 mice) and IF staining analysis detected the protein level of SNX3 (Scale bar: 100 μm, *n*=8 mice). **(C-D)**, Representative images of western blotting and IHC staining analysis of β-catenin (Scale bar: 200 μm, *n*=8 mice). **(E)**, Representative images of hydroxyproline concentration were shown,

n=8 mice. **(F-G)**, The change colocalization of SNX3 with CD31 and CD68 in mice was measured by IF staining analysis; Scale bar: 100 μm, *n*=8 mice. The data were shown as means ± SEM. **P* < 0.05 vs. Saline group. ns, not significant.

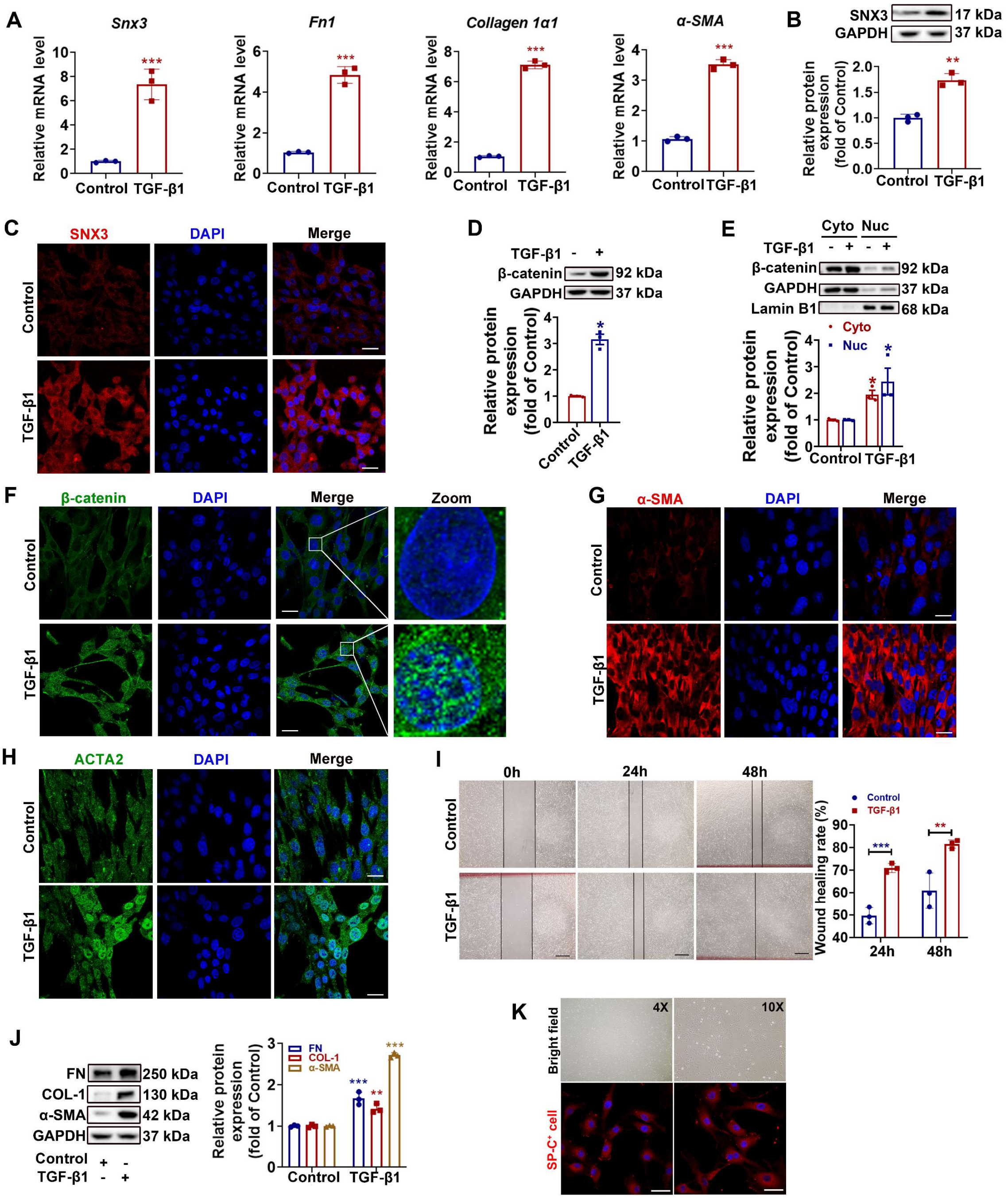

Figure S3 SNX3 expression was up-regulated in pulmonary fibrosis in AT2 cells, related to Figure 1.

(A), The relative mRNA level of *Fn1, Collagen 1a1, α-SMA* and *Snx3* were determined by qPCR; *n*=3 experiments. **(B)**, Western blotting analysis was used to detect the protein level SNX3, *n*=3 experiments. **(C)**, IF staining analysis indicated that SNX3 were augmented; Scale bar: 25 μm, *n*=3 experiments. **(D-F)**, The protein level and nuclear distribution of β-catenin were measured by western blotting analysis (*n*=3 experiments) and IF staining analysis (Scale

bar: 25 μm, *n*=3 experiments). **(G-H)**, IF staining analysis indicated that α-SMA and ACTA2 positive cells were augmented; Scale bar: 25 μm, *n*=3 experiments. **(I)**, The wound healing assay detected the migratory ability; Scale bar: 200 μm; *n*=3 experiments. **(J)**, Western blotting analysis was used to detect the protein level FN, COL-1 and α-SMA, *n*=3 experiments. **(K)**, Bright field and immunofluorescence analysis demonstrating type II alveolar epithelial cells (AT2), SP-C positive cells (red). Nuclei counterstained with DAPI (blue). Scale bar: 20 μm. The data were shown as means ± SEM. *$P < 0.05$ vs. Control group. ns, not significant.

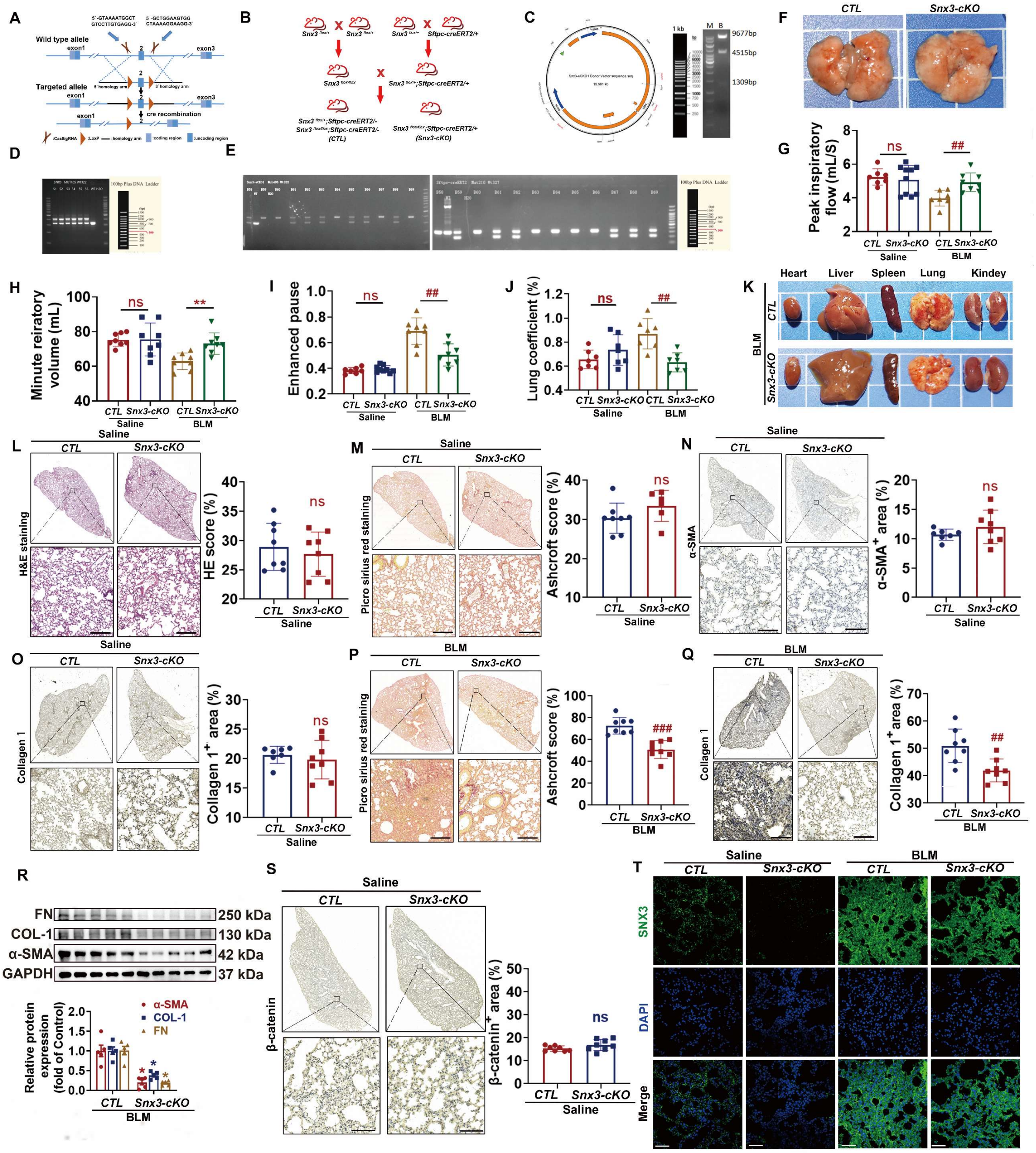

Figure S4 SNX3 deficiency alleviated pulmonary fibrosis in AT2 cells, related to Figure 2. **(A-B)**, The scheme of *Snx3-cKO* mice construction strategy and breeding process was shown. **(C)**, The plasmid profiles of donor vector were presented. **(D)**, The plasmid vector was detected by the agar gel electrophoresis. **(E)**, The genotypes were shown. Marker: 100 bp plus DNA ladder. **(F)**, Gross observation of lung was shown. **(G-I)**, Peak inspiratory flow, Enhanced paused and Minute respiratory were measured by EMKA system; *n*=8 mice. **(J)**, The lung coefficient ratio was calculated; *n*=8 mice. **(K)**, Gross observation of organs was shown. **(L-M)**, H&E staining and PSR staining indicated that there is no obvious difference between *Snx3-*

cKO group and *CTL* group; Scale bar: 200 μm, *n*=8 mice. **(N-O)**, Representative images of IHC staining analysis in *Snx3-cKO* mice were shown; Scale bar: 200 μm, *n*=8 mice. **(P)**, PSR staining of BLM-induced *Snx3-cKO* mice were shown; Scale bar: 200 μm, *n*=8 mice. **(Q),** Representative images of IHC staining analysis in *Snx3-cKO* mice were shown; Scale bar: 200 μm, *n*=8 mice. **(R),** Representative images of western blotting analysis indicting that the protein levels of FN, α-SMA and and COL-1 were shown, *n*=8 mice. **(S)**, Representative images and quantification of β-catenin; Scale bar: 200 μm, *n*=8 mice. **(T)**, *Snx3-cKO* mice was detected by IF staining analysis; Scale bar: 100 μm, *n* = 8 mice. The data were shown as means ± SEM. *$P < 0.05$ vs. *CTL+ Saline group*. $^{\#}P < 0.05$ vs *CTL+ BLM group*. ns, not significant.

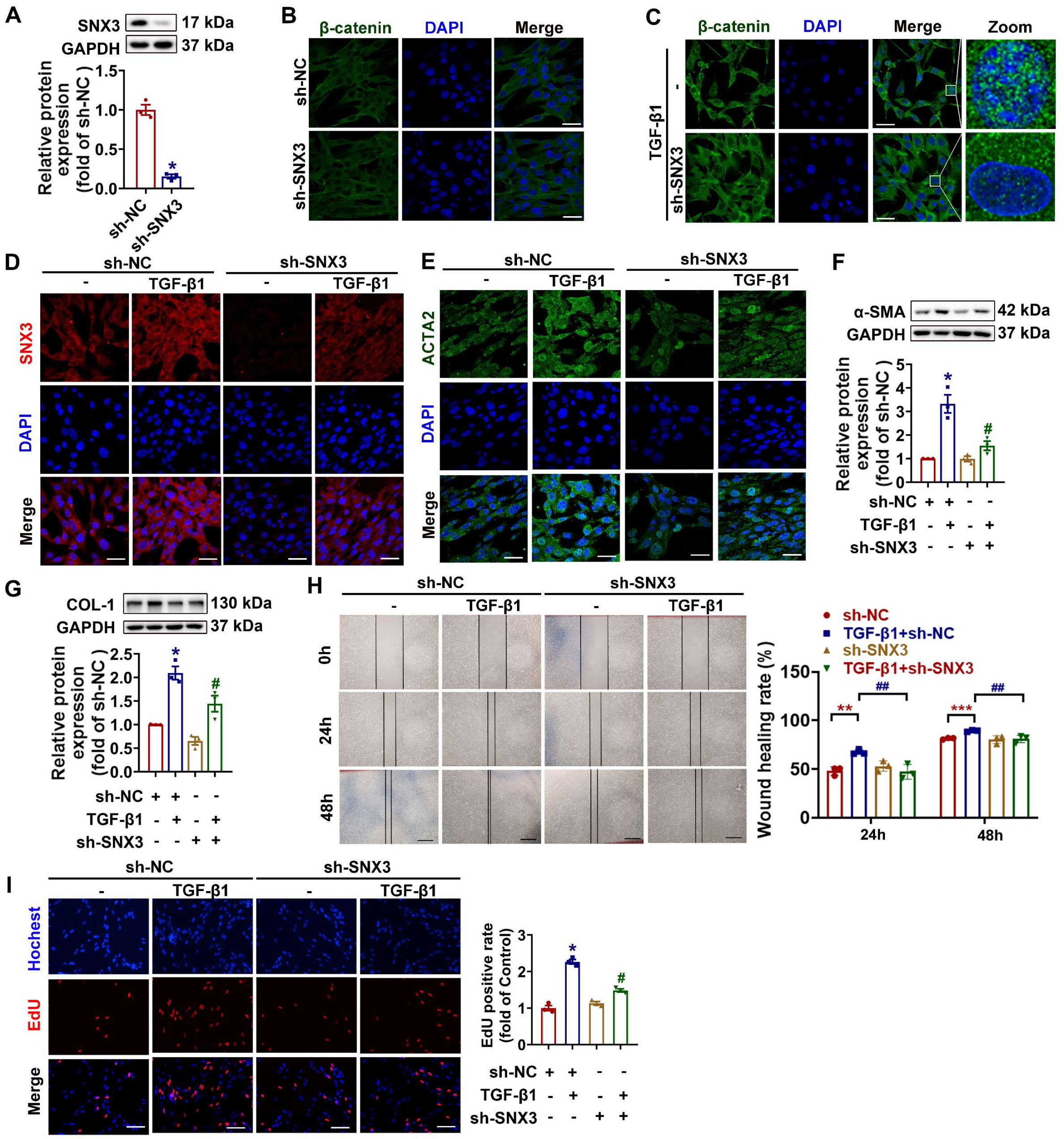

Figure S5 SNX3 deficiency alleviated pulmonary fibrosis in AT2 cells, related to Figure 2. **(A)**, Representative of SNX3 were detected by western blotting analysis; *n*=3 experiments. **(B-C)**, IF staining analysis were shown; Scale bar: 25 µm, *n*=3 experiments. **(D-E)**, IF staining analysis of SNX3 and ACTA2 were shown; Scale bar: 25µm, *n*=3 experiments. **(F-G)**, Representative images of western blotting analysis indicted that the protein levels of α-SMA and COL-1 were shown; *n*=3 experiments. **(H-I)**, Wound healing analysis and EdU analysis were shown; Scale bar: 200µm, *n*=3 experiments. The data were shown as means ± SEM. **P* < 0.05 vs. sh-NC. #*P* < 0.05 vs sh-NC+ TGF-β1. ns, not significant.

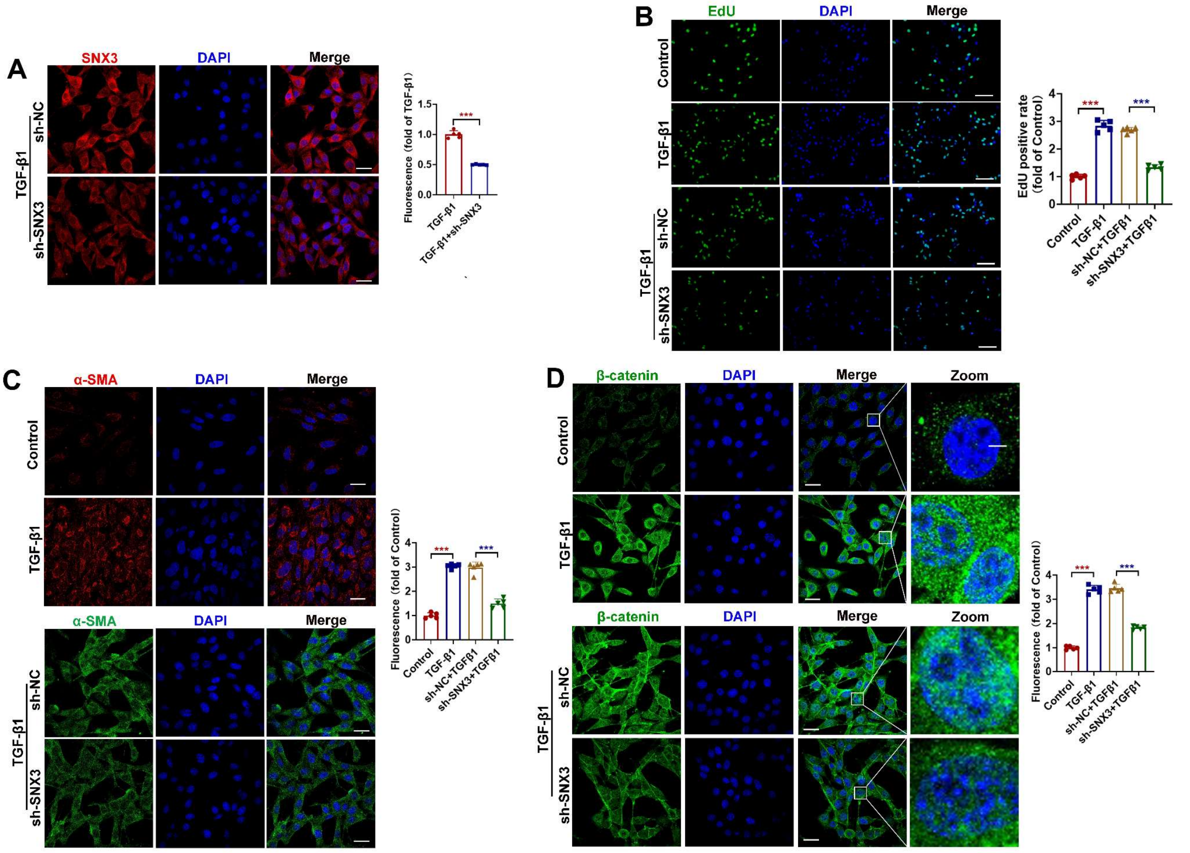

Figure S6 SNX3 deficiency alleviated pulmonary fibrosis in FB cells*,* related to Figure 2.

(A), Representative images of immunofluorescence (IF) staining illustrating the protein level of SNX3 (Scale bar: 25μm μm). **(B-D)**, Representative IF images demonstrating SNX3 knockdown via targeted shRNA in TGF-β1-activated FBs significantly attenuated EdU incorporation (Scale bar: 200 μm), α-SMA, and β-catenin expression relative to scrambled shRNA controls (Scale bar: 25 μm)., n=3 experiments.

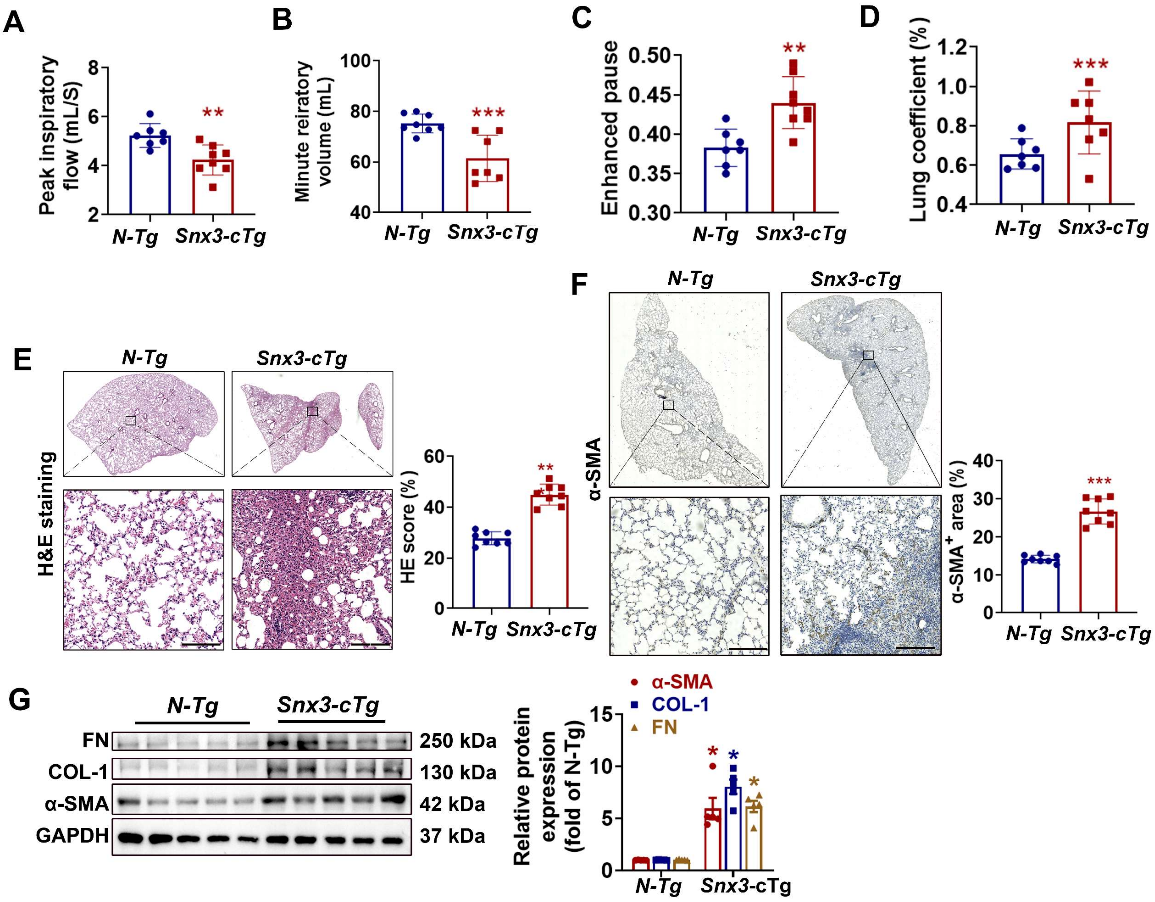

Figure S7 Overexpression of SNX3 caused pulmonary fibrosis *in vivo*, related to Figure 3. **(A-B)**, The scheme of *Snx3-cTg* mice construction strategy and breeding process was shown. **(C)**, The plasmid profiles were presented. **(D)**, PCR electrophoresis of F0 generation mice. Marker. **(E)**, The plasmid profiles of vector carrying mouse *Sftpc-e(IRES-CreERT2)* cDNA were presented. **(F)**, The *Snx3-Tg* genotype was identified. **(G)**, The *Sftpc-e(IRES-CreERT2)* genotype was identified by PCR. **(H)**, Gross observation of lung was shown. **(I-K)**, Peak inspiratory flow, Enhanced paused and Minute respiratory were measured by EMKA system; *n*=8 mice. **(L)**, The lung coefficient ratio was calculated; *n*=8 mice. **(M)**, H&E staining of lung tissue sections were shown; Scale bar: 200 μm, *n*=8 mice. **(N)**, Representative images of IHC staining analysis in *Snx3-Tg* mice were shown; Scale bar: 200 μm, *n*=8 mice. **(O)**, The protein levels of FN, COL-1 and α-SMA were detected by western blot analysis; *n*=8 mice. The data were shown as means ± SEM. **P* < 0.05 vs. *N-Tg*. ns, not significant.

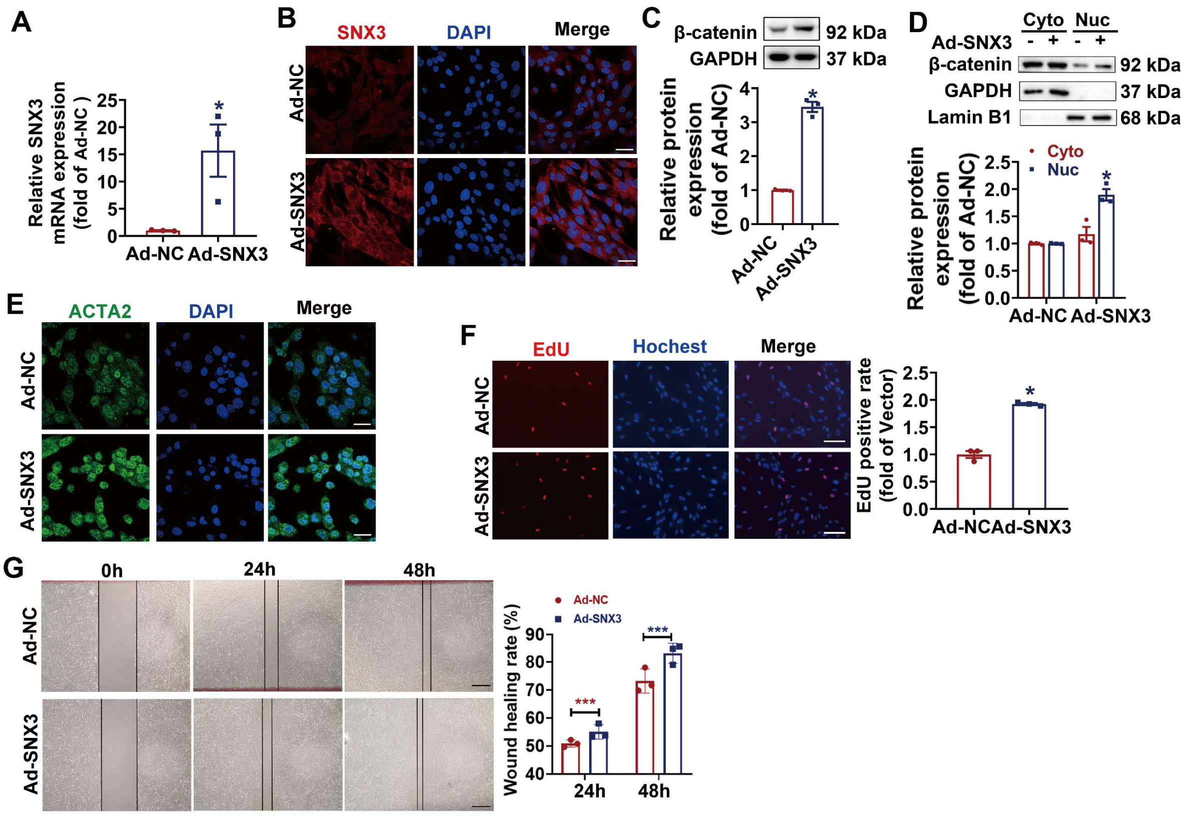

Figure S8 Overexpression of SNX3 caused pulmonary fibrosis in AT2 cells, related to Figure 3.

(A), qPCR results of SNX3 were shown, *n*=3 experiments. **(B)**, IF staining analysis of SNX3 were shown; Scale bar: 25 μm, *n*=3 experiments. **(C-D)**, Western blotting analysis results of β-catenin were shown, *n*=3 experiments. **(E)**, IF staining analysis detected that ACTA2 positive cells were shown; Scale bar: 25 μm, *n*=3 experiments. **(F-G)**, EdU analysis and wound healing analysis were shown; Scale bar: 200μm, *n*=3 experiments. The data were shown as means ± SEM. *$P < 0.05$ vs. Ad-NC. ns, not significant.

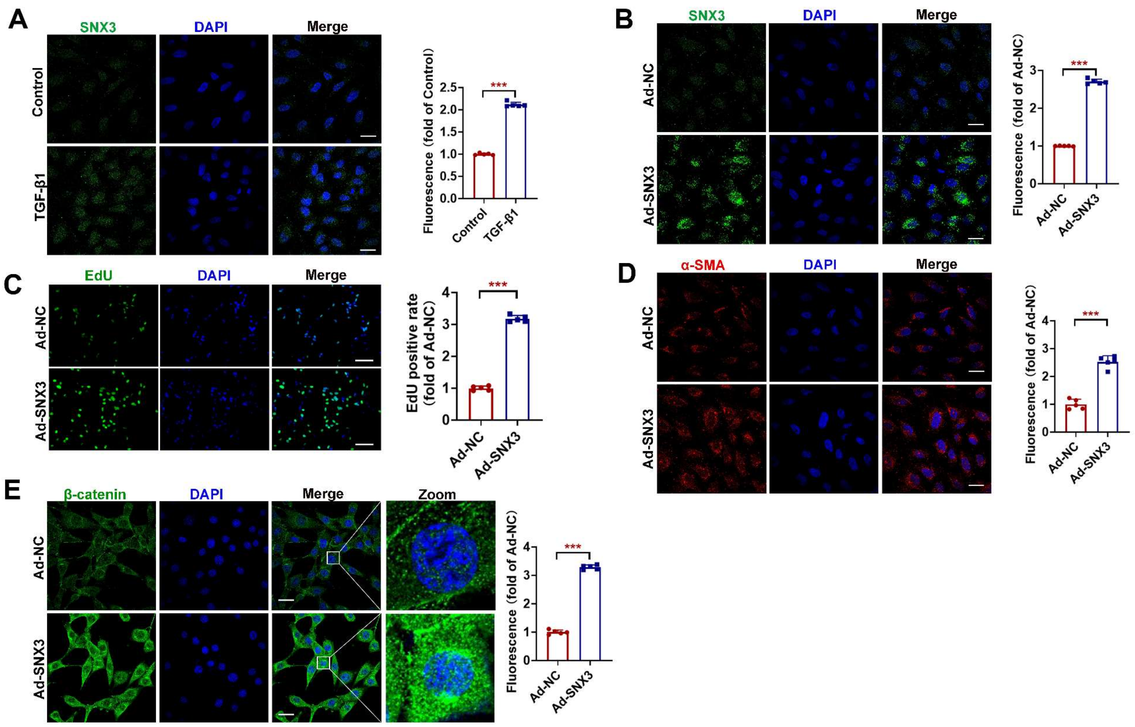

Figure S9 Overexpression of SNX3 caused pulmonary fibrosis in FB cells, related to Figure 3.

(A), Representative images of immunofluorescence (IF) staining illustrating the protein level of SNX3 (Scale bar: 25 μm). **(B-E),** Representative IF images demonstrating Ad-SNX3-infected FB cells exhibited enhanced SNX3 overexpression, accompanied by increased EdU incorporation (Scale bar: 200 μm), elevated α-SMA, and β-catenin accumulation compared to vector controls (Scale bar: 25 μm). n=3 experiments.

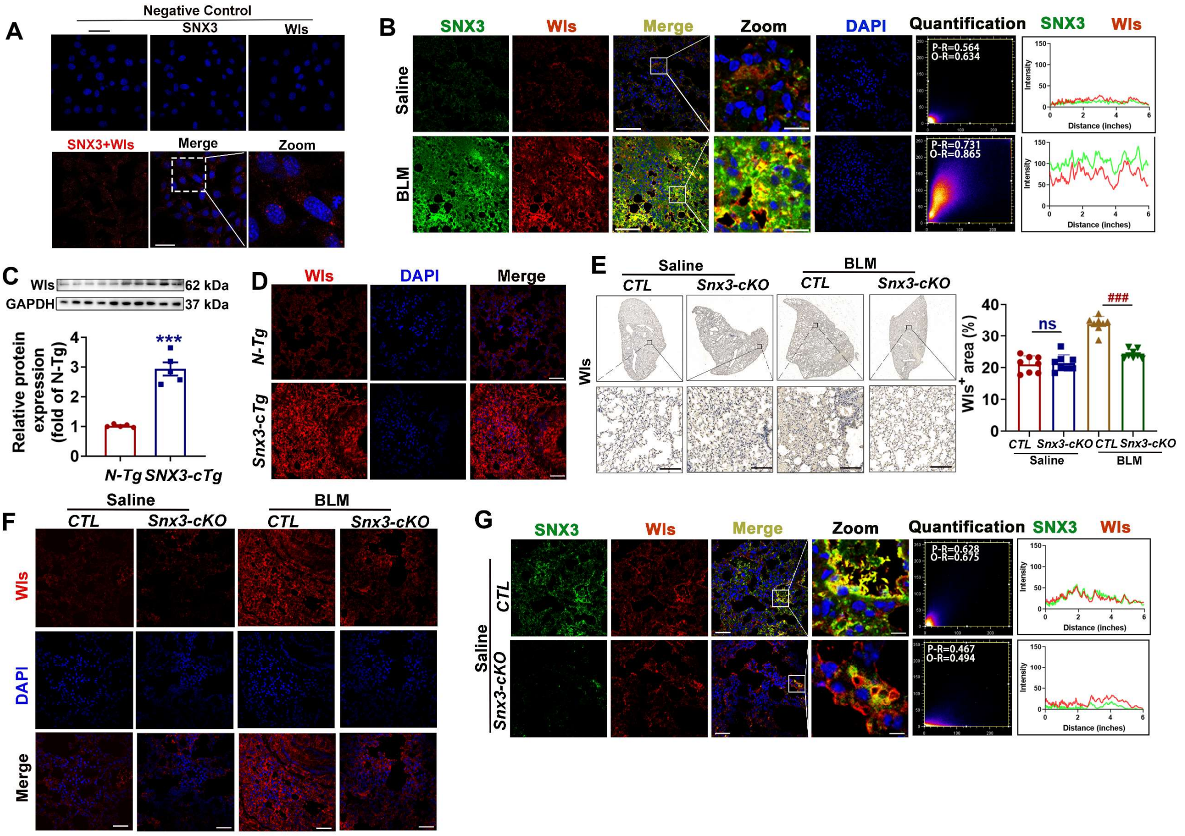

Figure S10 SNX3 interacted with Wls and regulated its intracellular recycling and degradation to activate Wnt/β-catenin pathway, related to Figure 4.

(A), The intracellular colocalization of SNX3 with Wls ((red) was identified by PLA analysis (scale bar: 20 μm). **(B)**, The protein level of Wls and colocalization with SNX3 were presented by IF staining analysis (Scale bar: 100 μm, *n*=8 mice). **(C-D)**, Representative western blotting analysis (*n*=8 mice) and IF staining analysis (Scale bar: 100 μm, *n*=8 mice) images of Wls were presented in *Snx3-cTg* mice. **(E-F)**, Representative IHC staining analysis (Scale bar: 200 μm, *n*=8 mice) and IF staining analysis images (Scale bar: 100 μm, *n*=8 mice) of Wls were presented in *Snx3-cKO* mice. **(G)**, The protein level of Wls and colocalization with SNX3 in *Snx3-cKO* mice were shown in IF staining analysis (Scale bar: 100 μm, *n*=8 mice). The data were shown as means ± SEM. **P* < 0.05 vs. *CTL+ Saline* group *or* sh-NC. #*P* < 0.05 vs *CTL+BLM* group *or* sh-NC+TGF-β1. ns, not significant.

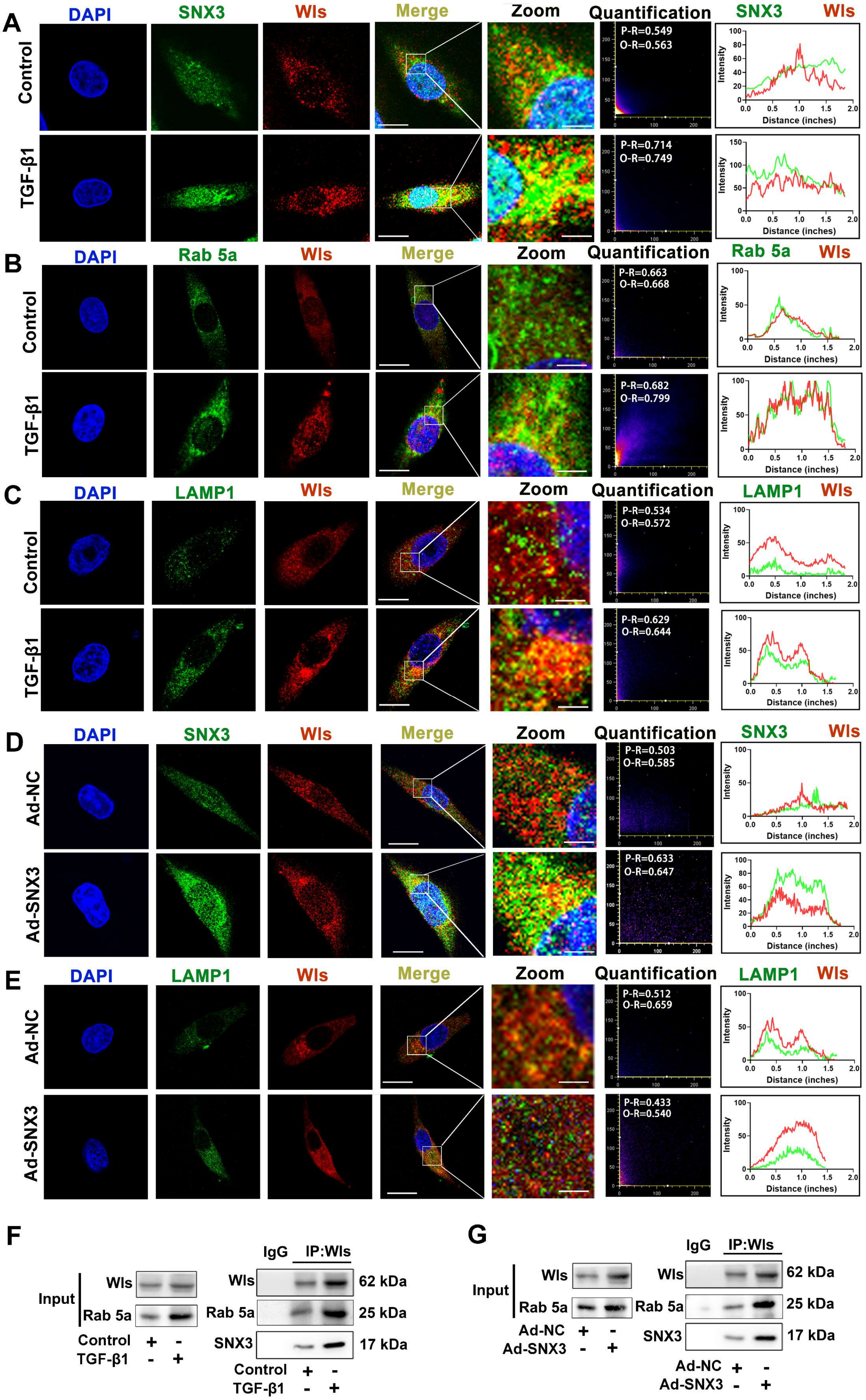
A
DAPI
SNX3
Wls
Merge
Zoom
Quantification
Control
TGF-β1
P-R=0.549
O-R=0.563
P-R=0.714
O-R=0.749
Intensity
Distance (inches)
B
Rab 5a
P-R=0.663
O-R=0.668
P-R=0.682
O-R=0.799
C
LAMP1
P-R=0.534
O-R=0.572
P-R=0.629
O-R=0.644
D
Ad-NC
Ad-SNX3
P-R=0.503
O-R=0.585
P-R=0.633
O-R=0.647
E
P-R=0.512
O-R=0.659
P-R=0.433
O-R=0.540
F
Input
IgG
IP:Wls
62 kDa
25 kDa
17 kDa
G

Figure S11 SNX3 interacted with Wls and regulated its intracellular recycling and degradation to activate Wnt/β-catenin pathway, related to Figure 4.

(A-E), The intracellular colocalization and quantitative of colocalization of Wls and SNX3, Rab 5a, LAMP1 were detected by IF staining analysis. Representative images were shown; Scale bar: 10 μm, *n*=3 experiments. **(F-G)**, Enhanced Wls-Rab5a binding following TGF-β1 stimulation or SNX3 overexpression by Co-IP analysis, *n*=3 experiments.

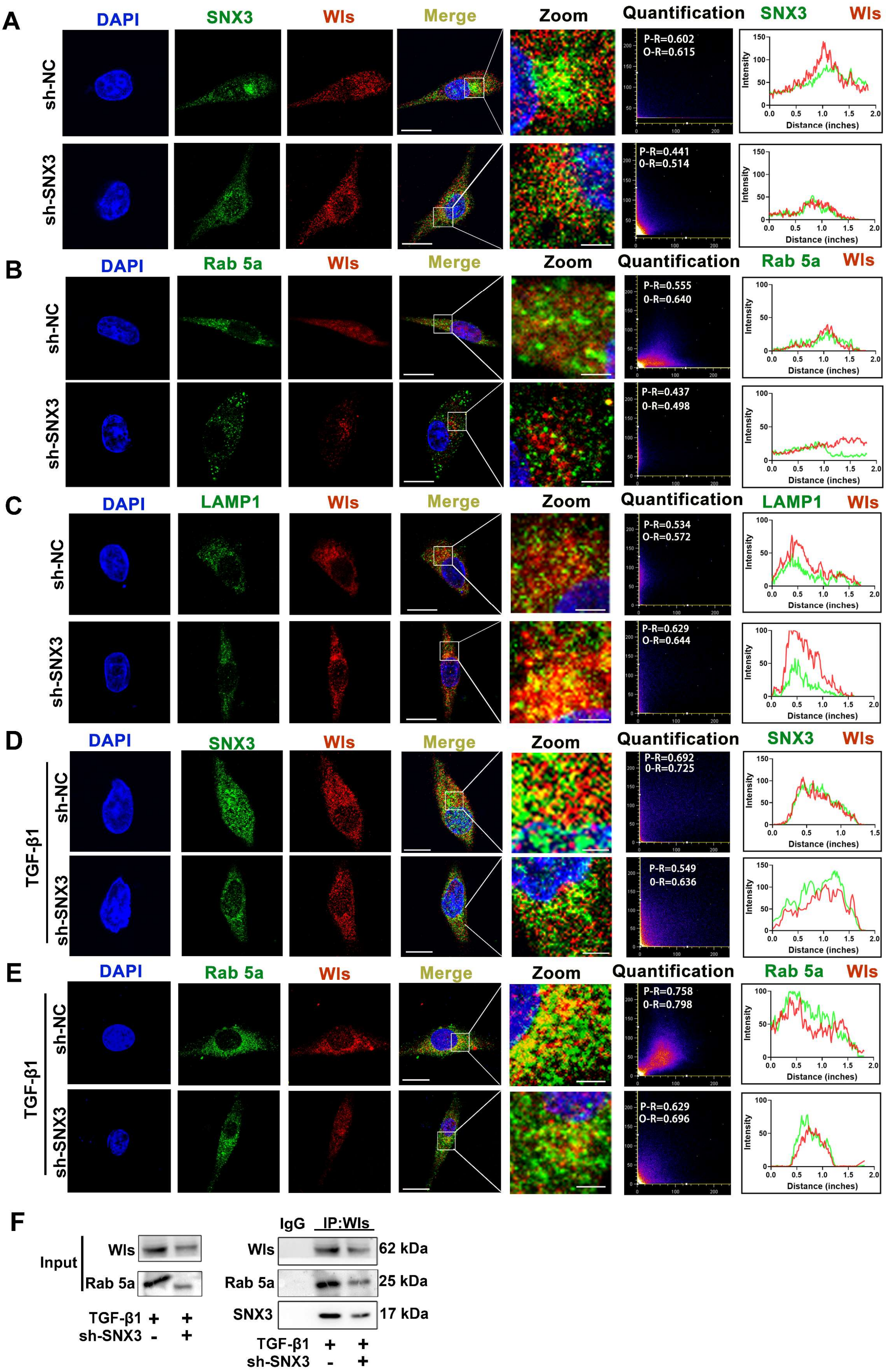
A
DAPI
SNX3
Wls
Merge
Zoom
Quantification
sh-NC
sh-SNX3
P-R=0.602
O-R=0.615
P-R=0.441
O-R=0.514
Intensity
Distance (inches)
B
Rab 5a
P-R=0.555
O-R=0.640
P-R=0.437
O-R=0.498
C
LAMP1
P-R=0.534
O-R=0.572
P-R=0.629
O-R=0.644
D
TGF-β1
P-R=0.692
O-R=0.725
P-R=0.549
O-R=0.636
E
P-R=0.758
O-R=0.798
P-R=0.629
O-R=0.696
F
Input
IgG
IP:Wls
62 kDa
25 kDa
17 kDa
TGF-β1
sh-SNX3

Figure S12 SNX3 interacted with Wls and regulated its intracellular recycling and degradation to activate Wnt/β-catenin pathway, related to Figure 4.

(A-E), the intracellular colocalization and quantitative of colocalization of Wls and SNX3, Rab 5a, LAMP1 were detected by IF staining analysis. Representative images were shown; Scale bar: 10 μm, *n*=3 experiments. **(F)**, SNX3 knockdown disrupted Wls-Rab5a interaction by Co-IP analysis, *n*=3 experiments.

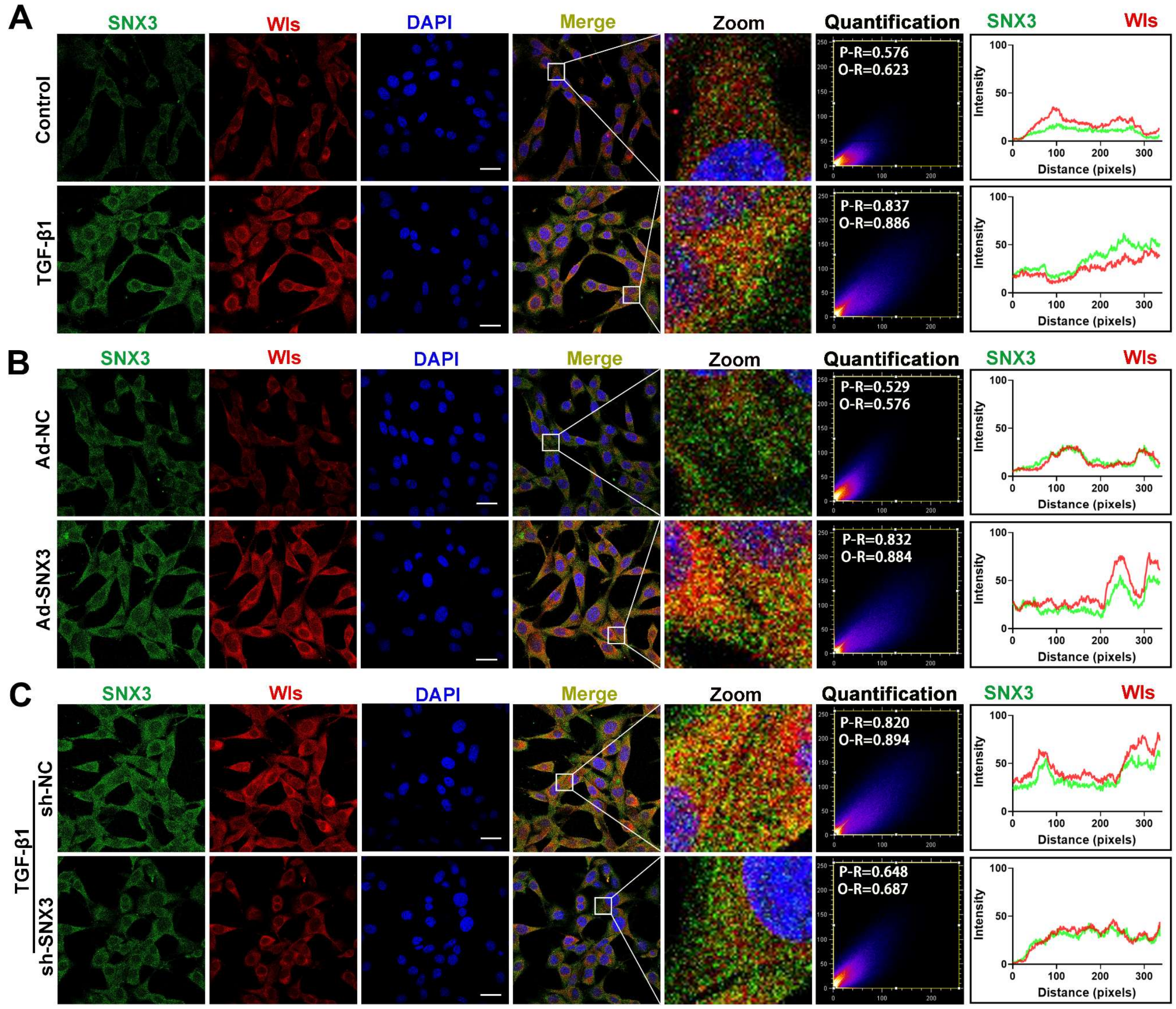

Figure S13 The interaction of SNX3 and Wls in FB cells, related to Figure 4.

(A), Immunofluorescence analysis demonstrating enhanced co-localization (yellow) of SNX3 (green) and Wls (red) following TGF-β1 stimulation. Nuclei counterstained with DAPI (blue). **(B)**, Ectopic SNX3 overexpression amplified SNX3-Wls interaction, evidenced by increased Pearson correlation coefficient (P-R=0.832 *vs.* 0.529 in controls). **(C)**, SNX3 knockdown (siRNA) substantially attenuated interaction, reducing P-R to 0.648. Scale bar: 25 μm. n=3 experiments.

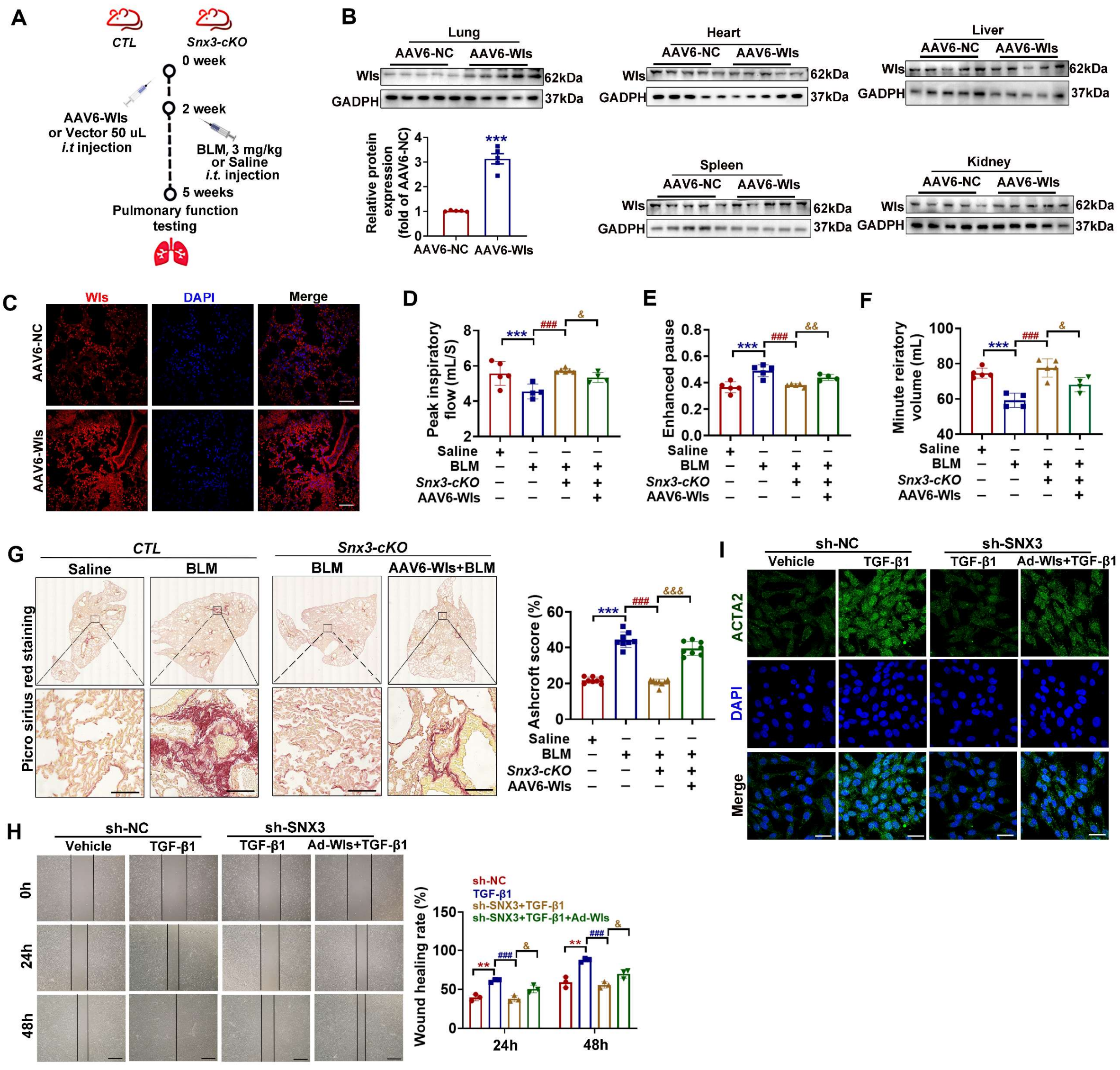

Figure S14 SNX3 interacted with Wls and regulated its intracellular recycling and degradation to activate Wnt/β-catenin pathway, related to Figure 4.

(A), *Snx3-cKO* mice and *CTL* mice were administered AAV6-Wls (50μL, *i.p* injection) and BLM (3 mg/kg, intratracheal instillation). **(B-C)**, Protein expression of Wls in lung, heart, liver, spleen, and kidney tissues of AAV6-Wls and negative control (AAV6-NC) mice was determined by Western blot and immunofluorescence images. Scale bar: 100 μm. *n*=8 mice. **(D-F)**, Peak inspiratory flow, Enhanced pause and Minute respiratory volume were detected by the EMKA system; *n*=8 mice. **(G)**, PSR staining of lung tissues are shown; Scale bar: 200 μm, *n*=8 mice. **(H)**, Wound healing analysis were shown; Scale bar: 200μm, *n*=3 experiments. **(I)**, IF staining analysis detected that ACTA2 positive cells were shown; Scale bar: 25 μm, *n* =3 experiments. The data were shown as means ± SEM. $^{*}P < 0.05$ vs. *CTL+ Saline* group *or* sh-NC. $^{\#}P < 0.05$

vs *CTL+BLM* group *or* sh-NC+TGF-β1. [&]$P < 0.05$ vs *Snx3-cKO+BLM* group *or* sh-SNX3+ TGF-β1. ns, not significant.

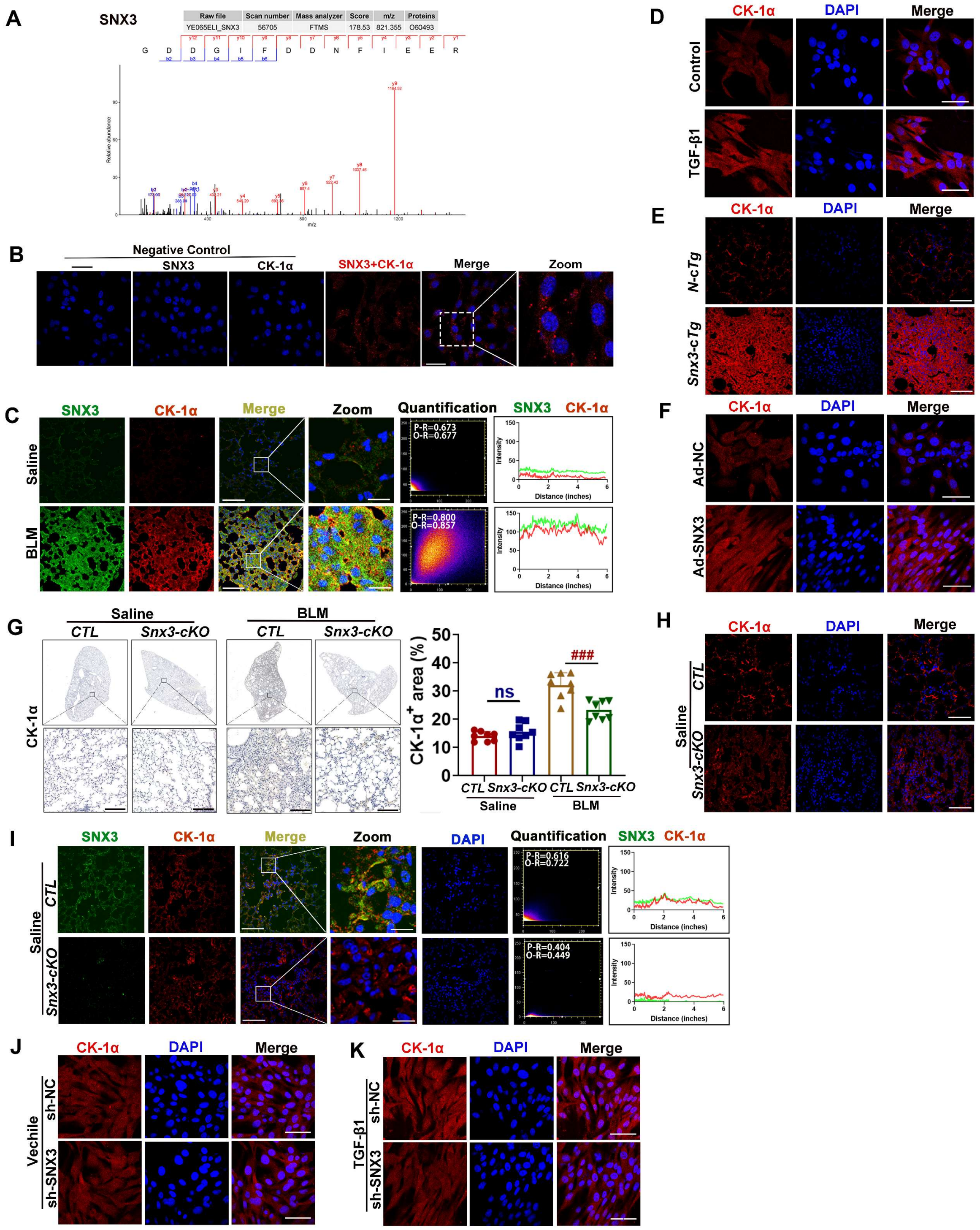

Figure S15 CK-1α was identified as a novel cargo protein of SNX3 in pulmonary fibrosis, related to Figure 5.

(A), The protein mass spectrum of SNX3 were shown. **(B)**, The intracellular colocalization of SNX3 with CK-1a((red) was identified by PLA analysis (scale bar: 20 μm). **(C)**, Representative IF staining analysis of CK-1α and colocalization with SNX3 in BLM-induced mice were shown (Scale bar: 100 μm, *n*=8 mice). **(D)**, Representative IF staining analysis images of CK-1α (Scale

bar: 25 μm, *n*=3 experiments). **(E)**, Representative IF staining analysis images of CK-1α in *Snx3-cTg* mice were shown; Scale bar: 100 μm, *n*=8 mice. **(F)**, Representative IF staining analysis (Scale bar: 100 μm, *n*=8 mice) were shown. **(G)**, Representative images of IHC staining analysis for CK-1α in lung tissue sections as indicated (Scale bar: 200 μm; *n*=8 mice). **(H-I)**, Representative IF staining analysis of CK-1α and colocalization with SNX3 in *Snx3-cKO* mice were shown (Scale bar: 100 μm, *n*=8 mice). **(J-K)**, Representative IF staining analysis images of CK-1α were presented; Scale bar: 25 μm, *n*=3 experiments. The data were shown as means ± SEM. $^{*}P < 0.05$ vs. *CTL+ Saline* group *or* sh-NC. $^{\#}P < 0.05$ vs *CTL+BLM* group *or* sh-NC+TGF-β1. ns, not significant.

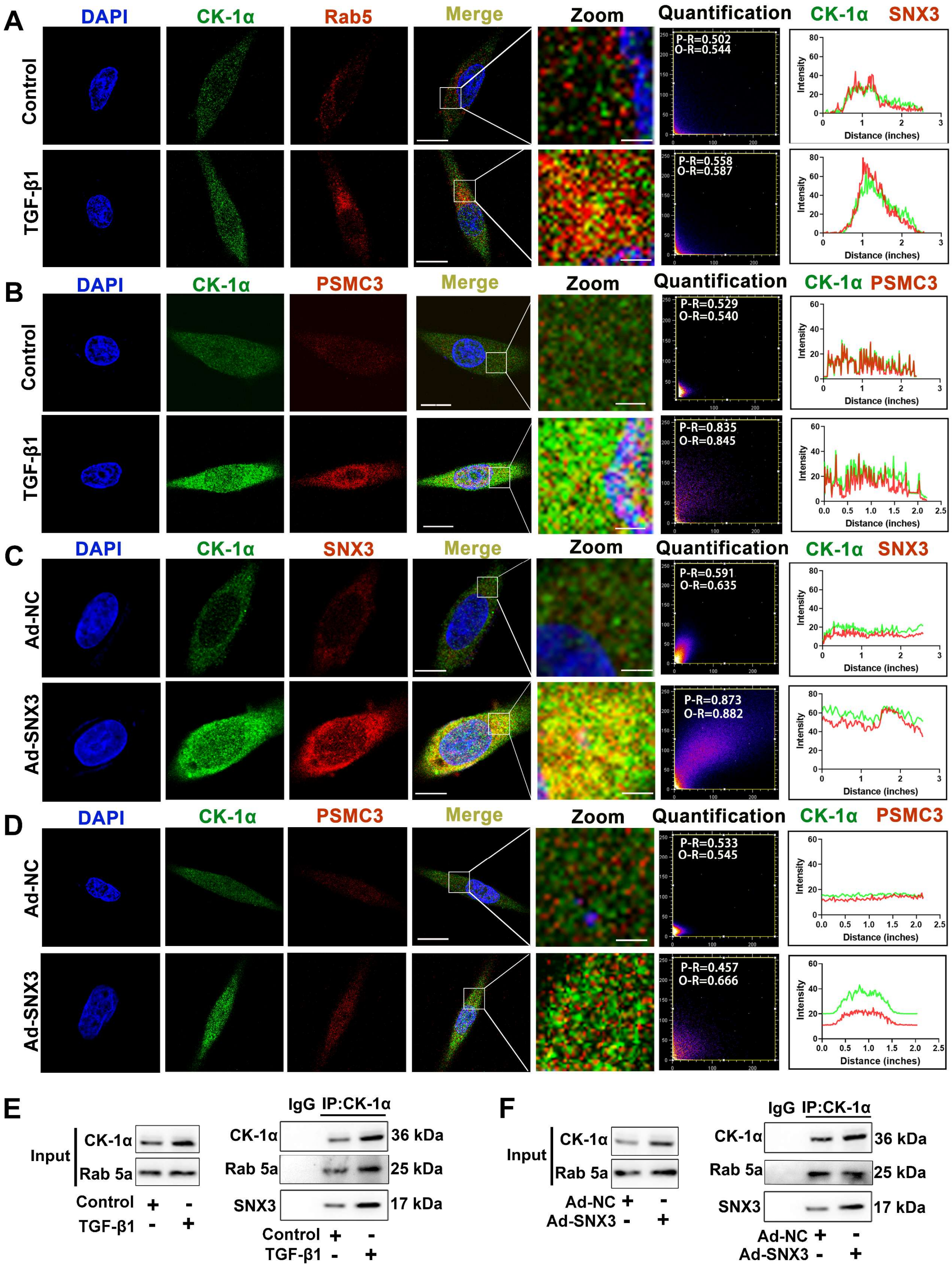

Figure S16 CK-1α was identified as a novel cargo protein of SNX3 in pulmonary fibrosis, related to Figure 5.

(A-D), The intracellular colocalization and quantitative of colocalization of CK-1α and SNX3,

Rab 5a, PSMC3 were detected by IF staining analysis. Representative images were shown; Scale bar: 10 μm, *n*=3 experiments. **(E-F)**, Enhanced CK-1α-Rab5a binding following TGF-β1 stimulation or SNX3 overexpression by Co-IP analysis, *n*=3 experiments.

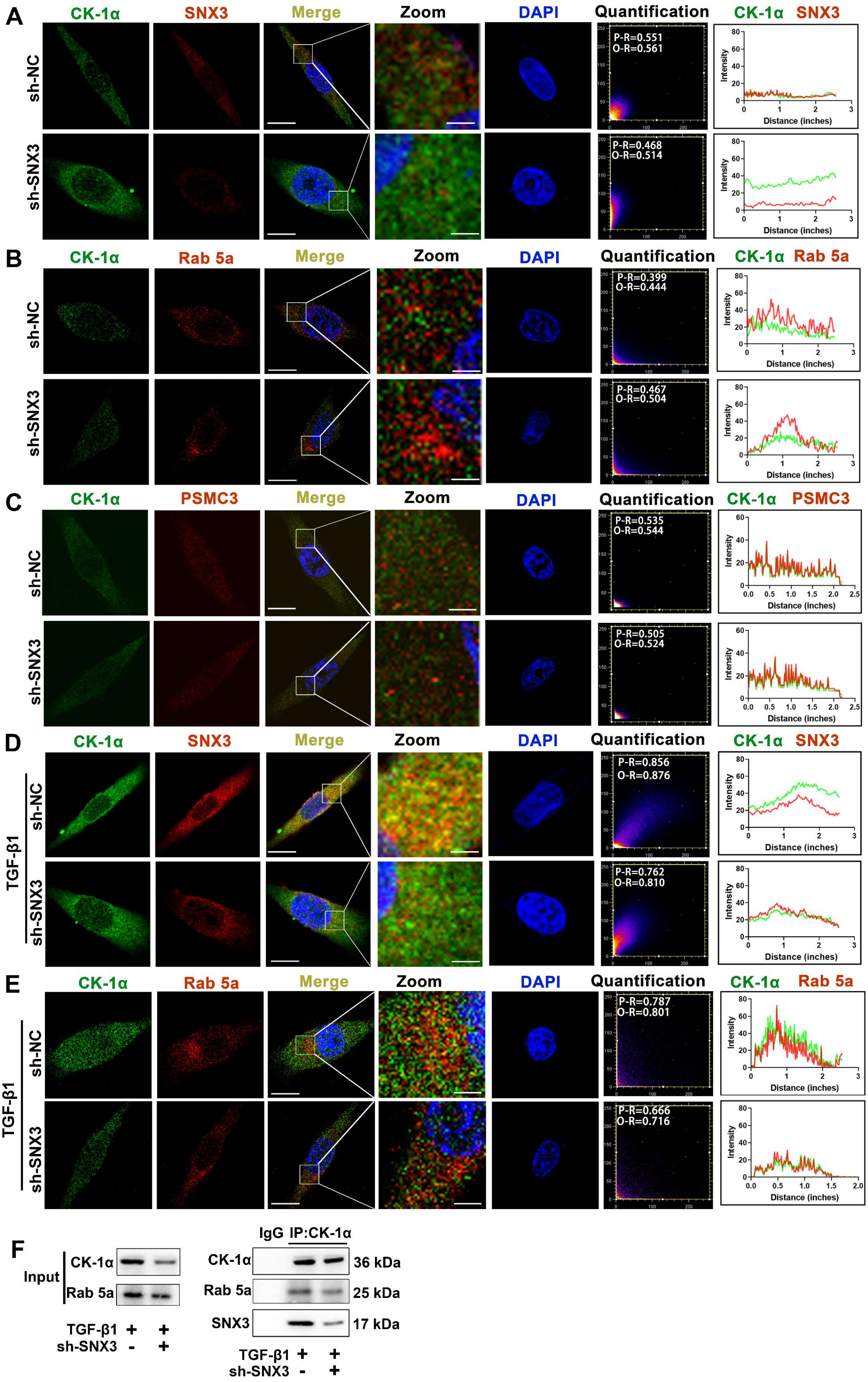
A
CK-1α
SNX3
Merge
Zoom
DAPI
Quantification
CK-1α SNX3
sh-NC
sh-SNX3
P-R=0.551
O-R=0.561
P-R=0.468
O-R=0.514
Intensity
Distance (inches)
B
CK-1α
Rab 5a
Merge
Zoom
DAPI
Quantification
CK-1α Rab 5a
sh-NC
sh-SNX3
P-R=0.399
O-R=0.444
P-R=0.467
O-R=0.504
C
CK-1α
PSMC3
Merge
Zoom
DAPI
Quantification
CK-1α PSMC3
sh-NC
sh-SNX3
P-R=0.535
O-R=0.544
P-R=0.505
O-R=0.524
D
CK-1α
SNX3
Merge
Zoom
DAPI
Quantification
CK-1α SNX3
TGF-β1
sh-NC
sh-SNX3
P-R=0.856
O-R=0.876
P-R=0.762
O-R=0.810
E
CK-1α
Rab 5a
Merge
Zoom
DAPI
Quantification
CK-1α Rab 5a
TGF-β1
sh-NC
sh-SNX3
P-R=0.787
O-R=0.801
P-R=0.666
O-R=0.716
F
Input
CK-1α
Rab 5a
TGF-β1 + +
sh-SNX3 - +
IgG IP:CK-1α
CK-1α 36 kDa
Rab 5a 25 kDa
SNX3 17 kDa
TGF-β1 + +
sh-SNX3 - +

Figure S17 CK-1α was identified as a novel cargo protein of SNX3 in pulmonary fibrosis, related to Figure 5.

(**A-E)**, The intracellular colocalization and quantitative of colocalization of CK-1α and SNX3, Rab 5a, PSMC3 were detected by IF staining analysis. Representative images were shown; Scale bar: 10 μm, *n*=3 experiments. **(F)**, SNX3 knockdown disrupted CK-1α-Rab5a interaction by Co-IP analysis, *n*=3 experiments.

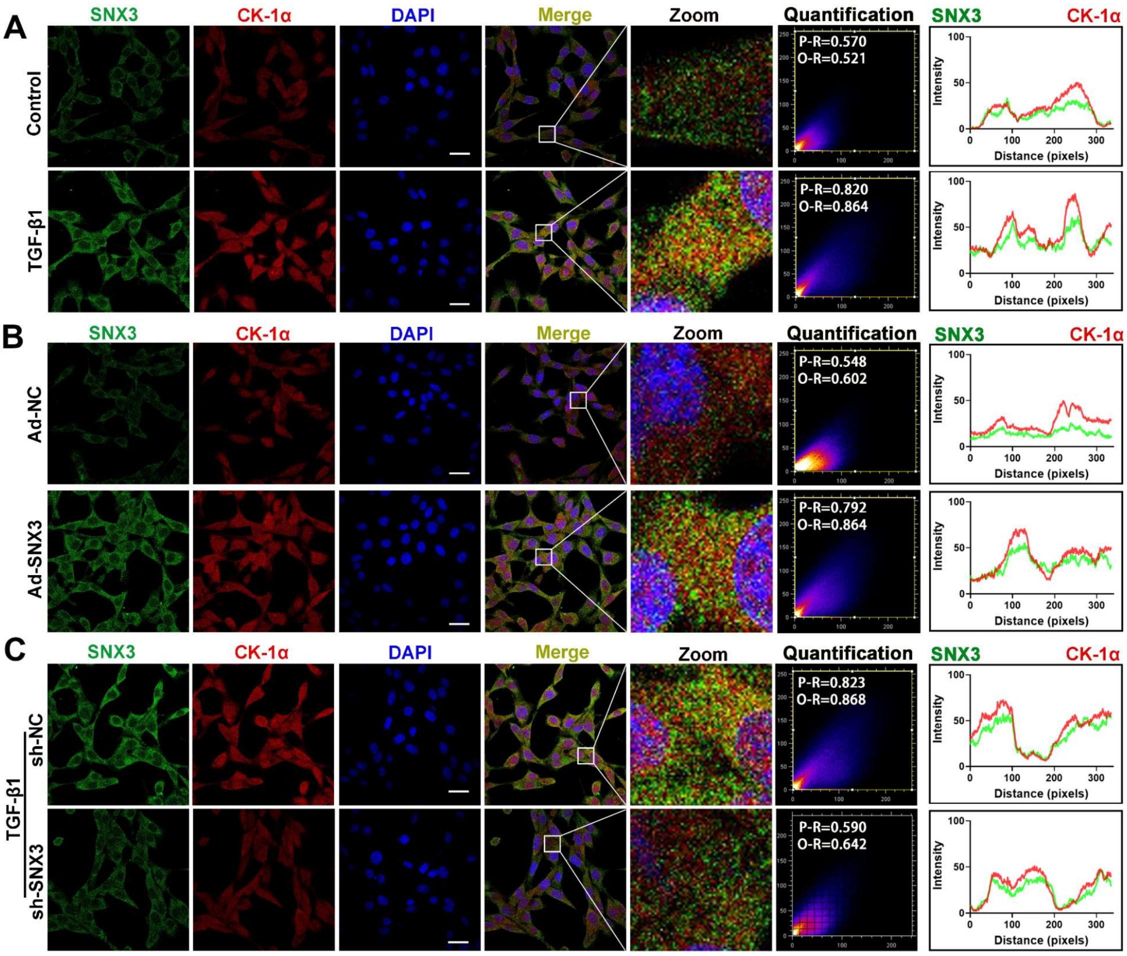

Figure S18 The interaction of SNX3 and CK-1α in FB cells, related to Figure 5.

(A), Immunofluorescence analysis demonstrating enhanced co-localization (yellow) of SNX3 (green) and CK-1α (red) following TGF-β1 stimulation. Nuclei counterstained with DAPI (blue). Scale bar: 25 μm. **(B)**, Ectopic SNX3 overexpression amplified SNX3-CK-1α interaction, evidenced by increased Pearson correlation coefficient (P-R=0.792 *vs.* 0.548 in controls). **(C)**, SNX3 knockdown (siRNA) substantially attenuated interaction, reducing P-R to 0.590. Scale bar: 25 μm. n=3 experiments.

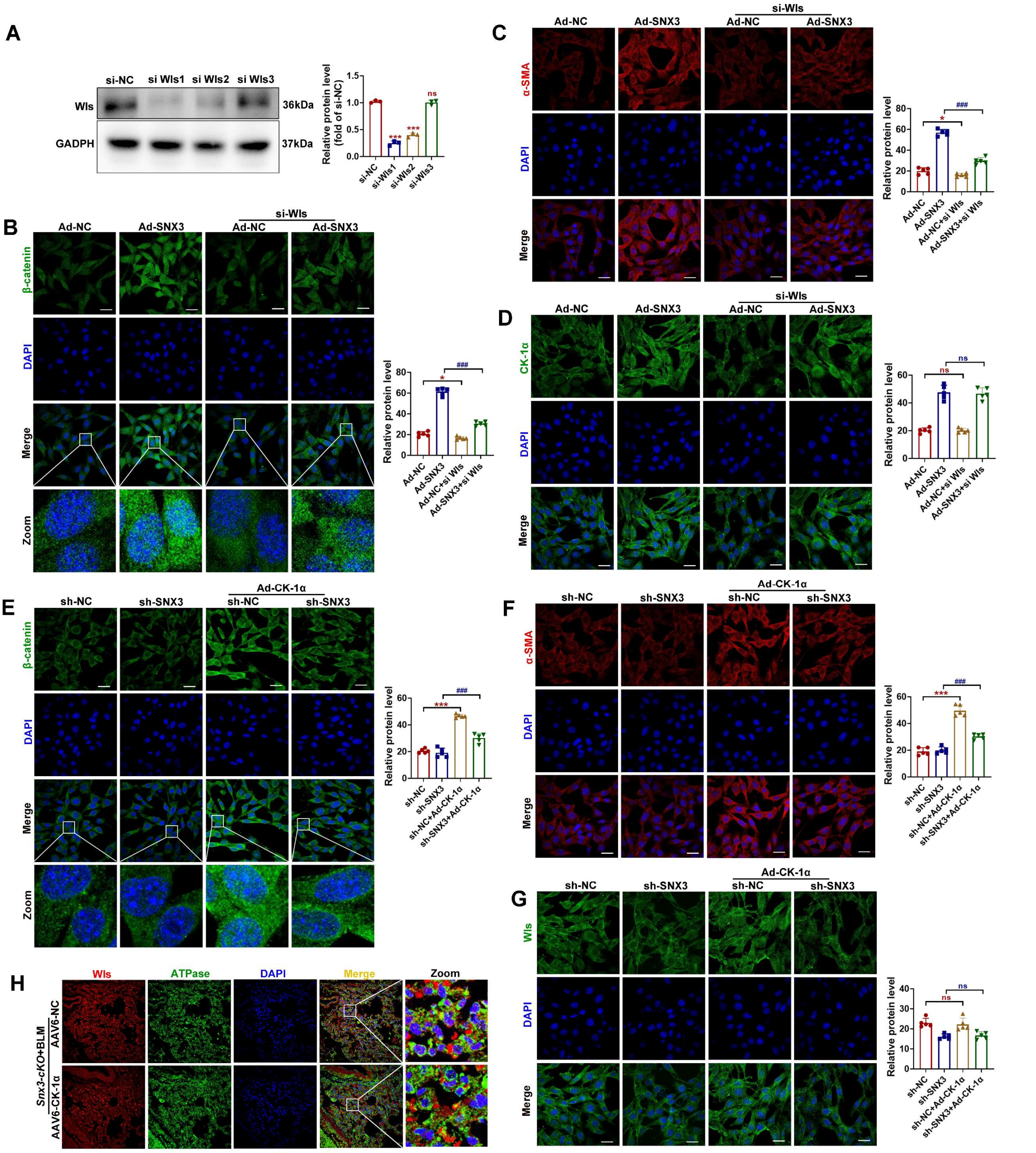

Figure S19 Compartmentalized regulation of Wls and CK-1α by SNX3 in AT2 cells and Snx3-cKO mice, related to Figure 5.

(A), Wls knockdown validation in AT2 cells via siRNA. **(B-D)**, Immunofluorescence (IF) of β-catenin, α-SMA and CK-1α in AT2 cells overexpressing SNX3 via Ad-SNX3 infection in the presence or absence of Wls knockdown. **(E-G)**, IF of β-catenin, α-SMA and Wls in AT2 cells following SNX3 knockdown with or without CK-1α overexpression. **(H)**, Co-localization IF of Wls and ATPase in lung sections from Snx3-cKO mice after BLM challenge with or without

AAV6-CK-1α administration. Nuclei counterstained with DAPI (blue). Scale bars: 100 μm. n=3 independent experiments (A-H); n= 8 mice (I).

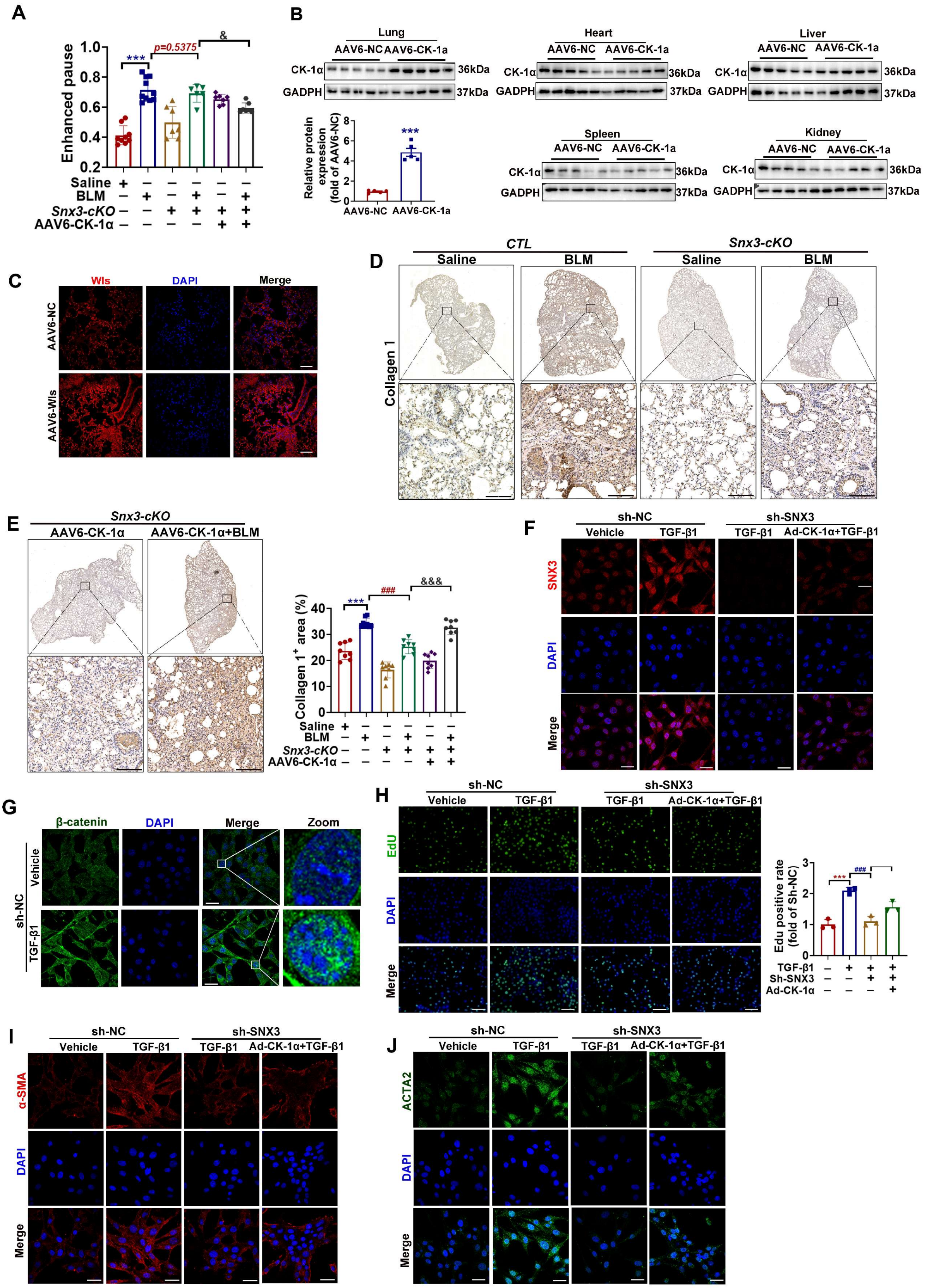

Figure S20 SNX3/CK-1α axis induced Wnt/β-catenin signaling pathway activation and pulmonary fibrosis, related to Figure 6.

(A), Enhanced pause was detected as detected by the EMKA system; *n*=8 mice. **(B-C)**, Protein expression of CK-1α in lung, heart, liver, spleen, and kidney tissues of AAV6-CK-1α and negative control (AAV6-NC) mice was determined by Western blot and immunofluorescence (IF) images Scale bar: 100 μm. *n*=8 mice. **(D-E)**, Representative images of IHC staining analysis for Collagen 1 in lung tissue sections as indicated (Scale bar: 200 μm; *n*=8 mice). **(F)**, Representative IF staining analysis images of SNX3 (Scale bar: 25 μm, *n*=3 experiments). **(G)**, The protein level and nuclear distribution of β-catenin were measured by IF staining analysis; Scale bar: 25 μm, *n*=3 experiments. **(H)**, analysis was shown; Scale bar: 200 μm, *n*=3 experiments. **(I-J)**, IF staining analysis indicated that α-SMA and ACTA2 positive cells; Scale bar: 25 μm, *n*=3 experiments. The data were shown as means ± SEM. $^{*}P < 0.05$ vs. *CTL+ Saline* group *or* sh-NC. $^{\#}P < 0.05$ vs *CTL+BLM* group *or* sh-NC+TGF-β1. $^{\&}P < 0.05$ vs *Snx3-cKO+BLM* group *or* sh-SNX3+ TGF-β1. ns, not significant.

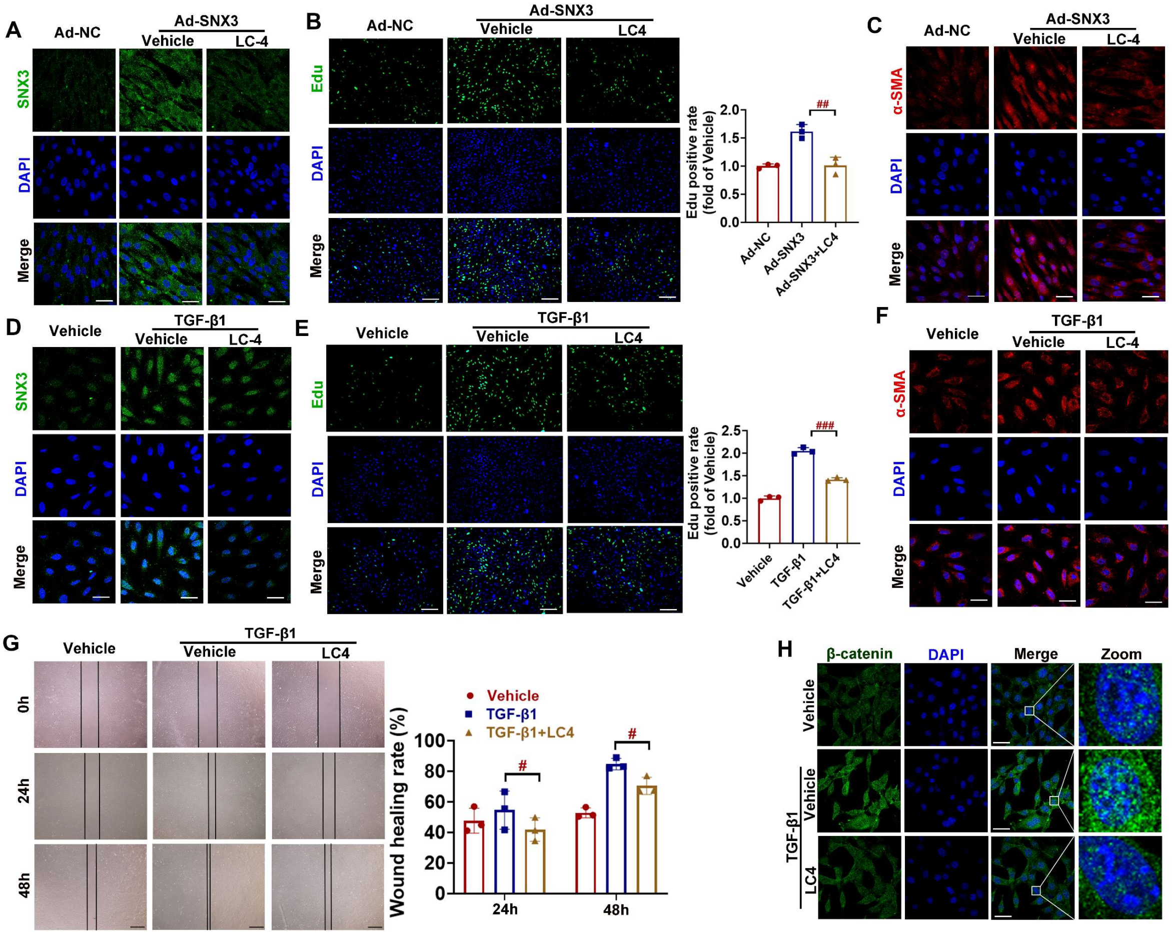

Figure S21 A novel small molecule LC4 targeting SNX3 ameliorated pulmonary dysfunction and fibrosis, related to Figure 7.

(A), Representative images of IF staining analysis as indicated, Scale bar: 25 μm, *n*=3 experiments. **(B)**, The microscopy results of EdU analysis were shown; Scale bar: 200 μm; *n*=3 experiments. **(C-D)**, Representative images of IF staining analysis as indicated, Scale bar: 25 μm, *n* = 3 experiments **(E)**, The microscopy results of EdU analysis were shown; Scale bar: 200 μm; *n*=3 experiments. **(F)**, Representative images of IF staining analysis as indicated, Scale bar: 25 μm, *n*=3 experiments. **(G)**, Representative wound healing analysis images were presented; Scale bar: 200μm, *n*=3 experiments. **(H)**, The protein level and nuclear distribution of β-catenin were measured by IF staining analysis; Scale bar: 25 μm, *n*=3 experiments. The data were shown as means ± SEM. **P* < 0.05 vs. Ad-NC, TDN *or* Vehicle. $^{\#}P < 0.05$ vs Ad-NC *or* TGF-β1. ns, not significant.

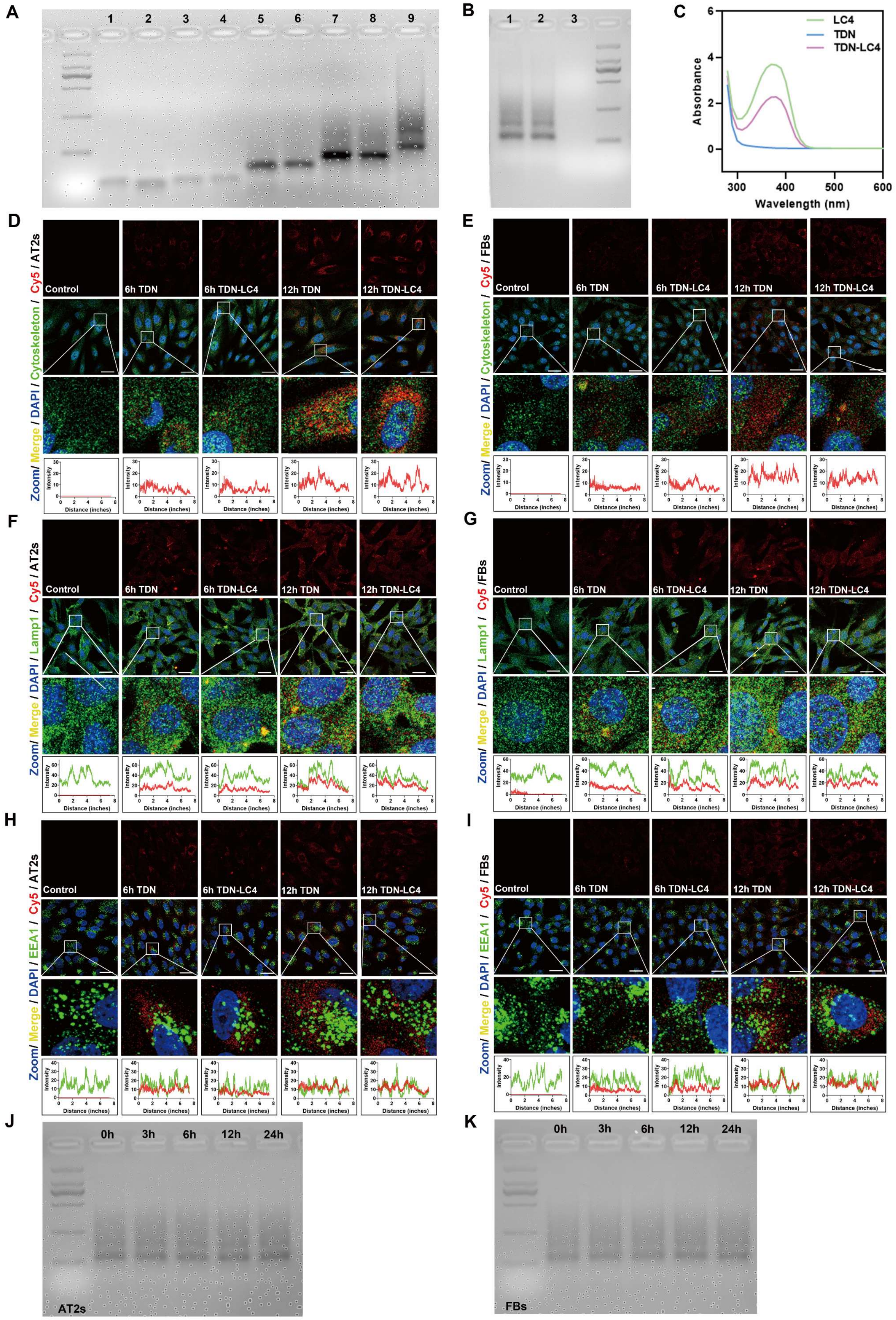

303

304 **Figure S22 Design and characterization of TDN-LC4,** related to Figure 7.

(A), Agarose gel image characterizing TDN-LC4 synthesis (1: S1, 2: S2, 3: S3, 4: S4, 5: S1+S2, 6, S3+S4, 7: S1+S2+S3, 8: S2+S3+S4, 9: S1+S2+S3+S4). **(B)**, Agarose gel image characterizing (1: TDN, 2: TDN-LC4, 3:LC4). **(C)**, UV–vis spectra of LC4, TDN, TDN-LC4. **(D-E)**, IF staining analysis of AT2s and FBs were performed to detect indicating that cells uptake Cy5-labeled TDN and TDN-LC4. Scale bar: 25 μm; *n*=3 experiments. **(F-I)**, IF staining analysis of AT2s and FBs were performed to detect indicating that cells subcellular localization in lysosome (green: Lamp1) and endosome (green: EEA1). Scale bar: 25 μm; *n*=3 experiments. **(J-K)**, The stability of TDN-LC4 in AT2s and FBs.

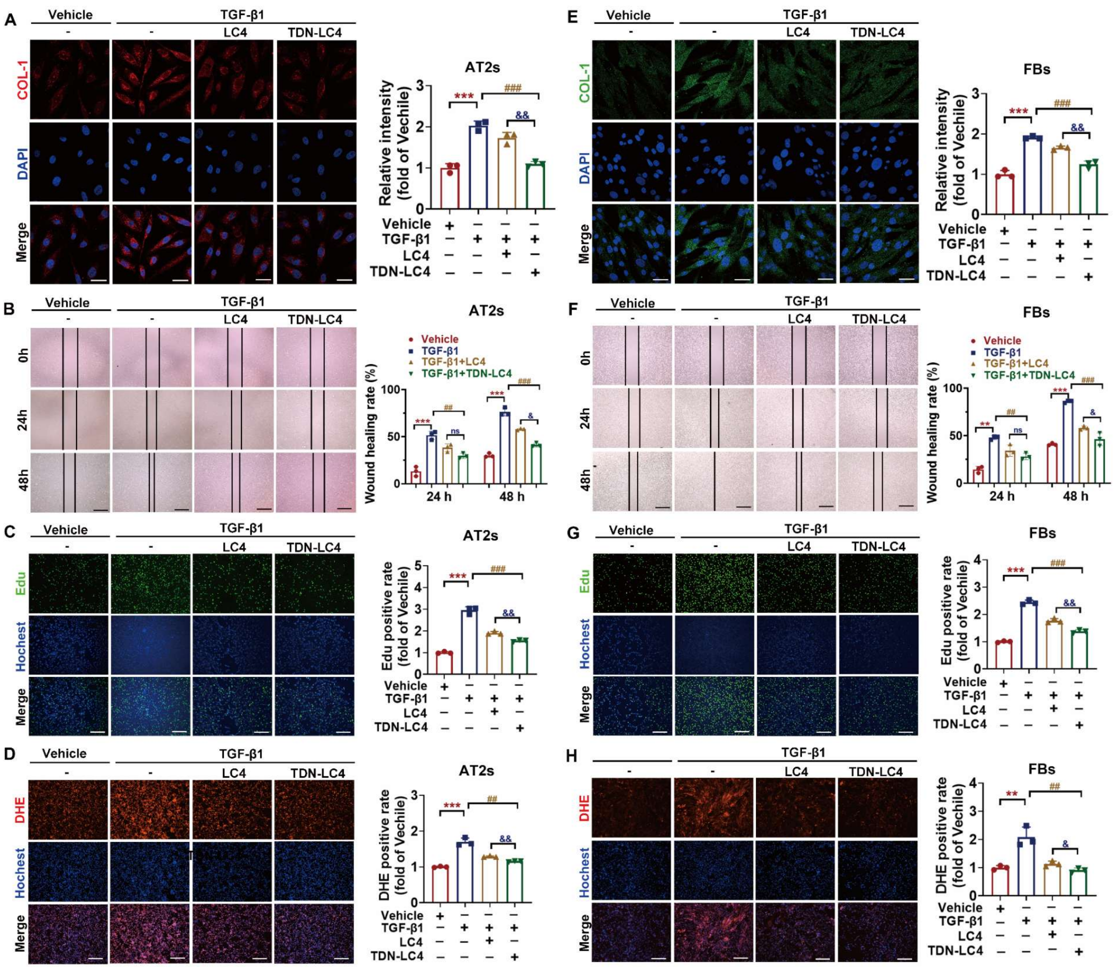

Figure S23 Pulmonary fibrotic progression was rescued via TDN-LC4 *in vitro,* related to Figure 7.

(A), IF staining analysis were performed to detect indicating that COL-1 positive cells in AT2s; Scale bar: 25 μm, *n*=3 experiments. **(B)**, The wound healing analysis detected the migratory ability of in AT2s; Scale bar: 200 μm; *n* = 3 experiments. **(C)**, EdU analysis detected the proliferation ability of in AT2s; Scale bar: 200μm, *n*=3 experiments. **(D)**, DHE analysis detected the ROS levels of AT2s and FBs; Scale bar: 200μm, *n*=3 experiments. **(E)**, IF staining analysis were performed to detect indicating that COL-1 positive cells in FBs; Scale bar: 25 μm, *n*=3 experiments. **(F)**, The wound healing analysis detected the migratory ability of FBs; Scale bar: 200 μm; *n*=3 experiments. **(G)**, EdU analysis detected the proliferation ability of FBs; Scale bar: 200μm, *n*=3 experiments. **(H)**, DHE analysis detected the ROS levels of AT2s and FBs; Scale bar: 200μm, *n*=3 experiments. The data were shown as means ± SEM. *$P < 0.05$ vs. Vehicle. $^{\#}P < 0.05$ vs TGF-β1 *or* TGF-β1+LC4. $^{\&}P < 0.05$ vs LC4+TGF-β1 ns, not significant.

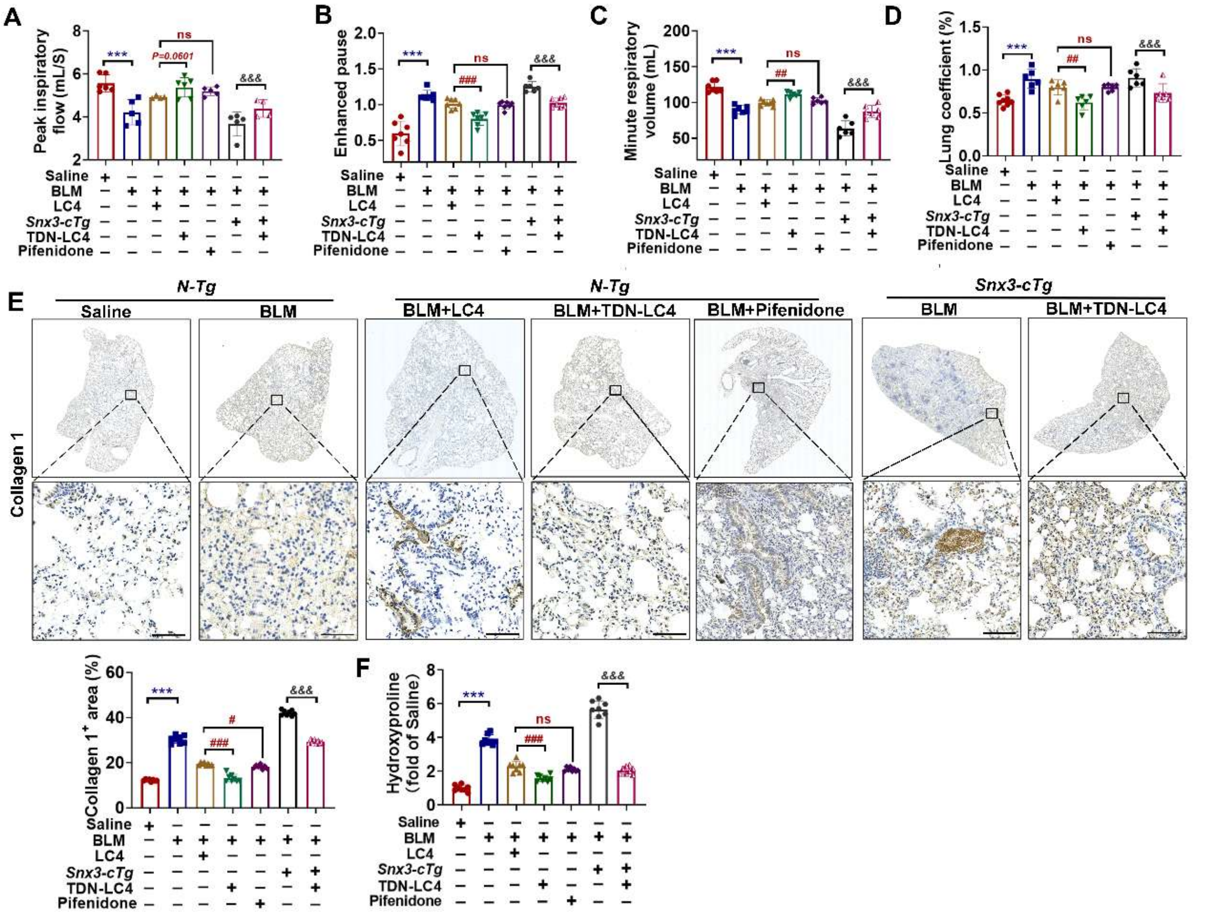

Figure S24 TDN-LC4 targeting SNX3 ameliorated pulmonary dysfunction and fibrosis, related to Figure 8.

(A-C), Peak inspiratory flow, Enhanced paused and Minute respiratory reduced in BLM-induced mice were measured by EMKA system; *n*=8 mice. **(D)**, The lung coefficient ratio was calculated; *n*=8 mice. **(E)**, Representative images of IHC staining analysis for Collagen 1 in lung tissue sections as indicated (Scale bar: 200 μm; *n*=8 mice). **(F)**, Representative images of hydroxyproline concentration were calculated, *n*=8 mice. The data were shown as means ± SEM. $^{*}P < 0.05$ vs. *Saline.* $^{\#}P < 0.05$ vs *BLM+LC4* group. $^{\&}P < 0.05$ vs *Snx3-cTg+BLM* group. ns, not significant.

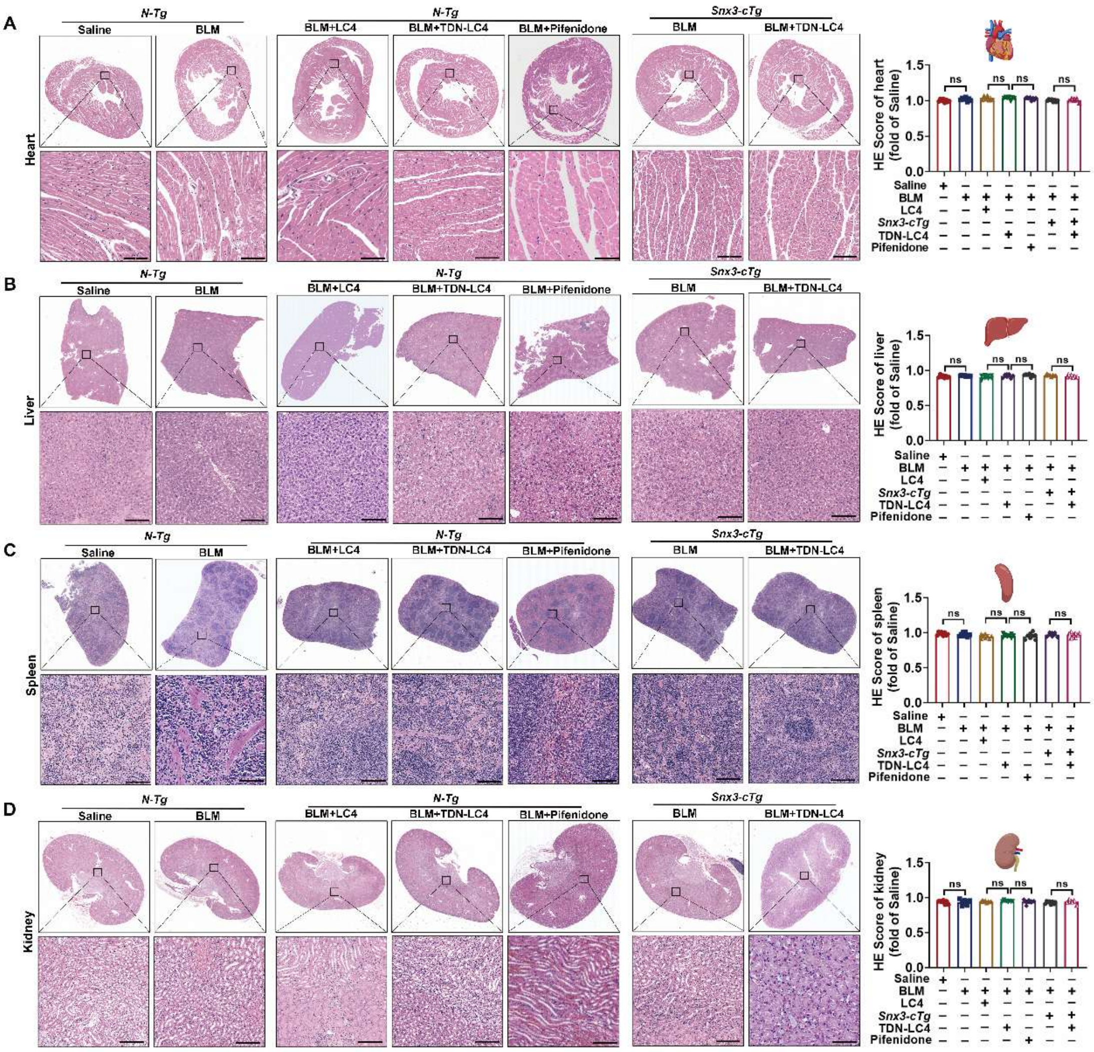

Figure S25 Safety evaluation of TDN-LC4 *in vivo*, related to Figure 8.

(A-D), Histopathological examination with H&E staining of heart, liver, spleen and kidney tissues obtained from TDN-LC4 treated mice; Scale bar: 200 μm, n=8 mice. The data were shown as means ± SEM. *$P < 0.05$ vs. *Saline.* $^{\#}P < 0.05$ vs *BLM+LC4* group. $^{\&}P < 0.05$ vs *Snx3-cTg+BLM* group. ns, not significant.

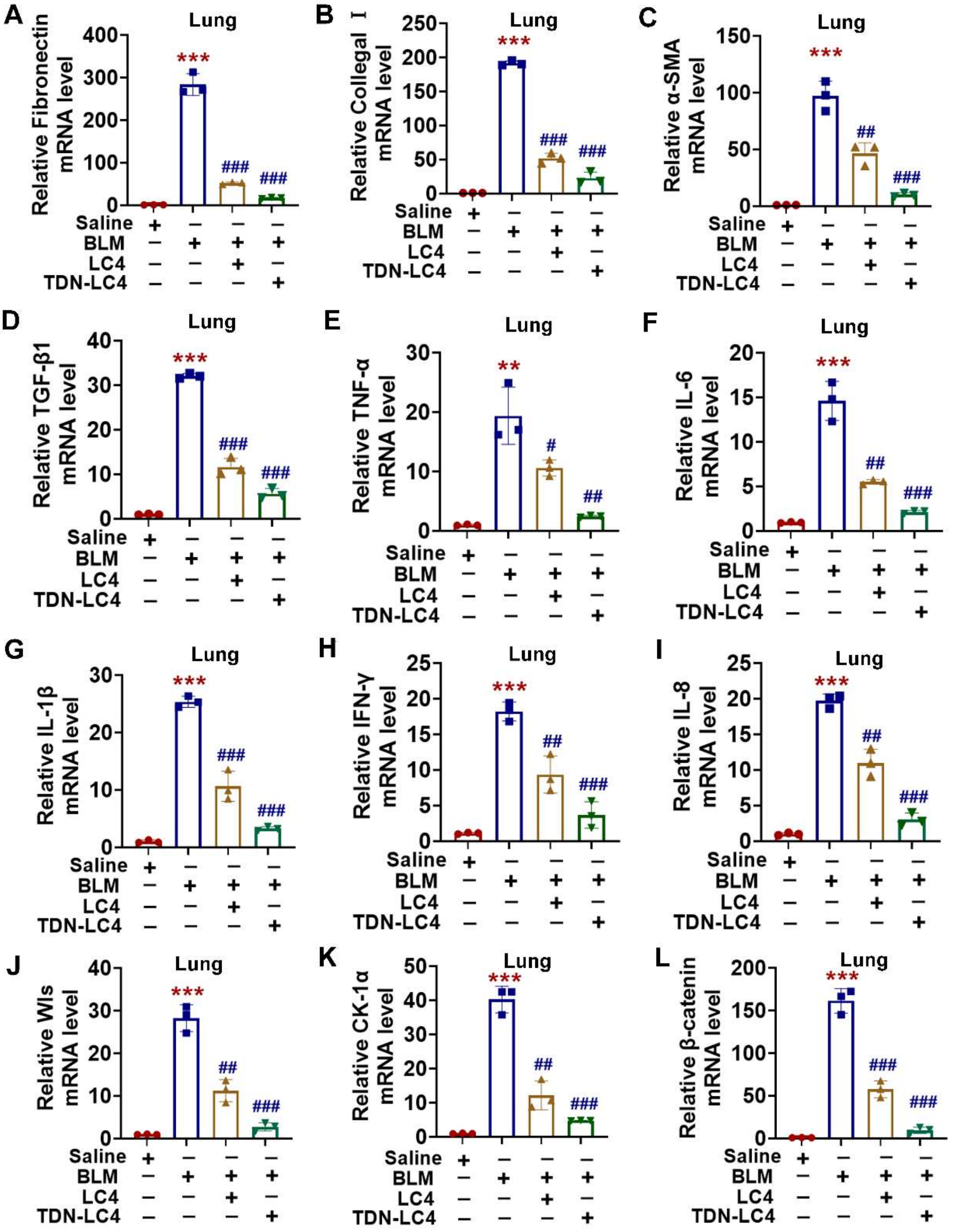

Figure S26 Safety evaluation of TDN-LC4 *in vivo*, related to Figure 8.
qPCR analysis of lung tissues from BLM-treated mice with versus without TDN-LC4 treatment. **(A-D)**, Pulmonary mRNA expression of fibrosis markers: Fibronectin, collagen I, α-SMA, TGF-β1. **(E-I)**, The mRNA expression of inflammatory cytokines and chemokines: TNF-α, IL-6, IL-1β, IFN-γ, IL-8. **(J-L)**, The mRNA expression of Wnt/β-catenin pathway: Wls, CK-1α, β-catenin. *n*=8 mice. Data were shown as means ± SEM. ns, not significant.

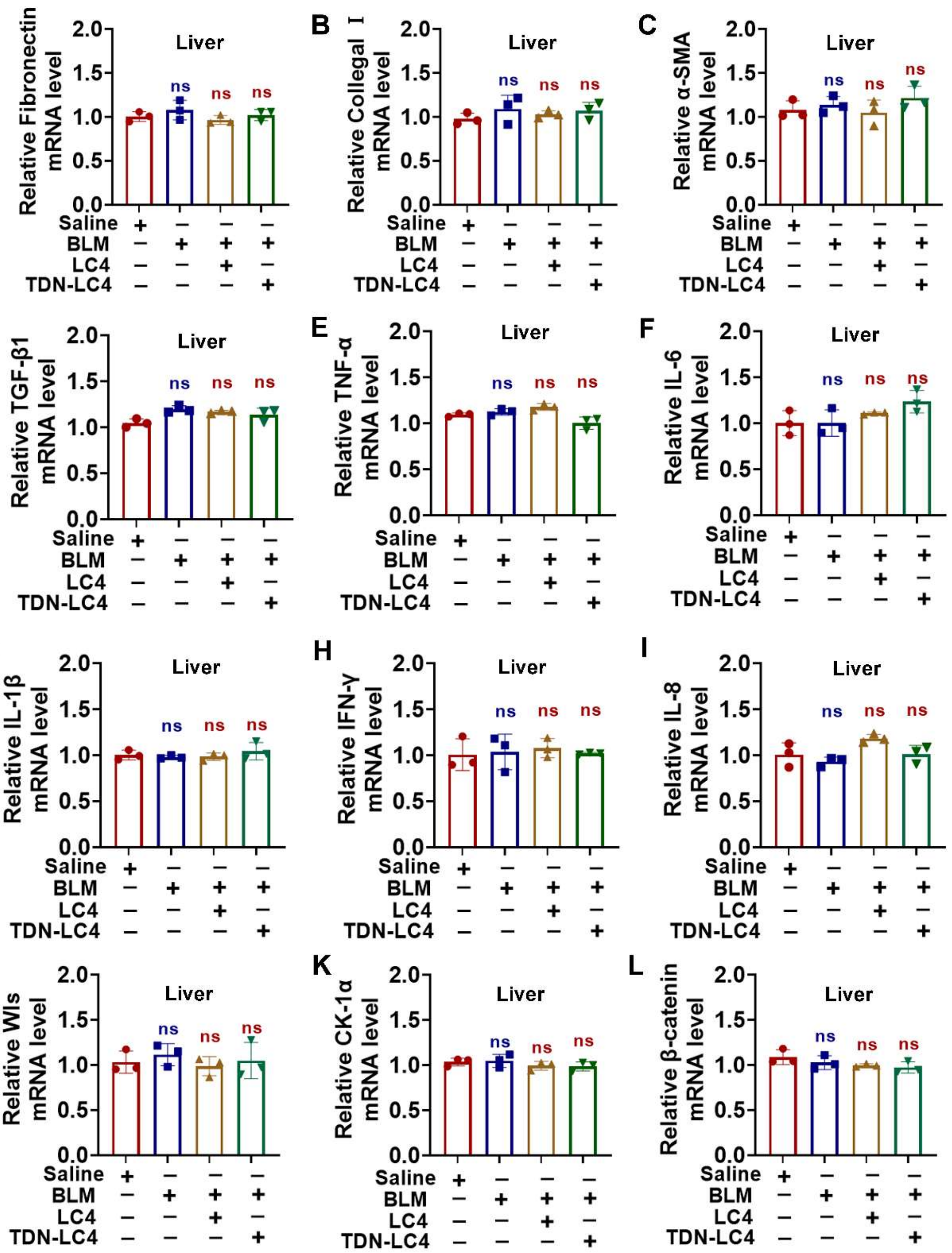

Figure S27 Evaluation of hepatic off-target impacts of TDN-LC4 in BLM-induced pulmonary fibrosis, related to Figure 8.

qPCR analysis of liver tissues from BLM-treated mice with versus without TDN-LC4 treatment. **(A-D)**, Hepatic mRNA expression of fibrosis markers: Fibronectin, collagen I, α-SMA, TGF-β1. **(E-I)**, The mRNA expression of inflammatory cytokines and chemokines: TNF-α, IL-6, IL-1β, IFN-γ, IL-8. **(J-L)**, The mRNA expression of Wnt/β-catenin pathway: Wls, CK-1α, β-catenin. n=8 mice. Data were shown as means ± SEM. ns, not significant.

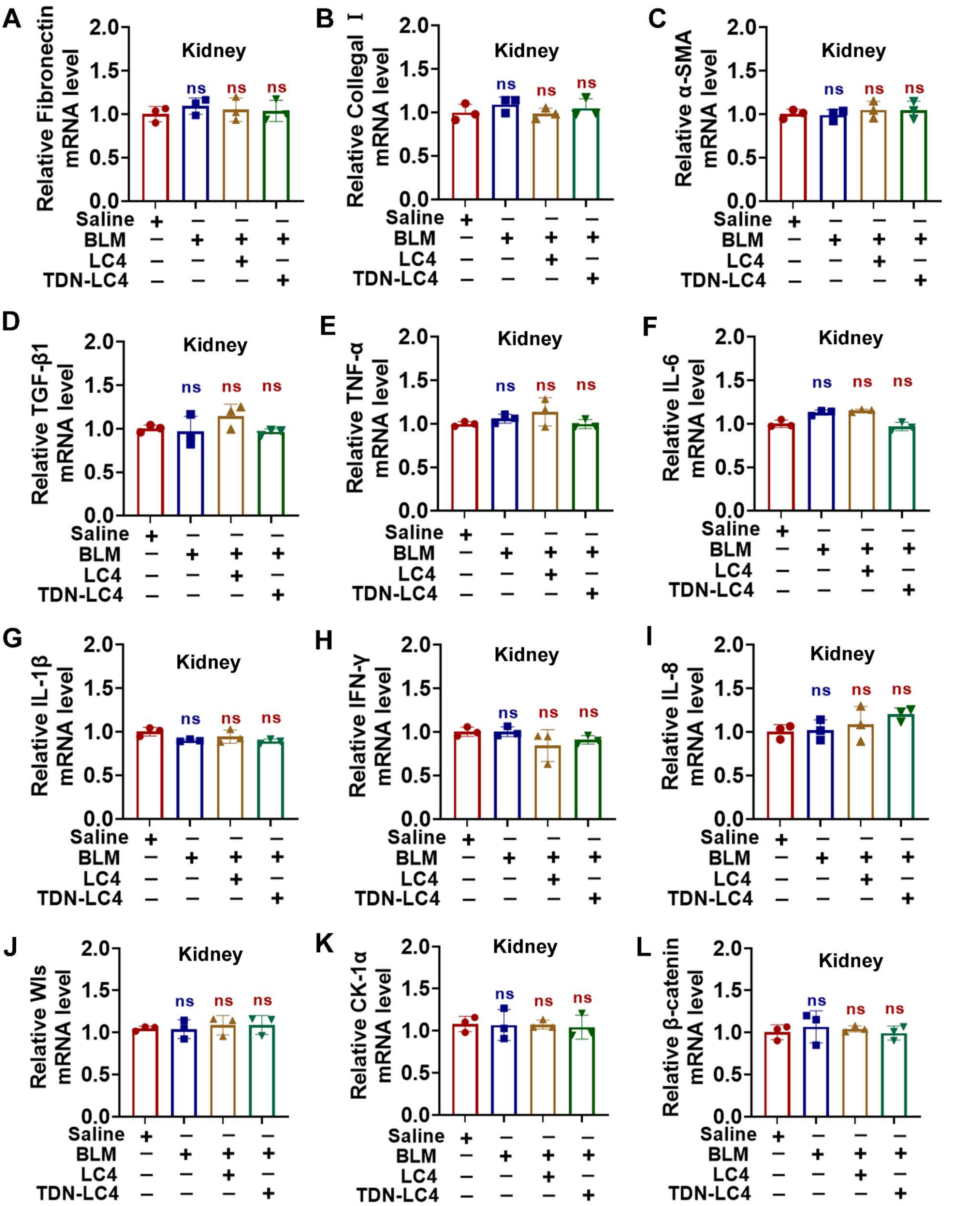

Figure S28 Evaluation of kidney off-target impacts of TDN-LC4 in BLM-induced pulmonary fibrosis, related to Figure 8.

qPCR analysis of kidney tissues from BLM-treated mice with versus without TDN-LC4 treatment. **(A-D)**, Renal mRNA expression of fibrosis markers: Fibronectin, collagen I, α-SMA, TGF-β1. **(E-I)**, The mRNA expression of inflammatory cytokines and chemokines: TNF-α, IL-6, IL-1β, IFN-γ, IL-8. **(J-L)**, The mRNA expression of Wnt/β-catenin pathway: Wls, CK-1α, β-catenin. *n*=8 mice. Data were shown as means ± SEM. ns, not significant.

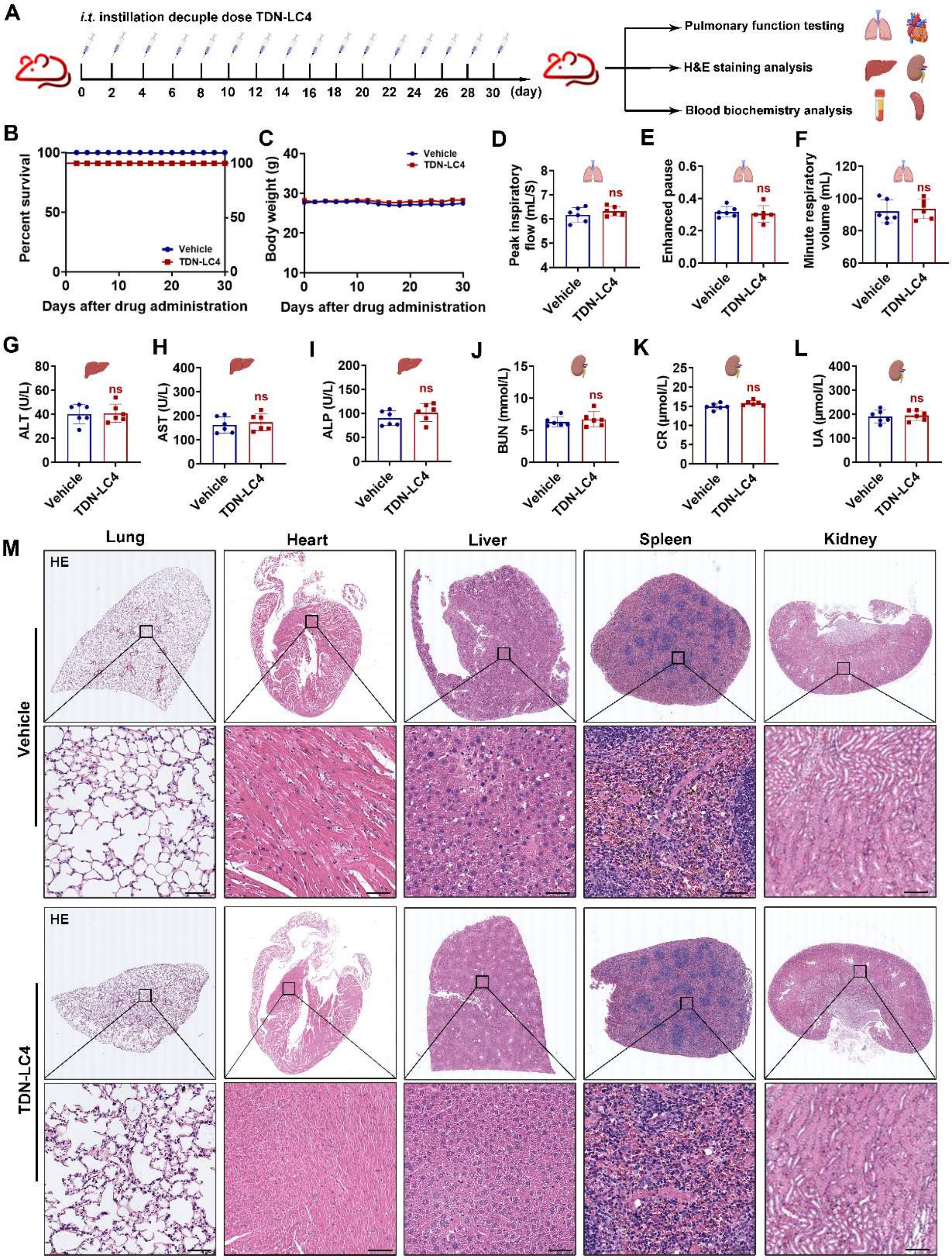

Figure S29 Toxicity assessment of TDN-LC4 at 10-fold the therapeutic dose in healthy mice, related to Figure 8.

(A), Schematic diagram of the experimental design for the chronic toxicity study of TDN-LC4. **(B)**, Survival rate of mice following administration of TDN-LC4. **(C)**, Body weight changes throughout the study period. **(D-F)**, Pulmonary function parameters, including peak inspiratory flow, enhanced pause, minute respiratory volume. **(G-I)**, Serum biochemical markers of hepatic

function, including alanine aminotransferase (ALT), aspartate aminotransferase (AST), alkaline phosphatase (ALP). **(J-L)**, Serum biochemical markers of renal function, including blood urea nitrogen (BUN), creatinine (CR), uric acid (UA). **(M)**, Representative hematoxylin and eosin (H&E)-stained histological sections from major organs (lungs, heart, liver, spleen, kidneys). Scale bars: 200 µm; n=6 mice. Data were shown as means ± SEM. *$P < 0.05$ *vs.* TDN-LC4 group. ns, not significant.

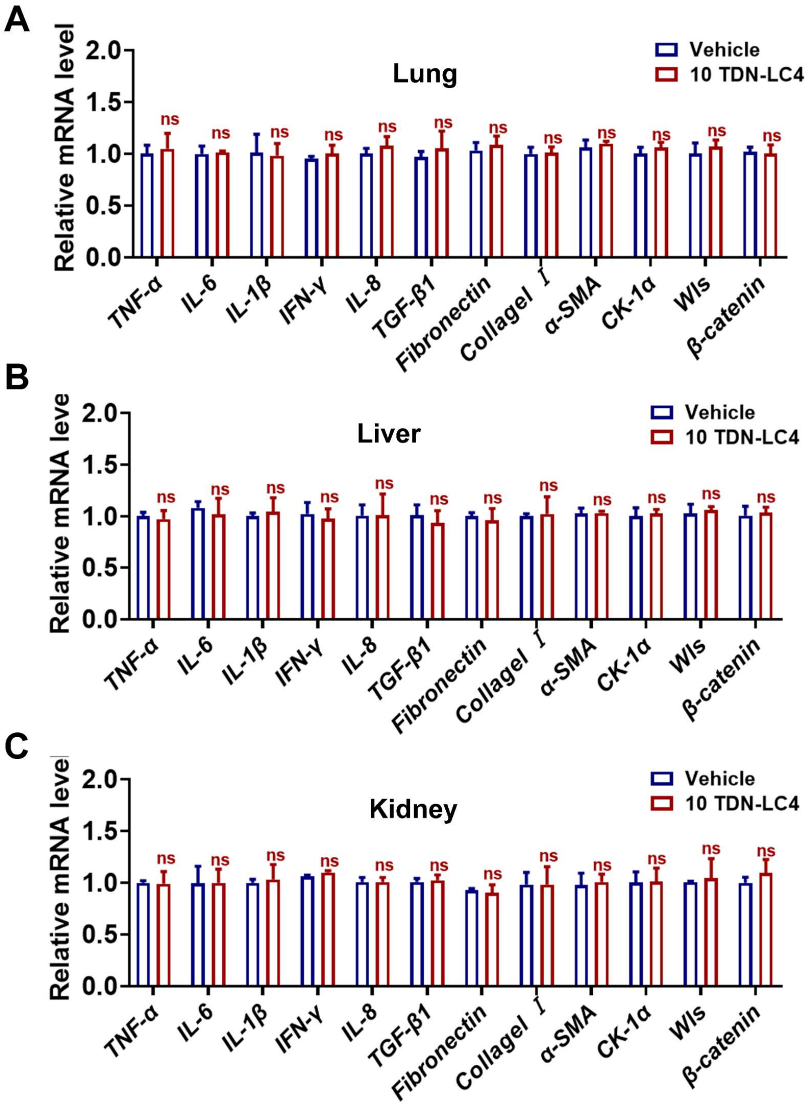

Figure S30. Evaluation of off-target effects of TDN-LC4 on major organs at 10-fold therapeutic dose, related to Figure 8.

qPCR analysis of liver and kidney tissues were measured. **(A)**, Pulmonary mRNA expression of inflammatory cytokines and chemokines (TNF-α, IL-6, IL-1β, IFN-γ, IL-8), fibrosis markers (TGF-β1, Fibronectin, collagen I, α-SMA), and Wnt/β-catenin pathway (CK-1α, Wls, β-catenin). **(B)**, Hepatic mRNA expression of inflammatory cytokines and chemokines (TNF-α, IL-6, IL-1β, IFN-γ, IL-8), fibrosis markers (TGF-β1, Fibronectin, collagen I, α-SMA), and Wnt/β-catenin pathwayy (CK-1α, Wls, β-catenin). **(C)**, Renal mRNA expression of inflammatory cytokines and chemokines (TNF-α, IL-6, IL-1β, IFN-γ, IL-8), fibrosis markers (TGF-β1, Fibronectin, collagen I, α-SMA), and Wnt/β-catenin pathway (CK-1α, Wls, β-catenin). *n*=6 mice. Data were shown as means ± SEM. ns, not significant.

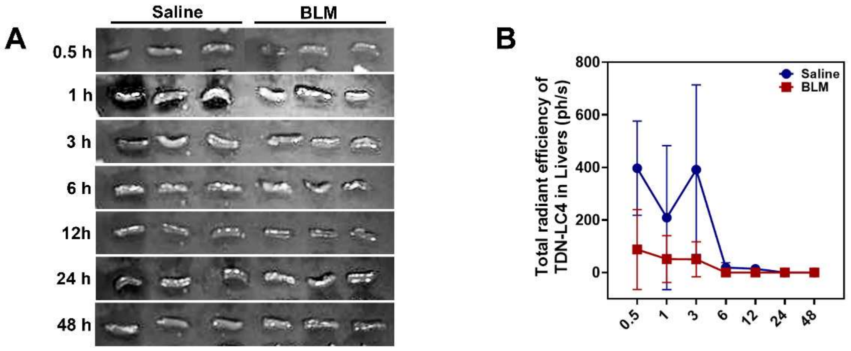

Figure S31 Pharmacokinetics of TDN-LC4 following intratracheal instillation in mice, related to Figure 9.

(A), Renal fluorescence intensity of Cy5-labeled TDN-LC4 in healthy and BLM-challenged mice at serial timepoints post-administration. **(B)**, Hepatic retention of TDN-LC4 in healthy and BLM-challenged mice at serial timepoints post-administration. Error bars represent SEM (n=6 mice).
