# Supplementary material for: Targeting sorting nexin 3 to treat pulmonary fibrosis by dual modulating Wnt/β-catenin signaling
Source: Cell Death Dis. 2026 Jan 15;17(1):43. doi: 10.1038/s41419-025-08248-x (PMC12808692; doi:10.1038/s41419-025-08248-x)

Figure 1 and Supplementary FigureS1-S3

Figure 1F

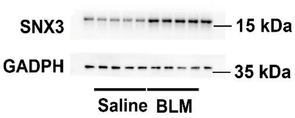

Figure S1F

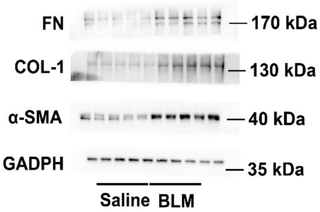

Figure S2C

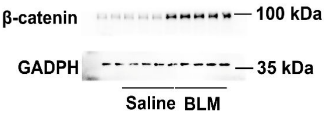

Figure S3B

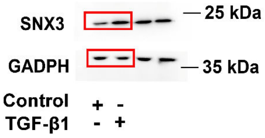

Figure S3D

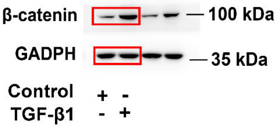

Figure S3E

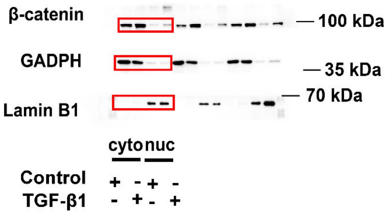

Figure S3J

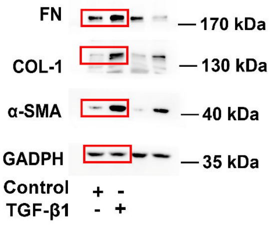

Figure 2 and Supplementary FigureS1-S3

Figure 2B

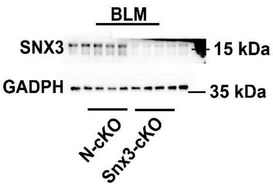

Figure 2K

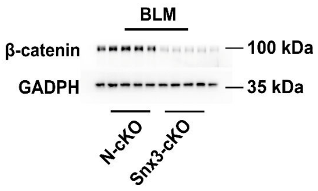

Figure 2M

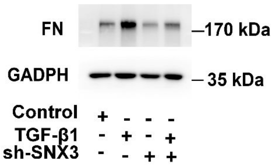

Figure 2O

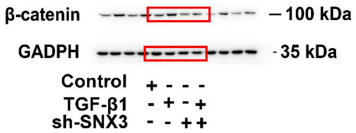

Figure 2P

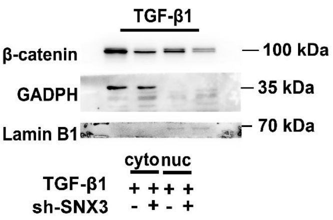

Figure S4R

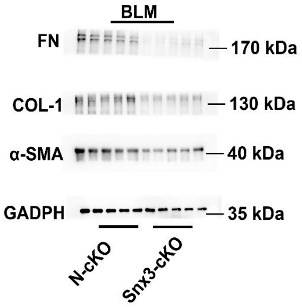

Figure S5A

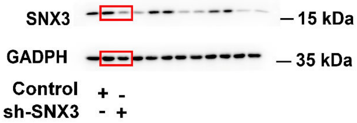

Figure S5F

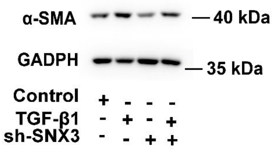

Figure S5G

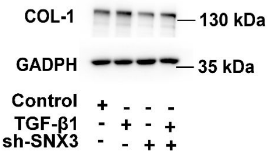

Figure 3 and Supplementary FigureS7-S8

Figure 3B

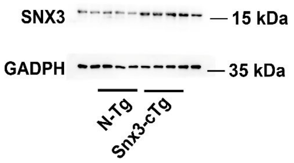

Figure 3K

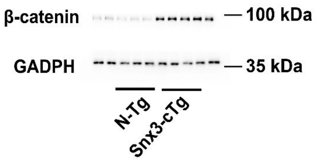

Figure 3N

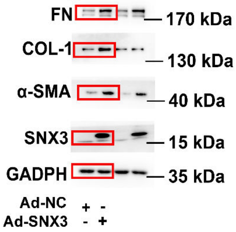

Figure S7O

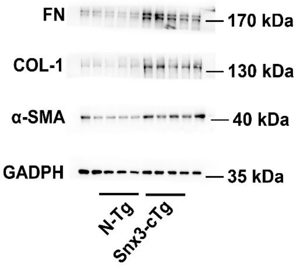

Figure S8C

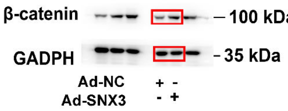

Figure S8D

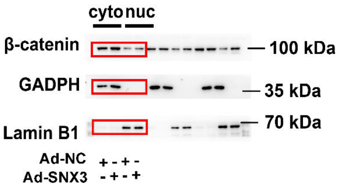

Figure 4 and Supplementary FigureS10-S12,S14

Figure 4B

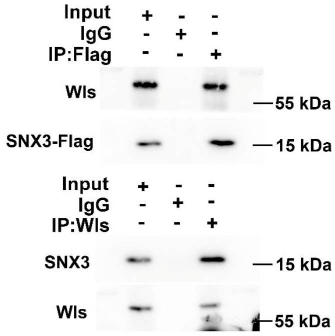

Figure S10B

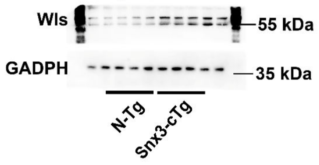

Figure S11F

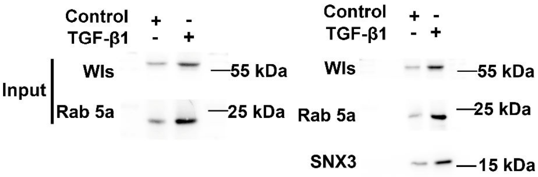

Figure S11G

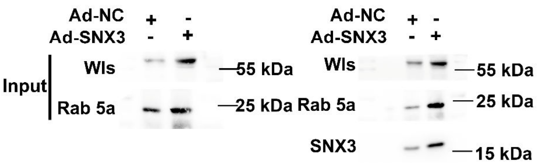

Figure S12F

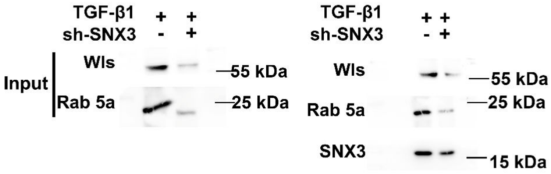

Figure S14B

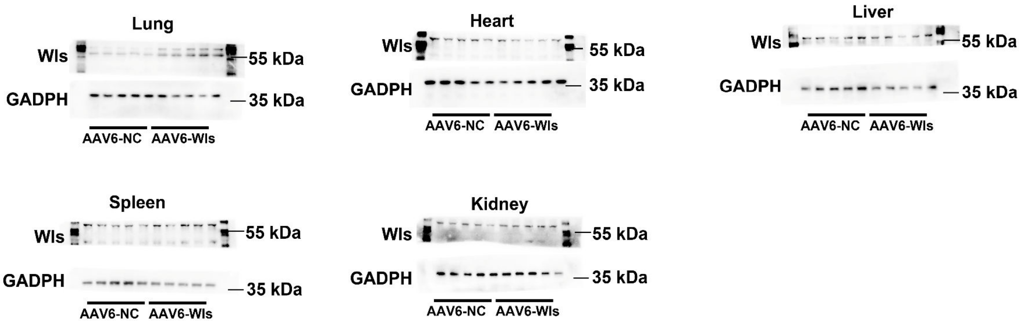

Figure 5 and Supplementary FigureS16-S17,S19-S20

Figure 5C

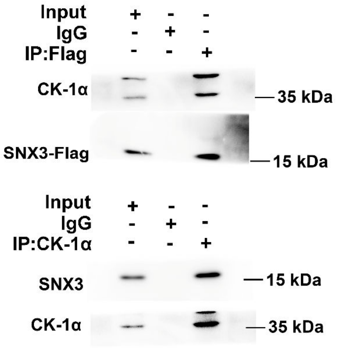

Figure S16E

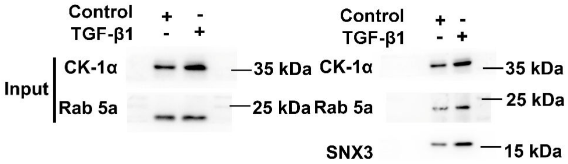

Figure S16F

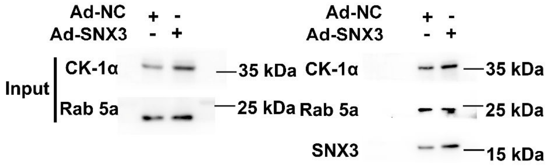

Figure S17F

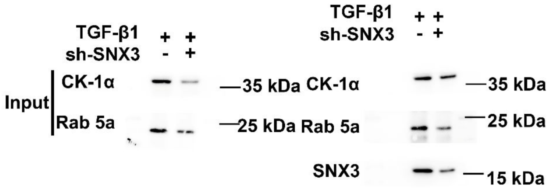

Figure S20B

Figure S19A

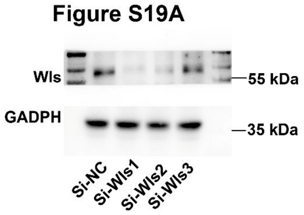

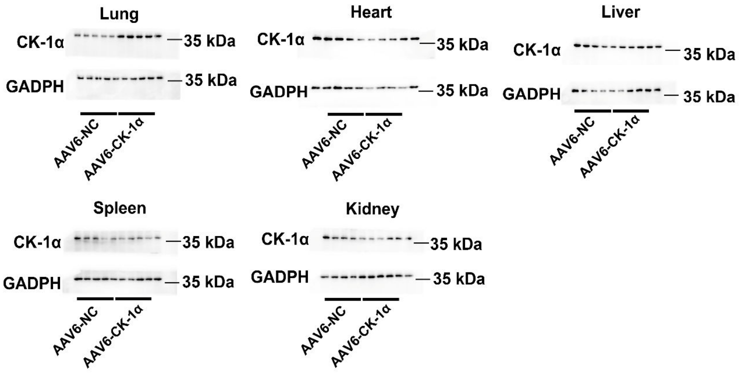

Figure 7

Figure 7D

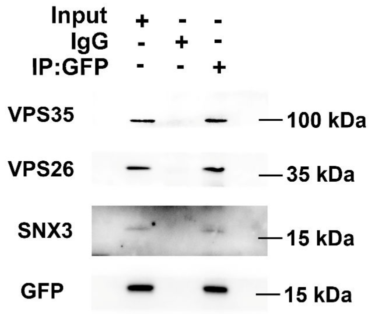

Supplement: Supplementary file 2 — Related Manuscript File [file 41419_2025_8248_MOESM2_ESM.pdf]
